# Crystal structures of two heterotrimetallic dysprosium–manganese–sodium 12-metallacrown-4 complexes with the bridging ligands 3-hy­droxy­benzoate and 4-hy­droxy­benzoate

**DOI:** 10.1107/S2056989020008853

**Published:** 2020-07-07

**Authors:** Elizabeth C. Manickas, Matthias Zeller, Curtis M. Zaleski

**Affiliations:** a Department of Chemistry and Biochemistry, Shippensburg University, Shippensburg, PA 17257, USA; b Department of Chemistry, Purdue University, West Lafayette, IN 47907, USA

**Keywords:** heterotrimetallic, metallacrown, self-assembled coordination complex, crystal structure

## Abstract

The syntheses and crystal structures for the compounds tetra-μ-aqua-­tetra­kis­{2-[aza­nid­yl­ene(oxido)meth­yl]phenolato}tetra­kis­(μ_2_-3-hy­droxy­benzoato)dys­pro­s­ium(III)­tetra­manganese(III)sodium(I) *N*,*N*-di­methyl­acetamide deca­solvate, [DyMn_4_Na(C_7_H_5_O_3_)_4_(C_7_H_4_NO_2_)_4_(H_2_O)_4_]·10C_4_H_9_NO or [Dy^III^Na(4-OHben)_4_{12-MC_Mn(III)N(shi)_-4}(H_2_O)_4_]·10DMA, **1**, and tetra-μ-aqua-­tetra­kis­{2-[aza­nid­yl­ene(oxido)meth­yl]phenolato}tetra­kis­(μ_2_-3-hy­droxy­benzoato)dys­pros­ium(III)tetra­manganese(III)sodium(I) *N*,*N*-di­methylformamide tetra­solvate, [DyMn_4_Na(C_7_H_5_O_3_)_4_(C_7_H_4_NO_2_)_4_(H_2_O)_4_]·4C_3_H_7_NO or [Dy^III^Na(3-OHben)_4_{12-MC_Mn(III)N(shi)_-4}(H_2_O)_4_]·4DMF, **2**, and where MC is metallacrown, shi^3−^ is salicyl­hydroximate, 3-OHben is 3-hy­droxy­benzoate, DMA is *N*,*N*-di­methyl­acetamide, 4-OHben is 4-hy­droxy­benzoate, and DMF is *N*,*N*-di­methyl­formamide, are reported. For both **1** and **2**, the macrocyclic metallacrown consists of an [Mn^III^—N—O] ring repeat unit, and the domed metallacrown captures two ions in the central cavity: a Dy^III^ ion on the convex side of the metallacrown and an Na^+^ ion the concave side. The Mn^III^ ions are six-coordinate with an elongated tetra­gonally distorted octa­hedral geometry. Both the Dy^III^ and Na^+^ ions are eight-coordinate. The Dy^III^ ions possess a square-anti­prismatic geometry, while the Na^+^ ions have a distorted biaugmented trigonal–prismatic geometry. Four 3-hy­droxy­benzoate or 4-hy­droxy­benzoate ligands bridge each Mn^III^ ion to the central Dy^III^ ion. For **1**, whole-mol­ecule disorder is observed for the main mol­ecule, excluding only the Dy^III^ and Na^+^ ions, and the occupancy ratio refined to 0.8018 (14):0.1982 (14). Three DMA mol­ecules were refined as disordered with two in general positions by an approximate 180° rotation and the third disordered twice by general disorder as well as by an exact 180° rotation about a twofold axis that bis­ects it. The occupancy ratios refined to 0.496 (8):0.504 (8), 0.608 (9):0.392 (9), and 2×0.275 (7):2×0.225 (7), respectively. For **2**, segments of the metallacrown are disordered including the Dy^III^ ion, one of the Mn ions, two of the Mn-bound 4-hy­droxy­benzoate ligands, the Mn-bridging salicyl­hydroximate ligand, and portions of the remaining three shi^3−^ ligands. The occupancy ratio for the metallacrown disorder refined to 0.849 (9):0.151 (9). Two DMF solvent mol­ecules are also disordered, each over two orientations. The disorder ratios refined to 0.64 (3):0.36 (3) and to 0.51 (2):0.49 (2), respectively. For **2**, the crystal under investigation was refined as a non-merohedric twin by a 90° rotation around the real *a* axis [twin ratio 0.9182 (8):0.0818 (8)].

## Abstract 2

The metallacrown complexes Dy^III^Na(3-OHben)_4_[12-MC_Mn(III)N(shi)_-4](H_2_O)_4_·10DMA, **1**, and Dy^III^Na(4-OHben)_4_[12-MC_Mn(III)N(shi)_-4](H_2_O)_4_·4DMF, **2**, where MC is metallacrown, shi^3−^ is salicyl­hydroximate, 3-OHben is 3-hy­droxy­benzoate, DMA is *N*,*N*-di­methyl­acetamide, 4-OHben is 4-hy­droxy­benzoate, and DMF is *N*,*N*-di­methyl­formamide, consist of a macrocyclic mol­ecule with an [Mn^III^—N—O] repeat unit. For both **1** and **2**, a Dy^III^ ion is captured on the convex side of the central cavity, while a Na^+^ ion is captured on the concave side of the cavity. Four 3-hy­droxy­benzoate or 4-hy­droxy­benzoate anions bridge between the ring Mn^III^ ions and the central Dy^III^ ion.

## Chemical context   

Metallacrowns (MC) were first discovered in 1989 by Pecoraro, and the compounds have grown into a class of coordination complexes with a wide range of applications including single-mol­ecule magnets, magnetorefrigerants, luminescent agents, cell imaging agents, and magnetic resonance imaging agents (Mezei *et al.*, 2007 ▸; Nguyen & Pecoraro, 2017 ▸; Lutter *et al.*, 2018 ▸; Anthanasopoulou *et al.*, 2018 ▸). MCs, the inorganic equivalent of crown ethers, are macrocyclic mol­ecules that follow a metal–nitro­gen–oxygen [*M*–N–O] repeat in the ring of the mol­ecule, similar to the carbon–carbon–oxygen [C–C–O] repeat of a crown ether. The self-assembly synthetic strategy of MCs lends itself to the ability to place metal ions in specific positions in the mol­ecules and the controllable formation of specific mol­ecules. While heterobimetallic MCs have been known since the 1990s, heterotrimetallic MCs have only been recently reported (Azar *et al.*, 2014 ▸; Travis *et al.*, 2015 ▸, 2016 ▸; Cao *et al.*, 2016 ▸; Boron *et al.*, 2016 ▸; Lutter *et al.*, 2020 ▸). These structures are based on a 12-MC-4 framework with manganese(III) or gallium(III) as the ring metal, a central lanthanide ion, and typically an alkali metal ion bound opposite to the lanthanide ion – though in one case a tungsten(V) ion is bound opposite the lanthanide ion. In general, the controllable formation of heterotrimetallic systems remains difficult from a synthetic perspective; however, MCs provide a pathway that demonstrates that such systems are achievable in a straightforward and predictable fashion.

In 2014 we reported a series of *Ln* ^III^Na(OAc)_4_[12-MC_Mn(III)N(shi)_-4](H_2_O)_4_ complexes, where *Ln* ^III^ is Pr^III^ to Yb^III^ (except Pm^III^) and Y^III^, ^−^OAc is acetate, and shi^3−^ is salicyl­hydroximate, that were the first heterotrimetallic MCs and the first 12-MC-4 complexes to bind a lanthanide ion in the central cavity (Azar *et al.*, 2014 ▸). The lanthanide ion is tethered to the MC *via* four acetate bridges that link the central *Ln* ^III^ to the ring Mn^III^ ions. Since then we have reported other *Ln* ^III^Na(*X*)_4_[12-MC_Mn(III)N(shi)_-4] complexes, where *Ln* ^III^ is Y^III^, Er^III^, and Dy^III^, and *X* ^−^ is 2-hy­droxy­benzoate, benzoate, and tri­methyl­acetate, which demonstrate that the bridging carboxyl­ate anion can be easily substituted in these structures (Travis *et al.*, 2015 ▸, 2016 ▸; Boron *et al.*, 2016 ▸). In addition, the identity of the bridging ligand affects the single-mol­ecule magnet (SMM) properties of a series of [12-MC_Mn(III)N(shi)_-4] complexes with Dy^III^ as the central lanthanide ion (Boron *et al.*, 2016 ▸). Specifically, the p*K*_a_ value of the parent acid of the bridging ligand, which indicates the Lewis basicity of the anion, directly impacts the SMM behavior of the MCs. Only the 2-hy­droxy­benzoate (*i.e*. salicylate) version of the MCs behaves as an SMM, while the benzoate, acetate, and tri­methyl­acetate analogues do not possess any SMM behavior. 2-Hy­droxy­benzoic acid has the smallest p*K*_a_ value (2.98) of the species investigated, and the subsequent p*K*_a_ values increase from benzoic acid (4.20) to acetic acid (4.76) to tri­methyl­acetic acid (5.03). Thus, 2-hy­droxy­benzoate is the most electron-withdrawing of the set of anions, and this could affect the magnetic coupling between the ring Mn^III^ ions and central Dy^III^ ion.

Herein we report the syntheses and crystal structures of Dy^III^Na(3-OHben)_4_[12-MC_Mn(III)N(shi)_-4](H_2_O)_4_·10DMA, **1**, and Dy^III^Na(4-OHben)_4_[12-MC_Mn(III)N(shi)_-4](H_2_O)_4_·4DMF, **2**, where 3-OHben is 3-hy­droxy­benzoate, DMA is *N*,*N*-di­methyl­acetamide, 4-OHben is 4-hy­droxy­benzoate, and DMF is *N*,*N*-di­methyl­formamide. The p*K*_a_ values of 3-hy­droxy­benzoic acid and 4-hy­droxy­benzoic acid are 4.08 and 4.57, respectively, which are greater than the p*K*_a_ of 2-hy­droxy­benzoic acid. Future studies will investigate the magnetic properties of **1** and **2** and the impact of the identity of the bridging ligand on the single-mol­ecule magnetism of the MCs.
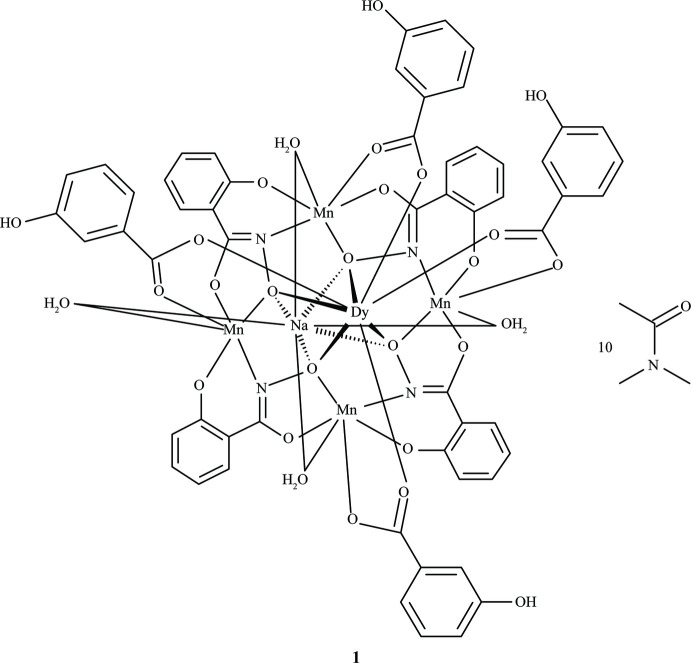
 
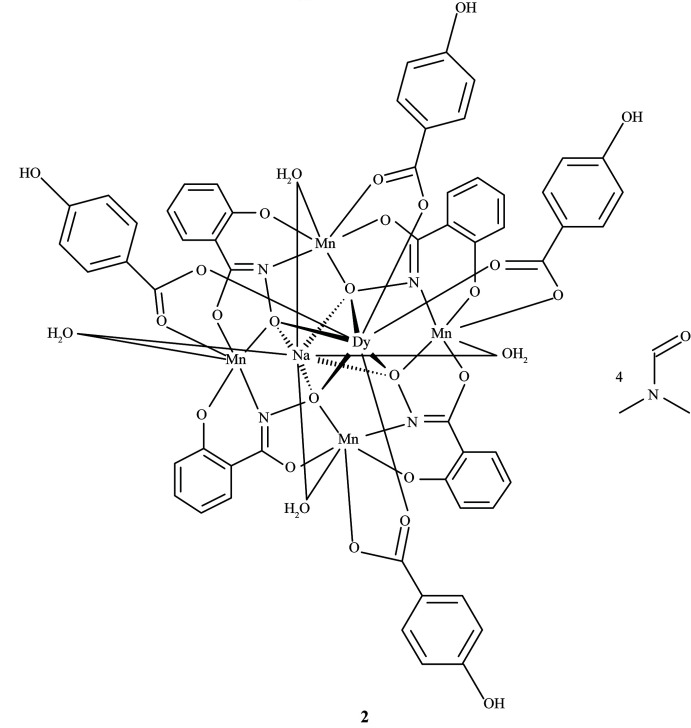


## Structural commentary   

**Figure 1:**
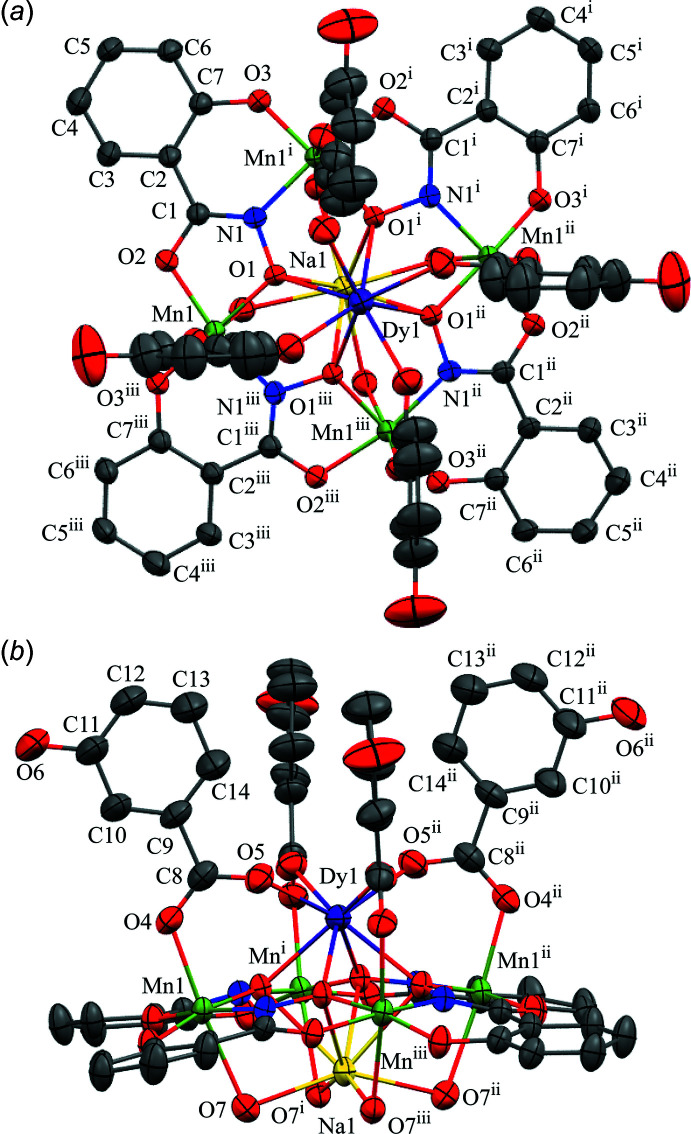
The single-crystal X-ray structure of Dy^III^Na(3-OHben)_4_[12-MC_Mn(III)N(shi)_-4](H_2_O)_4_·10DMA, **1**, (*a*) top view with only the metal atoms and shi^3−^ ligands labeled for clarity and (*b*) side view with only the metal atoms and axial ligands labeled for clarity. The displacement ellipsoids are drawn at the 50% probability level. For clarity, hydrogen atoms, solvent mol­ecules, and disorder have been omitted. Color scheme: purple – Dy^III^, green – Mn^III^, yellow – Na^+^, red – oxygen, blue – nitro­gen, and gray – carbon. All figures were generated with the program *Mercury* (Macrae *et al.*, 2020 ▸). [Symmetry codes: (i) +*x*, −*y* + 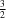, +*z*; (ii) −*x* + 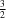, −*y* + 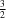, +*z*; (iii) −*x* + 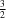, +*y*, +*z.*]

The metallacrown complexes Dy^III^Na(3-OHben)_4_[12-MC_Mn(III)N(shi)_-4](H_2_O)_4_·10DMA, **1**, and Dy^III^Na(4-OHben)_4_[12-MC_Mn(III)N(shi)_-4](H_2_O)_4_·4DMF, **2**, both possess the typical 12-MC-4 framework with a repeat unit of Mn^III^–N–O that recurs four times to generate an approximately square-shaped mol­ecule (Figs. 1 ▸ and 2 ▸). Each MC contains one Dy^III^ ion, one Na^+^ ion, and four Mn^III^ ions, which provides a total 16+ charge. This positive charge is counterbalanced by the four shi^3−^ ligands and four carboxyl­ate anions of the MCs (total 16− charge). Beyond overall mol­ecular charge considerations, the metal oxidation states are confirmed by average bond lengths and bond-valence sum (BVS) values (Table 1 ▸; Liu & Thorp, 1993 ▸ and Trzesowska *et al.*, 2004 ▸). The four Mn^III^ ions and four shi^3−^ ligands provide an MC framework that is able to bind the two central ions. The oxime oxygen atoms of the shi^3−^ ligands form the central MC cavity that binds Dy^III^ and Na^+^ ions on opposite faces of the MC. The metallacrown is slightly domed with the Dy^III^ ion bound to the convex side of the MC cavity and the Na^+^ ion attached to the concave side. As previously reported, the doming effect is likely due to the displacement of the ring metal atoms from the equatorial plane of the first coordination sphere ligand atoms (Azar *et al.*, 2014 ▸). For both **1** and **2**, the average distance of the Mn^III^ ions from the equatorial plane is 0.14 Å. The Dy^III^ ion is further attached to the MC *via* either four 3-hy­droxy­benzoate or 4-hy­droxy­benzoate anions that bridge between the Dy^III^ ion and each ring Mn^III^ ion. For **1** the mol­ecule possesses a fourfold rotation axis along the Dy^III^ and Na^+^ ions, and whole-mol­ecule disorder is observed for the main mol­ecule, excluding only the Dy^III^ and Na^+^ ions, with the occupancy ratio refined to 0.8018 (14):0.1982 (14). For **2**, large sections of the metallacrown are disordered, including the Dy^III^ ion, Mn1, two of the 4-hy­droxy­benzoate ligands bound to Mn1 and Mn2, the shi^3−^ ligand that connects Mn1 and Mn4, and portions of the remaining three shi^3−^ ligands. The occupancy ratio for the metallacrown disorder refined to 0.849 (9):0.151 (9). Complete details describing the treatment of the disorder are given in the *Refinement* section. The following structural descriptions focus only on the major disorder components.

For both **1** and **2**, each Mn^III^ ion is six-coordinate, with a tetra­gonally distorted octa­hedral geometry. The elongated Jahn–Teller axis along the *z* direction is expected for a high-spin *d* ^4^ electron configuration. The geometry assignment is supported by a continuous shape measures (CShM) analysis (*SHAPE 2.1*; Llunell *et al.*, 2013 ▸; Pinsky & Avnir, 1998 ▸). The CShM values of the Mn^III^ ions range from 1.115 to 1.434 (Table 2 ▸). Typically CShM values less than 1.0 indicate only minor distortions of the assigned geometry from the ideal shape (Cirera *et al.*, 2005 ▸), while CShM values up to 3.0 indicate significant distortions from the ideal geometry; however, a value up to 3.0 still represents an acceptable description of the geometry. The CShM values for the Mn^III^ ions are likely greater than 1.0 due to the presence of the Jahn–Teller axis. The elongated Jahn–Teller distortion is composed of a carboxyl­ate oxygen atom from a 3-hy­droxy­benzoate or 4-hy­droxy­benzoate anion and a bridging water mol­ecule that is also bound to the central Na^+^ ion. The equatorial donor atoms form two *trans* chelate rings about each Mn^III^ ion. A five-membered chelate ring is comprised of an oxime oxygen atom and a carbonyl oxygen atom from a shi^3−^ ligand, and a six-membered chelate ring is formed by an oxime nitro­gen atom and a phenolate oxygen atom from a different shi^3−^ ligand.

The central Dy^III^ ion on the convex side of the MC is eight-coordinate, with a distorted square anti­prismatic geometry (CShM values: 0.550 for **1** and 0.818 for **2**; Table 3 ▸; Casanova *et al.*, 2005 ▸). Two different planes of oxygen atoms complete the coordination sphere. One plane is composed of four oxime oxygen atoms from the MC cavity, while the second plane is formed from four carboxyl­ate oxygen atoms from either the 3-hy­droxy­benzoate or 4-hy­droxy­benzoate anions. The Dy^III^ lies closer to the mean plane of carboxyl­ate oxygen atoms [1.055 (3) Å for **1** and 1.076 (7) Å for **2**] than to the mean plane of oxime oxygen atoms [1.546 (3) Å for **1** and 1.593 (7) Å for **2**], indicating that the geometry is distorted from an ideal square anti­prism geometry. The mean plane distances were calculated with *SHELXL2018/3* (Sheldrick, 2015 ▸) and determined as previously described (Azar *et al.*, 2014 ▸).

The Na^+^ ion captured on the concave side of the MC is also eight-coordinate; however, the geometry assignment is not clearly defined based on CShM values (Table 3 ▸). The CShM analysis slightly favors a biaugmented trigonal–prismatic assignment (CShM values: 3.002 for **1** and 3.196 for **2**); however, a square-anti­prismatic geometry assignment is comparable (CShM values: 3.063 for **1** and 3.657 for **2**). Both values are above 3.0; thus, there are substantial distortions from each ideal geometry. The biaugmented trigonal–prismatic geometry is a trigonal prism capped on two of the three rectangular faces. As for the Dy^III^ ion, the Na^+^ ion is surrounded by two groups of oxygen atoms. One group of oxygen atoms is formed from the oxime oxygen atoms of the MC cavity, and the second group is comprised of four oxygen atoms from water mol­ecules. The Na^+^ ion is positioned closer to the mean plane of water oxygen atoms [0.677 (5) Å for **1** and 0.561 (9) Å for **2**] than to the mean plane of the oxime oxygen atoms [1.922 (4) Å for **1** and 1.991 (9) Å for **2**].

Lastly, in both **1** and **2** solvent mol­ecules are located in the structure, which are also hydrogen bonded to their respective MCs (described in the *Supra­molecular features* section). For **1**, the DMA mol­ecules associated with N2 and N3 are disordered over two positions with occupancy ratios that refined to 0.496 (8):0.504 (8) and 0.608 (9):0.392 (9), respectively. The DMA molecule associated with N4 is disordered over four positions with occupancy ratios that refined to 2×0.275 (7):2×0.225 (7). For **2**, two DMF mol­ecules associated with N6 and N7 are not disordered, while the two DMF mol­ecules associated with N5 and N8 are disordered over two different orientations, which refined to 0.64 (3):0.36 (3) and 0.51 (2):0.49 (2), respectively. Complete details describing the treatment of the solvent disorder are given in the *Refinement* section.

## Supra­molecular features   

**Figure 3:**
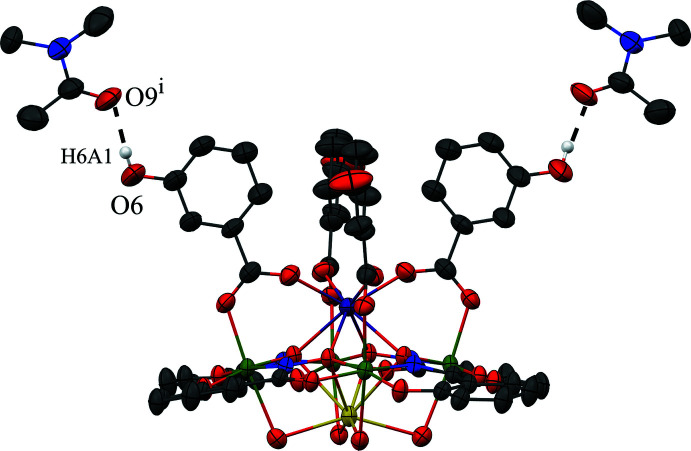
Inter­molecular hydrogen bonding between **1** and the carbonyl oxygen atom of a DMA mol­ecule. For clarity only the hydrogen atoms (white) involved in the inter­actions have been included, and only the atoms involved in the inter­actions have been labeled. See Fig. 1 ▸ for additional display details. [Symmetry code: (i) *x*, *y*, *z* − 1.]

**Figure 4:**
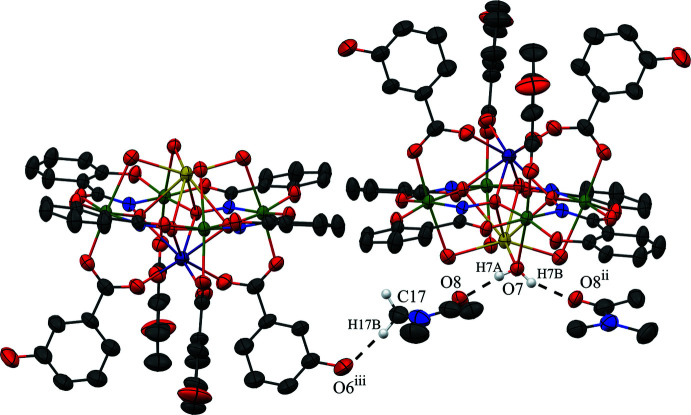
Inter­molecular hydrogen bonding between the water mol­ecule coordinated to the Na^+^ ion of **1** and the DMA mol­ecules. The DMA mol­ecules then form C—H⋯O inter­actions with the hydroxyl groups of 3-hy­droxy­benzoate anions of neighboring MCs to generate a network between the complexes. For clarity only the hydrogen atoms (white) involved in the inter­actions have been included, and only the atoms involved in the inter­actions have been labeled. See Fig. 1 ▸ for additional display details. [Symmetry codes: (ii) *y*, −*x* + 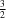, *z*; (iii) *y* − 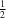, −*x* + 2, −*z* + 1.]

For both **1** and **2** the solvent mol­ecules form hydrogen bonds with the MC complexes. For **1**, the MC complex forms hydrogen bonds to the DMA mol­ecules, and the MCs are inter­connected *via* the DMA mol­ecules (Table 4 ▸). The hydroxyl group (O6) of each 3-hy­droxy­benzoate forms a hydrogen bond to the carbonyl oxygen atom (O9^i^) of a DMA mol­ecule [Fig. 3 ▸; symmetry code: (i) *x*, *y*, *z* − 1]. In addition, the water mol­ecule (O7) coordinated to the central Na^+^ ion hydrogen bonds to the carbonyl oxygen atoms (O8 and O8^ii^) of two DMA mol­ecules [Fig. 4 ▸; symmetry code: (ii) −*x* + 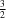, *y*, *z*]. Then, the methyl group (associated with C17) of the same DMA mol­ecules forms a C—H⋯O inter­action with the hydroxyl group (O6^iii^) of a 3-hy­droxy­benzoate of a neighboring MC [symmetry code: (iii) −*x* + 2, *y* − 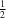, −*z* + 1]. These inter­actions are repeated about the fourfold axis of the MC; thus, a network is generated between neighboring MCs mediated by the DMA mol­ecule associated with N2. The connection between the neighboring MCs, the hydrogen bonds between the MCs and the DMA mol­ecules, and pure van der Waals forces contribute to the overall packing of the mol­ecules.

**Figure 5:**
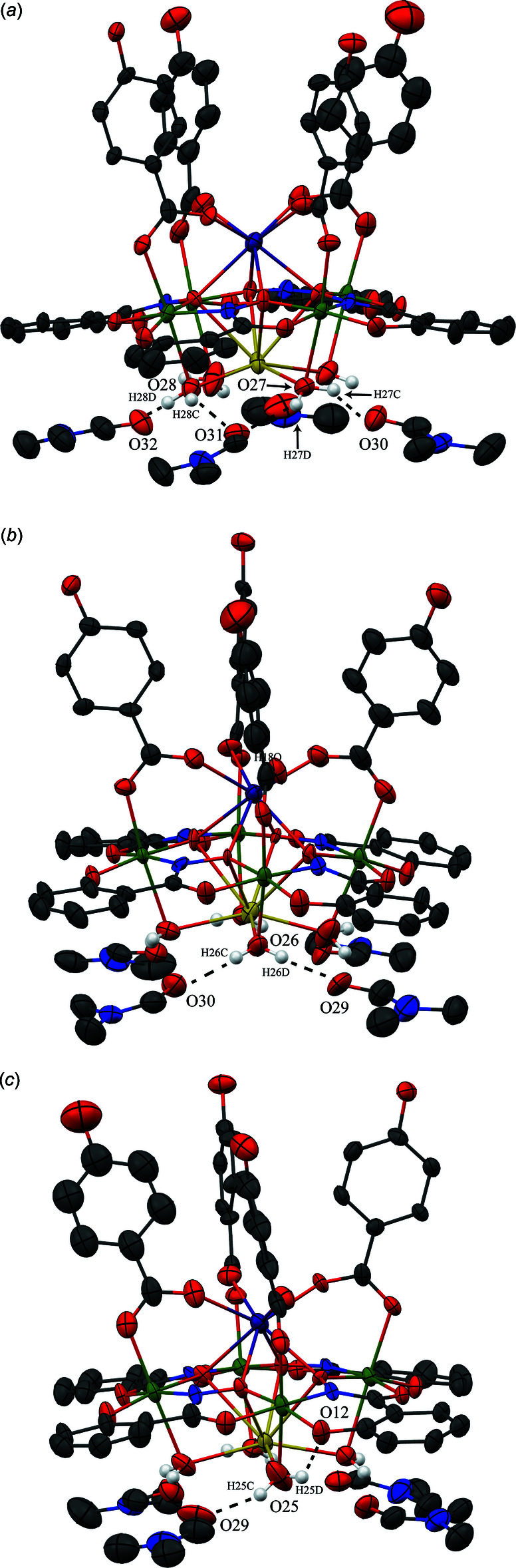
Inter­molecular hydrogen bonding between the water mol­ecules coordinated to the Na^+^ ion of **2** and the DMF mol­ecules and intra­molecular hydrogen bonding between a water mol­ecule coordinated to the Na^+^ ion and a phenolate oxygen atom of the metallacrown. For clarity the hydrogen bonding has been divided into three sections (*a*), (*b*) and (*c*), only the hydrogen atoms (white) involved in the hydrogen bonding have been included, and only the atoms involved in the hydrogen bonding have been labeled. See Fig. 1 ▸ for additional display details.

**Figure 6:**
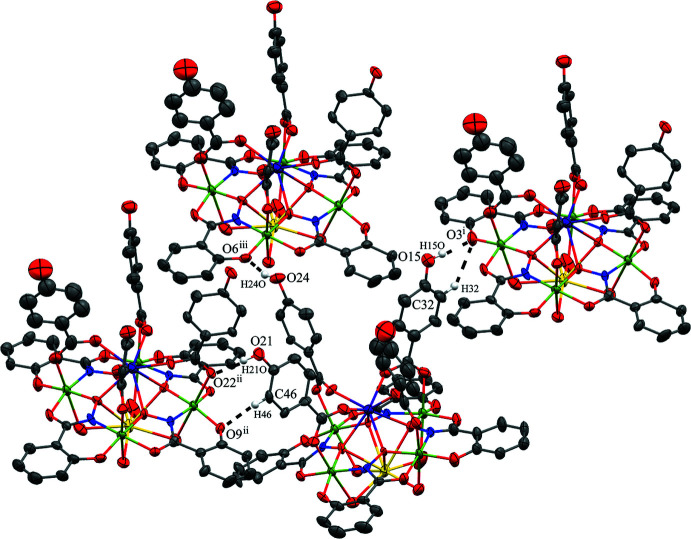
Inter­molecular hydrogen bonding between adjacent metallacrowns of **2**, which generate a network between the MCs. For clarity only the hydrogen atoms (white) involved in the inter­actions have been included, and only the atoms involved in the inter­actions have been labeled. See Fig. 1 ▸ for additional display details. [Symmetry codes: (i) *x* − 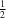, −*y* + 1, *z* + 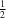; (ii) *x* − 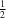, −*y* + 2, *z* − 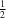; (iii) *x* − 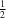, −*y* + 2, *z* + 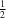.]

For **2**, several DMF mol­ecules are hydrogen bonded to each metallacrown and a small hydrogen-bonding network exists between neighboring metallacrowns (Table 5 ▸). The four water mol­ecules (O25–O28) coordinated to the central Na^+^ ion hydrogen bond to the carbonyl oxygen atoms of four DMF mol­ecules (Fig. 5 ▸). There is also one intra­molecular hydrogen bond between one of the water mol­ecules (O25) coordinated to the Na^+^ ion and a phenolate oxygen atom (O12) of the metallacrown (Fig. 5 ▸ *c*). In addition, several hydrogen bonds exist between neighboring metallacrowns (Fig. 6 ▸). The hydrogen bonding occurs *via* the 4-hy­droxy­benzoate ligands. The hydroxyl group (O15) of a 4-hy­droxy­benzoate anion forms a hydrogen bond to O3^i^ (a phenolate oxygen atom of a shi^3−^ ligand) of a neighboring MC through two hydrogen bonds: O15—H15*O*⋯O3^i^ and C32—H32⋯O3^i^ [symmetry code: (i) *x* − 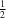, −*y* + 1, *z* + 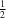]. The hydroxyl group (O21) of a 4-hy­droxy­benzoate anion also forms a hydrogen bond to a second MC *via* two hydrogen bonds: O21—H21*O*⋯O22^ii^ (a 4-hy­droxy­benzoate carboxyl­ate oxygen atom) and C46—H46⋯O9^ii^ [a phenolate oxygen atom of a shi^3−^ ligand; symmetry code: (ii) *x* − 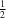, −*y* + 2, *z* − 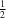]. Lastly, the hydroxyl group (O24) of a 4-hy­droxy­benzoate anion forms a hydrogen bond to a third MC *via* the hydrogen bond O24—H24*O*⋯O6^iii^ [a phenolate oxygen atom of a shi^3−^ ligand; symmetry code: (iii) *x* − 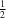, −*y* + 2, *z* + 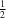]. Since each MC then forms reciprocal hydrogen bonds, each MC is hydrogen bonded to six neighboring MCs, forming a network of MCs. The hydrogen bonding between the neighboring MCs, between the MCs and the DMF mol­ecules, and pure van der Waals forces contribute to the overall packing of the mol­ecules.

## Database survey   

A survey of the Cambridge Structural Database (CSD version 5.41, update March 2020, Groom *et al.*, 2016 ▸) reveals that twenty-six *Ln*[12-MC_Mn(III)N(shi)_-4] complexes have been previously reported. Four of the metallacrowns contain both Dy^III^ and Na^+^ ions in the central cavity of the MC. The complexes have different bridging carboxyl­ate anions – acetate (OAc), benzoate (ben), 2-hy­droxy­benzoate (2-OHben), and tri­methyl­acetate (TMA): Dy(OAc)_4_Na[12-MC_Mn(III)N(shi)_-4](H_2_O)_4_·6DMF, **3** (TIWVIG; Azar *et al.*, 2014 ▸), Dy(ben)_4_Na[12-MC_Mn(III)N(shi)_-4](H_2_O)_4_·5DMF, **4** (HADFEA; Boron III *et al.*, 2016 ▸), Dy(2-OHben)_4_Na[12-MC_Mn(III)N(shi)_-4](DMF)(H_2_O)_3_·4DMF, **5** (HADFAW; Boron III *et al.*, 2016 ▸), and Dy(TMA)_4_Na[12-MC_Mn(III)N(shi)_-4](H_2_O)_2.59_(DMF)_1.41_·4DMF·0.59H_2_O, **6** (HADFOK; Boron III *et al.*, 2016 ▸).

In addition, three of the 12-MC-4 complexes contain both Dy^III^ and K^+^ in the central cavity with the bridging ligands acetate, benzoate, and 2-hy­droxy­benzoate: Dy(OAc)_4_K[12-MC_Mn(III)N(shi)_-4](DMF)_4_·DMF (TIWWUT; Azar *et al.*, 2014 ▸), Dy(ben)_4_K[12-MC_Mn(III)N(shi)_-4](H_2_O)_4_·4DMF·1.6H_2_O (HADFIE; Boron *et al.*, 2016 ▸), and Dy(2-OHben)_3.5_(OAc)_0.5_K[12-MC_Mn(III)N(shi)_-4](DMF)_1.5_(H_2_O)_3.5_·5DMF (HADDUO; Boron *et al.*, 2016 ▸).

Lastly, one dysprosium-manganese 12-MC-4 complex has an unbound tri­ethyl­ammonium as the counter-cation instead of an alkali metal cation and acetate as the bridging ligand:

[NH(C_2_H_5_)_3_]{Dy(OAc)_4_[12-MC_Mn(III)N(shi)_-4]} (QIBWUW; Qin *et al.*, 2017 ▸).

As complexes **1** and **2** contain a sodium cation, the discussion will be limited to the [12-MC_Mn(III)N(shi)_-4] complexes **3**–**6** that also capture a dysprosium and a sodium cation in the central cavity. The use of 3-hy­droxy­benzoate and 4-hy­droxy­benzoate does not significantly alter the overall MC framework as a structural comparison of complexes **1**–**6** reveals that the metrical parameters of the structures are similar (Table 6 ▸). These features were measured and calculated using the program *Mercury* (Macrae *et al.*, 2020 ▸) and in the same fashion as previously described (Azar *et al.*, 2014 ▸). For **1** and **2**, all metrical values fall within the range of **3**–**6**. In addition, **1** and **2** are domed in a similar fashion as **3**–**6** with the average distance of the ring Mn^III^ ions above their equatorial plane being 0.14 Å for both **1** and **2**, which is consistent with the values for **3**–**6**. Overall the mol­ecular structure of the six complexes are analogous with only differing bridging carboxyl­ate anions.

## Synthesis and crystallization   

**Materials**

Sodium 3-hy­droxy­benzoate (>99.0%) and sodium 4-hy­droxy­benzoate (>99.0%) were purchased from TCI America. Salicyl­hydroxamic acid (H_3_shi, 99%) and dysprosium(III) nitrate penta­hydrate (99.9%) were purchased from Alfa Aesar. Manganese(II) acetate tetra­hydrate (99+%) was purchased from Acros Organics. *N*,*N*-di­methyl­formamide (ACS grade) and methanol (ACS grade) were purchased from Pharmco–Aaper. *N*,*N*-di­methyl­acetamide (>99.5%) was purchased from VWR Chemicals BDH. All reagents were used as received and without further purification.

**Synthesis of Dy^III^Na(3-OHben)_4_[12-MC_Mn(III)N(shi)_-4](H_2_O)_4_·10DMA, 1.** Manganese(II) acetate tetra­hydrate (2 mmol, 0.4912 g) was dissolved in 8 mL of DMA, resulting in a clear orange solution. In a separate beaker, dysprosium(III) nitrate penta­hydrate (0.250 mmol, 0.1108 g) and salicyl­hydroxamic acid (2 mmol, 0.3070 g) were dissolved in 8 mL of DMA, resulting in a clear and colorless solution. In another beaker, sodium 3-hy­droxy­benzoate (4 mmol, 0.6413 g) was mixed in 8 mL of DMA, resulting in an opaque yellow mixture as not all of the reagent dissolved. Then the manganese(II) acetate solution was added to the Dy(NO_3_)_3_/H_3_shi solution, resulting in a dark-brown solution. Following, the sodium 3-hy­droxy­benzoate solution was added to the former solution and no color change was observed. The solution was stirred overnight and filtered the next day. A brown precipitate and clear and colorless solid were recovered and discarded. The filtrate was a dark-brown solution. Slow evaporation of the filtrate at room temperature afforded X-ray quality black/dark-brown block-shaped crystals after six days. The percentage yield was 44% based on dysprosium(III) nitrate penta­hydrate.

**Synthesis of Dy^III^Na(4-OHben)_4_[12-MC_Mn(III)N(shi)_-4](H_2_O)_4_·4DMF, 2.** Manganese(II) acetate tetra­hydrate (2 mmol, 0.4904 g) was dissolved in a solvent mixture of 5 mL of DMF and 5 mL of methanol, resulting in a clear orange solution. In a separate beaker, dysprosium(III) nitrate penta­hydrate (0.250 mmol, 0.1099 g), sodium 4-hy­droxy­benzoate (4 mmol, 0.6411 g), and salicyl­hydroxamic acid (2 mmol, 0.3072 g) were mixed in a solvent mixture of 5 mL of DMF and 5 mL of methanol, and the resulting mixture had an opaque white color as not all of the reagents had dissolved. Then the manganese(II) acetate solution was added to the latter mixture, resulting in an opaque green solution. The solution was stirred overnight and filtered the next day. A green precipitate was recovered and discarded. The filtrate was a dark green–brown solution. Slow evaporation of the filtrate at room temperature afforded X-ray quality black/dark-brown block-shaped crystals after three weeks. The percentage yield was 56% based on dysprosium(III) nitrate penta­hydrate.

## Refinement   

For **1**, whole mol­ecule disorder is observed for the main mol­ecule, excluding only the Dy and Na ions. Equivalent disordered organic moieties were restrained to have similar geometries (SAME command of *SHELXL*), and *U^ij^* components of ADPs for all disordered atoms closer to each other than 2.0 Å were restrained to be similar (SIMU command of *SHELXL*). Subject to these conditions, the occupancy ratio refined to 0.8018 (14):0.1982 (14). Three DMA mol­ecules were refined as disordered. The two DMA mol­ecules associated with N2 and N3 are in general positions by an approximate 180° rotation. The third DMA mol­ecule associated with N4 is disordered by an exact 180° rotation from a twofold axis that bis­ects it as well as by additional general disorder. All DMA moieties were restrained to have similar geometries (SAME command of *SHELXL*). All N—CH_3_ bond lengths were restrained to be similar in length and all 1,3 distances of the C—N—CH_3_ angles were also restrained to be similar to each other. *U^ij^* components of ADPs for all DMA atoms closer to each other than 2.0 Å were restrained to be similar, and the atoms of the fourfold-disordered mol­ecule were restrained to be close to isotropic. The lowest occupancy DMA mol­ecule (the minor component disordered by twofold symmetry) was restrained to be close to planar. Subject to these conditions the occupancy ratios of the DMA mol­ecules associated with N2, N3, and N4 refined to 0.496 (8):0.504 (8), 0.608 (9):0.392 (9), and 2×0.275 (7):2×0.225 (7), respectively. Initially alcohol hydrogen atoms were allowed to rotate about their respective oxygen atoms, and water hydrogen-atom positions were refined while a damping factor was applied, and O—H and H⋯H distances were restrained to 0.84 (2) and 1.36 (2) Å, respectively. Some water hydrogen-atom positions were further restrained based on hydrogen-bonding considerations. In the final refinement cycles these hydrogen atoms were set to ride on their carrier oxygen atoms and the damping factor was removed. Additional crystal data, data collection, and structure refinement details are summarized in Table 7 ▸.

For **2** the crystal under investigation was found to be a non-merohedric twin. The orientation matrices for the two components were identified using the program *CELL_NOW* (Sheldrick, 2008*b* ▸), with the two components being related by a 90° rotation around the real *a* axis. The two components were integrated using *SAINT* (Bruker, 2018 ▸) and corrected for absorption using *TWINABS* (Sheldrick, 2012 ▸). The twin matrix obtained by the integration program was (1 0 0 0 0 1 0 − 1 0).

The structure was solved by direct methods with only the non-overlapping reflections of component 1. The structure was refined using all reflections of component 1 (including overlaps), resulting in a minor-component fraction of 0.0818 (8). The *R*_int_ value given is for all reflections and is based on agreement between observed single and composite intensities and those calculated from refined unique intensities and twin fractions (*TWINABS;* Sheldrick 2012 ▸). Sections of the metallacrown are disordered including the Dy ion, Mn1, two of the 4-hy­droxy­benzoate ligands bound to Mn1 and Mn2, the salicyl­hydroximate ligand that connects Mn1 and Mn4, and portions of the remaining three salicyl­hydroximate ligands. The major moiety 4-hy­droxy­benzoate anion geometry was restrained to be similar to that of a non-disordered 4-hy­droxy­benzoate. The geometry of the entire minor moiety was restrained to be similar to that of the major moiety. Some sections of the minor disordered salicyl­hydroximate ligands were restrained to be planar. Pairs of close to overlapping equivalent atoms of the major and minor moieties were constrained to have identical ADPs (C1 and C1*B*, N2 and N2*B*, O4 and O4*B*, O7 and O7*B*, C22 and C22*B*, Dy1 and Dy1*B*). Two solvate DMF mol­ecules are disordered over different orientations. The major and minor disordered moieties were each restrained to have similar geometries. *U^ij^* components of ADPs for all disordered atoms closer to each other than 2.0 Å were restrained to be similar. Subject to these conditions the occupancy ratio for the main mol­ecule disorder refined to 0.849 (9):0.151 (9). The disorder of the two DMF moieties refined to 0.64 (3):0.36 (3) for the DMF associated with N5 and to 0.51 (2):0.49 (2) for the DMF mol­ecule associated with N8. Water hydrogen atom positions were refined and O—H and H⋯H distances were restrained to 0.84 (2) and 1.36 (2) Å, respectively. Some water hydrogen-atom positions were further restrained based on hydrogen-bonding considerations and were restrained to be at least 3.10 (2) Å from the sodium ion. Additional crystal data, data collection, and structure refinement details are summarized in Table 7 ▸.

## Supplementary Material

Crystal structure: contains datablock(s) 2, 1, global. DOI: 10.1107/S2056989020008853/pk2637sup1.cif

Structure factors: contains datablock(s) 1. DOI: 10.1107/S2056989020008853/pk26371sup2.hkl

Structure factors: contains datablock(s) 2. DOI: 10.1107/S2056989020008853/pk26372sup3.hkl

CCDC references: 2013185, 2013184

Additional supporting information:  crystallographic information; 3D view; checkCIF report

## Figures and Tables

**Figure 2:**
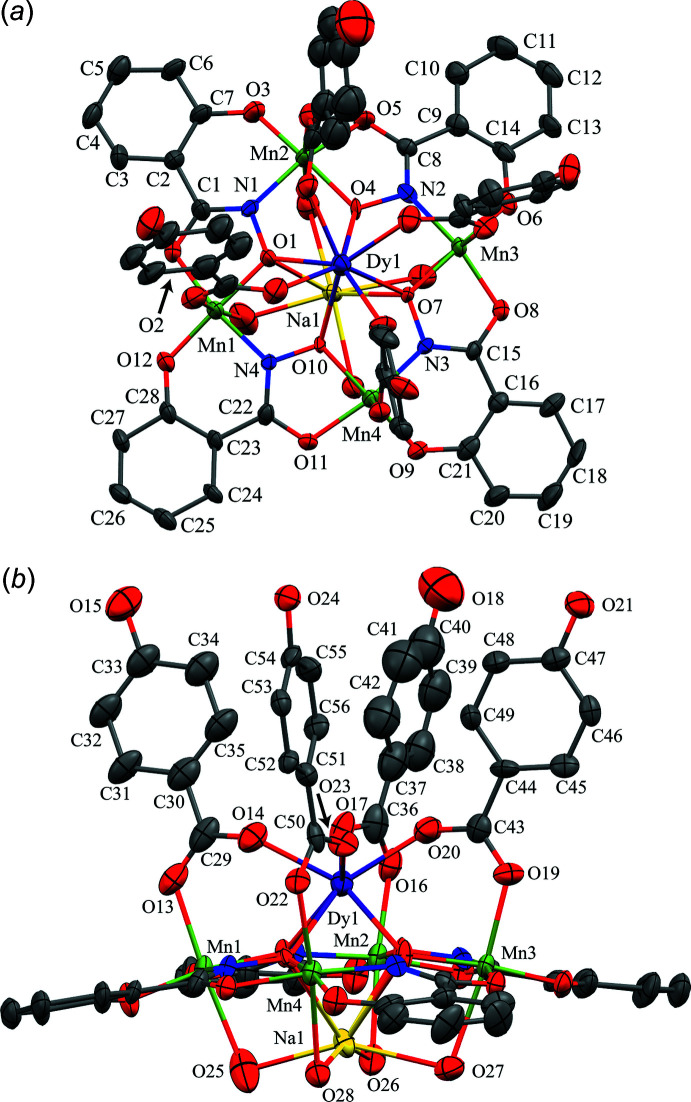
The single-crystal X-ray structure of Dy^III^Na(4-OHben)_4_[12-MC_Mn(III)N(shi)_-4](H_2_O)_4_·4DMF, **2**, (*a*) top view with only the metal atoms and shi^3−^ ligands labeled for clarity and (*b*) side view with only the metal atoms and axial ligands labeled for clarity. The displacement ellipsoids are drawn at the 50% probability level. For clarity, hydrogen atoms, solvent mol­ecules, and disorder have been omitted. See Fig. 1 ▸ for additional display details.

**Table 1:** Average bond length (Å) and bond-valence-sum (BVS) values (v.u.) used to support assigned oxidation states of the dysprosium and manganese ions of **1** and **2**

	Avg. bond length	BVS value	Assigned oxidation state
**1**			
Dy1	2.339	3.32	3+
Mn1	2.053	3.02	3+
			
**2**			
Dy1	2.357	3.17	3+
Mn1	2.038	3.13	3+
Mn2	2.03	3.11	3+
Mn3	2.031	3.22	3+
Mn4	2.055	3.05	3+

**Table 2:** Continuous Shapes Measures (CShM) values for the geometry about the six-coordinate ring Mn^III^ ions in **1** and **2**

Shape	Hexagon (*D_6h_*)	Penta­gonal pyramid (*C_5v_*)	Octa­hedron (*O_h_*)	Trigonal prism (*D_3h_*)	Johnson penta­gonal pyramid (J2; *C_5v_*)
**1**					
Mn1	30.226	27.832	1.147	17.090	30.691
					
**2**					
Mn1	30.178	27.324	1.126	16.539	30.302
Mn2	29.625	27.265	1.115	16.232	29.492
Mn3	30.366	28.015	1.145	16.300	30.249
Mn4	29.517	26.990	1.434	15.615	29.813

**Table 3:** Continuous Shapes Measures (CShM) values for the geometry about the eight-coordinate central Dy^III^ and Na^+^ ions in **1** and **2**

Shape	**1**		**2**	
	Dy^III^	Na^+^	Dy^III^	Na^+^
Octa­gon (*D_8h_*)	31.416	30.418	32.709	29.627
Heptagonal pyramid (*C_7v_*)	23.704	25.842	23.084	25.952
Hexagonal bipyramid (*D_6h_*)	17.239	13.946	16.431	14.078
Cube (*O_h_*)	9.655	6.064	9.477	6.784
Square anti­prism (*D_4d_*)	0.550	3.063	0.818	3.657
Triangular dodeca­hedron (*D_2d_*)	2.708	3.797	2.517	4.233
Johnson – gyrobifastigium (J26; *D_2d_*)	17.567	16.821	16.670	16.504
Johnson – elongated triangular bipyramid (J14; *D_3h_*)	30.145	29.438	29.907	29.093
Johnson – biaugmented trigonal prism (J50; *C_2v_*)	2.927	4.700	3.128	5.084
Biaugmented trigonal prism (*C_2v_*)	1.995	3.002	2.160	3.196
Johnson – snub disphenoid (J84; *D_2d_*)	5.823	7.668	5.580	7.860
Triakis tetra­hedron (*T_d_*)	10.516	6.959	10.266	7.625
Elongated trigonal bipyramid (*D_3h_*)	25.542	25.071	25.294	24.594

**Table 4:** Hydrogen-bond geometry (Å, °) for **1** [Chem scheme1]

*D*—H⋯*A*	*D*—H	H⋯*A*	*D*⋯*A*	*D*—H⋯*A*
O6—H6*A*1⋯O9^i^	0.84	1.82	2.542 (13)	143
O7—H7*A*⋯O8	0.82	1.85	2.653 (9)	165
O7—H7*B*⋯O8^ii^	0.82	2.05	2.785 (9)	151
C17—H17*B*⋯O6^iii^	0.98	2.53	3.348 (19)	141

Symmetry codes: (i) 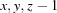; (ii) 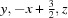; (iii) 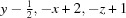.

**Table 5:** Hydrogen-bond geometry (Å, °) for **2** [Chem scheme1]

*D*—H⋯*A*	*D*—H	H⋯*A*	*D*⋯*A*	*D*—H⋯*A*
O25—H25*C*⋯O29	0.92	2.00	2.74 (3)	137
O25—H25*D*⋯O12	0.87	2.41	3.06 (2)	132
O26—H26*C*⋯O30	0.85 (4)	2.04 (9)	2.74 (2)	138 (10)
O26—H26*D*⋯O29	0.84 (4)	2.03 (11)	2.70 (4)	136 (11)
O27—H27*C*⋯O30	0.87 (4)	2.12 (14)	2.730 (19)	127 (14)
O27—H27*D*⋯O31	0.87 (4)	2.09 (7)	2.798 (18)	138 (6)
O28—H28*C*⋯O31	0.88 (4)	2.07 (10)	2.776 (17)	137 (10)
O28—H28*D*⋯O32	0.88 (4)	1.94 (7)	2.68 (3)	142 (6)
C32—H32⋯O3^i^	0.95	2.66	3.35 (2)	131
O15—H15*O*⋯O3^i^	0.84	1.93	2.77 (2)	175
C46—H46⋯O9^ii^	0.95	2.24	3.168 (15)	165
O21—H21*O*⋯O22^ii^	0.84	2.01	2.794 (16)	155
O24—H24*O*⋯O6^iii^	0.84	2.02	2.815 (16)	158

Symmetry codes: (i) 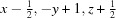; (ii) 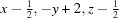; (iii) 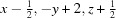.

**Table 6:** Structural comparison of **1** and **2** with other Dy^III^Na(*X*)_4_[12-MC_Mn(III)N(shi)_-4] complexes (Å)

Compound	Dy^III^ crystal radius	MC crystal radius	Avg. cross-cavity Mn^III^⋯Mn^III^ distance	Avg. cross-cavity O_ox_⋯O_ox_ distance	Dy^III^—O_ox_MP distance	Dy^III^—O_car_MP distance	Avg. distance of Mn to equatorial atom MP
**1**	1.04	0.56	6.53	3.72	1.55	1.06	0.14
**2**	1.06	0.54	6.49	3.69	1.59	1.08	0.14
**3**	1.06	0.55	6.52	3.71	1.59	1.03	0.17
**4**	1.05	0.54	6.51	3.69	1.58	1.05	0.14
**5**	1.03	0.54	6.47	3.68	1.51	1.15	0.06
**6**	1.06	0.56	6.51	3.73	1.58	1.05	0.17

**Table 7:** Experimental details

	**1**	**2**
Crystal data		
Chemical formula	[DyMn_4_Na(C_7_H_5_O_3_)_4_(C_7_H_4_NO_2_)_4_(H_2_O)_4_]·10C_4_H_9_NO	[DyMn_4_Na(C_7_H_5_O_3_)_4_(C_7_H_4_NO_2_)_4_(H_2_O)_4_]·4C_3_H_7_NO
*M*_r_	2497.41	1918.58
Crystal system, space group	Tetragonal, *P*4/*n*	Monoclinic, *P* *n*
Temperature (K)	150	150
*a*, *b*, *c* (Å)	19.9869 (9), 19.9869 (9), 13.9570 (11)	14.3622 (11), 16.5258 (11), 16.8246 (12)
α, β, γ (°)	90, 90, 90	90, 92.347 (3), 90
*V* (Å^3^)	5575.5 (7)	3989.9 (5)
*Z*	2	2
Radiation type	Mo *K*α	Mo *K*α
μ (mm^−1^)	1.19	1.64
Crystal size (mm)	0.25 × 0.23 × 0.15	0.30 × 0.20 × 0.19

Data collection		
Diffractometer	Bruker AXS D8 Quest CMOS	Bruker AXS D8 Quest CMOS
Absorption correction	Multi-scan (*SADABS*; Krause *et al.*, 2015 ▸)	Multi-scan (*TWINABS*; Sheldrick, 2012 ▸)
*T*_min_, *T*_max_	0.024, 0.055	0.053, 0.109
No. of measured, independent and observed [*I* > 2σ(*I*)] reflections	58638, 7967, 6605	40158, 40158, 29137
*R*_int_	0.042	0.084
(sin θ/λ)_max_ (Å^−1^)	0.714	0.667

Refinement		
*R*[*F* ^2^ > 2σ(*F* ^2^)], *wR*(*F* ^2^), *S*	0.051, 0.151, 1.04	0.073, 0.219, 1.07
No. of reflections	7967	40158
No. of parameters	761	1433
No. of restraints	1550	1908
H-atom treatment	H-atom parameters constrained	H atoms treated by a mixture of independent and constrained refinement
Δρ_max_, Δρ_min_ (e Å^−3^)	2.49, −0.91	1.86, −1.83
Absolute structure	–	Flack *x* determined using 5372 quotients [(*I* ^+^)−(*I* ^−^)]/[(*I* ^+^)+(*I* ^−^)] (Parsons *et al.*, 2013 ▸)
Absolute structure parameter	–	−0.025 (7)

Computer programs: *APEX3* and *SAINT* (Bruker, 2018 ▸), *SHELXS97* (Sheldrick, 2008*a* ▸), *SHELXL2018/3* (Sheldrick, 2015 ▸), *shelXle* (Hübschle *et al.*, 2011 ▸), *Mercury* (Macrae *et al.*, 2020 ▸) and *publCIF* (Westrip, 2010 ▸).

## supplementary crystallographic information

### Tetra-µ-aqua-tetrakis{2-[azanidylene(oxido)methyl]phenolato}tetrakis(µ2-3-hydroxybenzoato)dysprosium(III)tetramanganese(III)sodium(I) N,N-dimethylformamide tetrasolvate (2) Crystal data

**Table:**

[DyMn_4_Na(C_7_H_5_O_3_)_4_(C_7_H_4_NO_2_)_4_(H_2_O)_4_]·4C_3_H_7_NO	*F*(000) = 1938
*M**_r_* = 1918.58	*D*_x_ = 1.597 Mg m^−^^3^
Monoclinic, *P**n*	Mo *K*α radiation, λ = 0.71073 Å
*a* = 14.3622 (11) Å	Cell parameters from 9804 reflections
*b* = 16.5258 (11) Å	θ = 2.8–34.6°
*c* = 16.8246 (12) Å	µ = 1.64 mm^−^^1^
β = 92.347 (3)°	*T* = 150 K
*V* = 3989.9 (5) Å^3^	Prism, green
*Z* = 2	0.30 × 0.20 × 0.19 mm

### Tetra-µ-aqua-tetrakis{2-[azanidylene(oxido)methyl]phenolato}tetrakis(µ2-3-hydroxybenzoato)dysprosium(III)tetramanganese(III)sodium(I) N,N-dimethylformamide tetrasolvate (2) Data collection

**Table:**

Bruker AXS D8 Quest CMOS diffractometer	40158 independent reflections
Radiation source: sealed tube X-ray source	29137 reflections with *I* > 2σ(*I*)
Triumph curved graphite crystal monochromator	*R*_int_ = 0.084
ω and phi scans	θ_max_ = 28.3°, θ_min_ = 2.9°
Absorption correction: multi-scan (*TWINABS*; Sheldrick, 2012)	*h* = −19→19
*T*_min_ = 0.053, *T*_max_ = 0.109	*k* = −22→22
40158 measured reflections	*l* = −22→22

### Tetra-µ-aqua-tetrakis{2-[azanidylene(oxido)methyl]phenolato}tetrakis(µ2-3-hydroxybenzoato)dysprosium(III)tetramanganese(III)sodium(I) N,N-dimethylformamide tetrasolvate (2) Refinement

**Table:**

Refinement on *F*^2^	Secondary atom site location: difference Fourier map
Least-squares matrix: full	Hydrogen site location: mixed
*R*[*F*^2^ > 2σ(*F*^2^)] = 0.073	H atoms treated by a mixture of independent and constrained refinement
*wR*(*F*^2^) = 0.219	*w* = 1/[σ^2^(*F*_o_^2^) + (0.1018*P*)^2^ + 15.3803*P*] where *P* = (*F*_o_^2^ + 2*F*_c_^2^)/3
*S* = 1.07	(Δ/σ)_max_ < 0.001
40158 reflections	Δρ_max_ = 1.86 e Å^−^^3^
1433 parameters	Δρ_min_ = −1.83 e Å^−^^3^
1908 restraints	Absolute structure: Flack *x* determined using 5372 quotients [(*I*^+^)-(*I*^-^)]/[(*I*^+^)+(*I*^-^)] (Parsons *et al.*, 2013)
Primary atom site location: structure-invariant direct methods	Absolute structure parameter: −0.025 (7)

### Tetra-µ-aqua-tetrakis{2-[azanidylene(oxido)methyl]phenolato}tetrakis(µ2-3-hydroxybenzoato)dysprosium(III)tetramanganese(III)sodium(I) N,N-dimethylformamide tetrasolvate (2) Special details

**Table:**

Geometry. All esds (except the esd in the dihedral angle between two l.s. planes) are estimated using the full covariance matrix. The cell esds are taken into account individually in the estimation of esds in distances, angles and torsion angles; correlations between esds in cell parameters are only used when they are defined by crystal symmetry. An approximate (isotropic) treatment of cell esds is used for estimating esds involving l.s. planes.
Refinement. The crystal under investigation was found to be non-merohedrally twinned. The orientation matrices for the two components were identified using the program Cell_Now, with the two components being related by a 90 degree rotation around the real a-axis. The two components were integrated using Saint and corrected for absorption using twinabs, resulting in the following statistics:20647 data (5279 unique) involve domain 1 only, mean I/sigma 37.1 19807 data (5143 unique) involve domain 2 only, mean I/sigma 9.5 63719 data (23583 unique) involve 2 domains, mean I/sigma 23.2 172 data (172 unique) involve 3 domains, mean I/sigma 29.5 The exact twin matrix identified by the integration program was found to be: 0.99975 -0.00605 -0.00410 0.04844 0.02512 0.98410 -0.05702 -1.01529 0.02202The structure was solved using direct methods with only the non-overlapping reflections of component 1. The structure was refined using the hklf 5 routine with all reflections of component 1 (including the overlapping ones), resulting in a BASF value of 0.0818 (8).The Rint value given is for all reflections and is based on agreement between observed single and composite intensities and those calculated from refined unique intensities and twin fractions (TWINABS (Sheldrick, 2012)).Large sections of the main molecule are disordered, including two of the 4-hydroxybenzoate ligands, the Dy atom, manganese atom Mn1, one of the salicylhydroximate ligands, and part of another. The main difference between the major and minor moieties is the coordination mode of one of the 4-hydroxybenzoate anions. In the major moiety, all 4-hydroxybenzoate anions are coordinated to the Dy atom. In the minor moiety, O17B is detached from the Dy atom. Major moiety 4-hydroxybenzoate anion geometries were restrained to be similar to that of a not disordered 4-hydroxybenzoate. The geometry of the whole minor moiety was restrained to be similar to that of the major moiety. Some sections of the minor disordered salicylhydroximate ligands were restrained to be planar. Pairs of close to overlapping equivalent atoms of the major and minor moieties were constrained to have identical ADPs (C1 and C1B, N2 and N2B, O4 and O4B, O7 and O7B, C22 and C22B, Dy1 and Dy1B). Two solvate DMF molecules are disordered over different orientations. The major and minor disordered moieties were each restrained to have similar geometries. Uij components of ADPs for all disordered atoms closer to each other than 1.7 Angstrom were restrained to be similar. Subject to these conditions the occupancy ratio for the main molecule disorder refined to 0.849 (9) to 0.151 (9). The disorder of the two DMF moieties refined to 0.64 (3) to 0.36 (3) and 0.51 (2) to 0.49 (2). Water H atom positions were refined and O-H and H···H distances were restrained to 0.84 (2) and 1.36 (2) Angstrom, respectively. Some water H atom positions were further restrained based on hydrogen bonding considerations, and were restrained to be at least 3.1 Angstrom from the next sodium ions.

### Tetra-µ-aqua-tetrakis{2-[azanidylene(oxido)methyl]phenolato}tetrakis(µ2-3-hydroxybenzoato)dysprosium(III)tetramanganese(III)sodium(I) N,N-dimethylformamide tetrasolvate (2) Fractional atomic coordinates and isotropic or equivalent isotropic displacement parameters (Å^2^)

**Table:**

	*x*	*y*	*z*	*U*_iso_*/*U*_eq_	Occ. (<1)
Mn2	0.2835 (2)	0.54730 (13)	−0.06110 (14)	0.0270 (5)	
Mn3	0.31469 (19)	0.79391 (13)	−0.18204 (14)	0.0229 (5)	
Mn4	0.3714 (2)	0.91325 (13)	0.06191 (14)	0.0241 (5)	
Na1	0.4472 (5)	0.7150 (4)	−0.0061 (4)	0.0308 (13)	
O3	0.2981 (10)	0.4372 (7)	−0.0438 (7)	0.045 (3)	
O5	0.2923 (9)	0.5445 (6)	−0.1758 (6)	0.035 (3)	
O6	0.3280 (8)	0.7720 (7)	−0.2890 (6)	0.033 (2)	
O8	0.3581 (8)	0.9052 (6)	−0.1857 (6)	0.028 (2)	
O9	0.4259 (8)	1.0102 (6)	0.0385 (6)	0.032 (2)	
O25	0.4984 (13)	0.6609 (10)	0.1204 (8)	0.073 (5)	
H25C	0.521690	0.610050	0.131164	0.110*	
H25D	0.507005	0.679723	0.168435	0.110*	
O26	0.4477 (10)	0.5722 (8)	−0.0585 (8)	0.047 (3)	
H26C	0.458 (16)	0.560 (4)	−0.106 (4)	0.070*	
H26D	0.461 (15)	0.531 (3)	−0.031 (4)	0.070*	
O27	0.4763 (9)	0.7604 (8)	−0.1437 (8)	0.041 (3)	
H27C	0.473 (14)	0.732 (6)	−0.187 (3)	0.062*	
H27D	0.514 (12)	0.799 (8)	−0.157 (4)	0.062*	
O28	0.5181 (8)	0.8430 (7)	0.0402 (7)	0.037 (3)	
H28C	0.539 (12)	0.882 (6)	0.010 (4)	0.055*	
H28D	0.555 (11)	0.849 (5)	0.082 (6)	0.055*	
N1	0.2942 (9)	0.5681 (7)	0.0536 (7)	0.025 (3)	
N3	0.3442 (8)	0.8943 (7)	−0.0522 (7)	0.021 (2)	
C2	0.3108 (13)	0.4324 (9)	0.1012 (10)	0.036 (4)	
C3	0.3158 (14)	0.3808 (11)	0.1678 (11)	0.045 (4)	
H3	0.321500	0.404634	0.219165	0.054*	
C4	0.3131 (14)	0.3007 (10)	0.1630 (13)	0.048 (5)	
H4	0.318659	0.268751	0.209894	0.057*	
C5	0.3019 (15)	0.2639 (11)	0.0889 (13)	0.050 (5)	
H5	0.298348	0.206621	0.085189	0.060*	
C6	0.2960 (13)	0.3110 (9)	0.0199 (11)	0.041 (4)	
H6	0.288418	0.285858	−0.030755	0.049*	
C7	0.3011 (13)	0.3945 (9)	0.0258 (10)	0.036 (4)	
C8	0.2950 (10)	0.6170 (9)	−0.2053 (9)	0.025 (3)	
C9	0.3028 (11)	0.6270 (10)	−0.2927 (9)	0.031 (3)	
C10	0.2919 (13)	0.5583 (12)	−0.3392 (10)	0.041 (4)	
H10	0.283475	0.507565	−0.313951	0.050*	
C11	0.2931 (16)	0.5613 (14)	−0.4217 (11)	0.057 (5)	
H11	0.289898	0.512984	−0.452367	0.068*	
C12	0.2989 (15)	0.6353 (14)	−0.4581 (11)	0.055 (5)	
H12	0.294288	0.638969	−0.514457	0.067*	
C13	0.3114 (14)	0.7048 (12)	−0.4129 (9)	0.043 (4)	
H13	0.319022	0.755453	−0.438531	0.052*	
C14	0.3130 (12)	0.7010 (11)	−0.3293 (9)	0.036 (4)	
C15	0.3678 (10)	0.9356 (8)	−0.1154 (9)	0.024 (3)	
C16	0.4047 (11)	1.0180 (9)	−0.1058 (10)	0.029 (3)	
C17	0.4135 (12)	1.0652 (10)	−0.1742 (12)	0.040 (4)	
H17	0.396735	1.043455	−0.225069	0.048*	
C18	0.4466 (15)	1.1437 (11)	−0.1674 (12)	0.051 (5)	
H18	0.453749	1.175257	−0.213923	0.062*	
C19	0.4683 (17)	1.1751 (12)	−0.0974 (13)	0.058 (6)	
H19	0.489161	1.229604	−0.094324	0.070*	
C20	0.4617 (13)	1.1311 (10)	−0.0281 (11)	0.042 (4)	
H20	0.479156	1.155589	0.021346	0.051*	
C21	0.4290 (11)	1.0496 (9)	−0.0294 (10)	0.033 (3)	
Dy1	0.20229 (13)	0.74838 (9)	0.01112 (12)	0.0316 (4)	0.849 (9)
O1	0.3067 (10)	0.6491 (7)	0.0745 (7)	0.026 (3)	0.849 (9)
O2	0.3466 (11)	0.5481 (7)	0.1814 (8)	0.035 (3)	0.849 (9)
O4	0.2849 (9)	0.6613 (8)	−0.0795 (7)	0.022 (3)	0.849 (9)
N2	0.2911 (19)	0.6821 (12)	−0.1615 (10)	0.025 (3)	0.849 (9)
C1	0.3227 (16)	0.5211 (11)	0.1115 (10)	0.035 (3)	0.849 (9)
N4	0.3568 (11)	0.7807 (8)	0.1614 (8)	0.020 (3)	0.849 (9)
O7	0.3147 (10)	0.8160 (9)	−0.0710 (8)	0.024 (3)	0.849 (9)
O10	0.3366 (8)	0.8047 (7)	0.0836 (7)	0.018 (2)	0.849 (9)
O11	0.4079 (13)	0.9099 (9)	0.1749 (7)	0.025 (4)	0.849 (9)
C22	0.3931 (13)	0.8404 (10)	0.2061 (10)	0.026 (3)	0.849 (9)
C23	0.4176 (12)	0.8262 (10)	0.2907 (9)	0.025 (3)	0.849 (9)
C24	0.4365 (16)	0.8932 (12)	0.3390 (10)	0.029 (4)	0.849 (9)
H24	0.432946	0.946283	0.317536	0.035*	0.849 (9)
C25	0.4608 (18)	0.8812 (13)	0.4194 (11)	0.038 (4)	0.849 (9)
H25	0.472752	0.926892	0.452556	0.045*	0.849 (9)
C26	0.4678 (16)	0.8065 (13)	0.4509 (12)	0.039 (4)	0.849 (9)
H26	0.485772	0.800058	0.505469	0.047*	0.849 (9)
C27	0.4487 (15)	0.7389 (12)	0.4041 (10)	0.034 (4)	0.849 (9)
H27	0.453598	0.686491	0.427100	0.041*	0.849 (9)
C28	0.4225 (14)	0.7469 (10)	0.3235 (9)	0.031 (3)	0.849 (9)
O12	0.4088 (12)	0.6786 (8)	0.2813 (7)	0.037 (3)	0.849 (9)
Mn1	0.3482 (3)	0.6653 (2)	0.1823 (2)	0.0277 (9)	0.849 (9)
O13	0.2110 (11)	0.6665 (9)	0.2286 (9)	0.043 (3)	0.849 (9)
O14	0.1442 (12)	0.7440 (9)	0.1332 (9)	0.044 (3)	0.849 (9)
C29	0.1409 (15)	0.6966 (12)	0.1932 (12)	0.044 (4)	0.849 (9)
C30	0.0464 (14)	0.6781 (13)	0.2227 (12)	0.051 (4)	0.849 (9)
C31	0.0398 (15)	0.6421 (12)	0.2982 (12)	0.049 (4)	0.849 (9)
H31	0.094344	0.628183	0.329067	0.058*	0.849 (9)
C32	−0.0473 (14)	0.6277 (13)	0.3264 (13)	0.050 (4)	0.849 (9)
H32	−0.052485	0.604136	0.377566	0.060*	0.849 (9)
C33	−0.1284 (15)	0.647 (2)	0.2815 (14)	0.052 (5)	0.849 (9)
C34	−0.1207 (16)	0.6812 (15)	0.2059 (14)	0.058 (4)	0.849 (9)
H34	−0.175024	0.693090	0.173960	0.069*	0.849 (9)
C35	−0.0328 (15)	0.6978 (15)	0.1778 (14)	0.054 (4)	0.849 (9)
H35	−0.027476	0.722949	0.127404	0.065*	0.849 (9)
O15	−0.2158 (12)	0.6408 (10)	0.3106 (11)	0.063 (5)	0.849 (9)
H15O	−0.213172	0.614349	0.353246	0.095*	0.849 (9)
Dy1B	0.1734 (8)	0.7497 (7)	0.0004 (8)	0.0316 (4)	0.151 (9)
O1B	0.272 (4)	0.650 (2)	0.075 (2)	0.022 (9)	0.151 (9)
O2B	0.299 (6)	0.553 (2)	0.187 (3)	0.030 (6)	0.151 (9)
O4B	0.262 (6)	0.655 (4)	−0.079 (4)	0.022 (3)	0.151 (9)
N2B	0.282 (11)	0.669 (7)	−0.160 (5)	0.025 (3)	0.151 (9)
C1B	0.300 (4)	0.522 (2)	0.116 (3)	0.035 (3)	0.151 (9)
N4B	0.315 (5)	0.783 (2)	0.161 (3)	0.022 (5)	0.151 (9)
O7B	0.297 (6)	0.807 (5)	−0.076 (4)	0.024 (3)	0.151 (9)
O10B	0.306 (4)	0.804 (3)	0.080 (3)	0.020 (7)	0.151 (9)
O11B	0.388 (10)	0.908 (5)	0.175 (2)	0.026 (7)	0.151 (9)
C22B	0.363 (7)	0.838 (3)	0.200 (3)	0.026 (3)	0.151 (9)
C23B	0.390 (5)	0.827 (3)	0.286 (3)	0.028 (5)	0.151 (9)
C24B	0.422 (8)	0.895 (3)	0.327 (4)	0.032 (6)	0.151 (9)
H24B	0.425825	0.945595	0.299777	0.038*	0.151 (9)
C25B	0.449 (9)	0.890 (4)	0.407 (4)	0.035 (6)	0.151 (9)
H25B	0.470631	0.936208	0.434753	0.042*	0.151 (9)
C26B	0.444 (8)	0.816 (5)	0.446 (3)	0.036 (6)	0.151 (9)
H26B	0.461723	0.811941	0.500680	0.043*	0.151 (9)
C27B	0.412 (6)	0.748 (4)	0.405 (3)	0.034 (6)	0.151 (9)
H27B	0.408009	0.697062	0.431630	0.041*	0.151 (9)
C28B	0.385 (4)	0.753 (3)	0.325 (3)	0.031 (5)	0.151 (9)
O12B	0.355 (5)	0.684 (3)	0.288 (2)	0.027 (6)	0.151 (9)
Mn1B	0.3022 (17)	0.6696 (11)	0.1860 (10)	0.022 (3)	0.151 (9)
O13B	0.160 (3)	0.676 (4)	0.221 (3)	0.046 (6)	0.151 (9)
O14B	0.106 (4)	0.750 (4)	0.117 (3)	0.047 (8)	0.151 (9)
C29B	0.093 (3)	0.704 (6)	0.178 (4)	0.047 (5)	0.151 (9)
C30B	−0.005 (4)	0.677 (7)	0.185 (5)	0.052 (6)	0.151 (9)
C31B	−0.036 (4)	0.647 (7)	0.257 (5)	0.052 (5)	0.151 (9)
H31B	0.006684	0.630446	0.298238	0.063*	0.151 (9)
C32B	−0.132 (5)	0.641 (14)	0.265 (6)	0.053 (6)	0.151 (9)
H32B	−0.154361	0.629196	0.316536	0.064*	0.151 (9)
C33B	−0.196 (4)	0.651 (7)	0.202 (5)	0.055 (7)	0.151 (9)
C34B	−0.164 (4)	0.692 (8)	0.136 (5)	0.055 (7)	0.151 (9)
H34B	−0.207699	0.707294	0.094201	0.066*	0.151 (9)
C35B	−0.071 (4)	0.710 (7)	0.130 (6)	0.054 (7)	0.151 (9)
H35B	−0.051631	0.744938	0.088435	0.064*	0.151 (9)
O15B	−0.290 (4)	0.644 (6)	0.211 (6)	0.068 (15)	0.151 (9)
H15B	−0.318293	0.677120	0.181176	0.103*	0.151 (9)
O16	0.1346 (10)	0.5323 (9)	−0.0740 (8)	0.041 (3)	0.849 (9)
O17	0.1018 (12)	0.6375 (8)	0.0031 (8)	0.045 (3)	0.849 (9)
C36	0.0793 (16)	0.5813 (15)	−0.0465 (14)	0.049 (4)	0.849 (9)
C37	−0.0219 (16)	0.5729 (15)	−0.0671 (16)	0.063 (4)	0.849 (9)
C38	−0.0536 (19)	0.5039 (17)	−0.108 (2)	0.069 (5)	0.849 (9)
H38	−0.012944	0.461690	−0.122684	0.083*	0.849 (9)
C39	−0.1498 (19)	0.5016 (18)	−0.125 (2)	0.078 (5)	0.849 (9)
H39	−0.175599	0.455806	−0.152122	0.094*	0.849 (9)
C40	−0.2081 (19)	0.5630 (18)	−0.104 (2)	0.086 (6)	0.849 (9)
C41	−0.172 (2)	0.6317 (18)	−0.067 (2)	0.089 (6)	0.849 (9)
H41	−0.212367	0.676146	−0.057268	0.106*	0.849 (9)
C42	−0.0804 (19)	0.6362 (17)	−0.0426 (19)	0.079 (5)	0.849 (9)
H42	−0.056917	0.679874	−0.011034	0.095*	0.849 (9)
O18	−0.3015 (16)	0.561 (2)	−0.121 (2)	0.122 (8)	0.849 (9)
H18O	−0.311444	0.564554	−0.170632	0.182*	0.849 (9)
O16B	0.135 (3)	0.581 (5)	−0.074 (4)	0.043 (6)	0.151 (9)
O17B	0.054 (6)	0.606 (6)	0.035 (4)	0.065 (13)	0.151 (9)
C36B	0.060 (3)	0.584 (8)	−0.038 (4)	0.050 (6)	0.151 (9)
C37B	−0.029 (3)	0.571 (6)	−0.085 (5)	0.065 (6)	0.151 (9)
C38B	−0.051 (3)	0.492 (6)	−0.107 (9)	0.070 (6)	0.151 (9)
H38B	−0.004884	0.451473	−0.104425	0.084*	0.151 (9)
C39B	−0.142 (4)	0.473 (5)	−0.132 (10)	0.078 (7)	0.151 (9)
H39B	−0.157729	0.419431	−0.146620	0.093*	0.151 (9)
C40B	−0.210 (3)	0.533 (5)	−0.135 (8)	0.085 (7)	0.151 (9)
C41B	−0.187 (4)	0.612 (4)	−0.114 (8)	0.084 (6)	0.151 (9)
H41B	−0.233727	0.653208	−0.116084	0.101*	0.151 (9)
C42B	−0.096 (5)	0.631 (5)	−0.089 (7)	0.077 (6)	0.151 (9)
H42B	−0.080882	0.685251	−0.073888	0.092*	0.151 (9)
O18B	−0.300 (3)	0.516 (5)	−0.154 (9)	0.095 (15)	0.151 (9)
H18B	−0.320078	0.545931	−0.191201	0.142*	0.151 (9)
O19	0.1728 (8)	0.8272 (7)	−0.2033 (7)	0.036 (3)	
O20	0.1083 (8)	0.7685 (7)	−0.0984 (7)	0.042 (3)	
C43	0.1037 (11)	0.8116 (9)	−0.1622 (9)	0.033 (4)	
C44	0.0110 (7)	0.8429 (9)	−0.1900 (7)	0.032 (4)	
C45	0.0012 (8)	0.8741 (8)	−0.2664 (7)	0.030 (3)	
H45	0.052975	0.875370	−0.299664	0.036*	
C46	−0.0847 (8)	0.9036 (8)	−0.2940 (7)	0.030 (3)	
H46	−0.091907	0.924637	−0.346416	0.036*	
C47	−0.1601 (8)	0.9022 (9)	−0.2450 (7)	0.033 (3)	
C48	−0.1504 (8)	0.8704 (11)	−0.1686 (7)	0.040 (4)	
H48	−0.202477	0.868284	−0.135577	0.048*	
C49	−0.0642 (8)	0.8419 (10)	−0.1410 (7)	0.035 (4)	
H49	−0.056712	0.821583	−0.088273	0.043*	
O21	−0.2449 (8)	0.9304 (8)	−0.2702 (8)	0.047 (3)	
H21O	−0.238122	0.967694	−0.303444	0.071*	
O22	0.2392 (8)	0.9745 (6)	0.0926 (7)	0.031 (2)	
O23	0.1592 (8)	0.8793 (6)	0.0249 (7)	0.036 (3)	
C50	0.1623 (11)	0.9423 (8)	0.0681 (9)	0.031 (3)	
C51	0.0719 (9)	0.9786 (9)	0.0883 (9)	0.028 (3)	
C52	0.0658 (10)	1.0561 (8)	0.1207 (8)	0.025 (3)	
H52	0.121092	1.086494	0.130962	0.030*	
C53	−0.0200 (10)	1.0896 (9)	0.1383 (9)	0.030 (3)	
H53	−0.023111	1.142353	0.160452	0.035*	
C54	−0.1002 (10)	1.0455 (9)	0.1232 (8)	0.031 (3)	
C55	−0.0946 (11)	0.9676 (10)	0.0914 (10)	0.040 (4)	
H55	−0.150018	0.937181	0.081930	0.048*	
C56	−0.0104 (10)	0.9343 (9)	0.0737 (10)	0.032 (4)	
H56	−0.007791	0.881476	0.051759	0.039*	
O24	−0.1870 (8)	1.0765 (8)	0.1350 (7)	0.042 (3)	
H24O	−0.182646	1.126221	0.144980	0.063*	
O29	0.534 (3)	0.5064 (18)	0.072 (2)	0.081 (10)	0.64 (3)
C57	0.551 (3)	0.467 (2)	0.136 (2)	0.079 (7)	0.64 (3)
H57	0.563221	0.499596	0.182111	0.095*	0.64 (3)
N5	0.5544 (15)	0.3896 (13)	0.1484 (13)	0.079 (5)	0.64 (3)
C58	0.548 (4)	0.341 (3)	0.079 (3)	0.100 (10)	0.64 (3)
H58A	0.551354	0.283857	0.093564	0.151*	0.64 (3)
H58B	0.489232	0.352043	0.049507	0.151*	0.64 (3)
H58C	0.600280	0.354158	0.044897	0.151*	0.64 (3)
C59	0.560 (3)	0.353 (3)	0.226 (2)	0.078 (9)	0.64 (3)
H59A	0.591540	0.300613	0.223010	0.117*	0.64 (3)
H59B	0.496707	0.344781	0.244652	0.117*	0.64 (3)
H59C	0.594682	0.388422	0.263137	0.117*	0.64 (3)
O29B	0.558 (7)	0.506 (3)	0.075 (5)	0.083 (13)	0.36 (3)
C57B	0.544 (5)	0.430 (3)	0.082 (2)	0.077 (7)	0.36 (3)
H57B	0.525002	0.400831	0.035292	0.093*	0.36 (3)
N5B	0.5544 (15)	0.3896 (13)	0.1484 (13)	0.079 (5)	0.36 (3)
C58B	0.550 (5)	0.437 (4)	0.219 (3)	0.085 (12)	0.36 (3)
H58D	0.558930	0.401230	0.265523	0.127*	0.36 (3)
H58E	0.599950	0.477426	0.219819	0.127*	0.36 (3)
H58F	0.489716	0.463469	0.220381	0.127*	0.36 (3)
C59B	0.564 (6)	0.305 (2)	0.157 (4)	0.091 (12)	0.36 (3)
H59D	0.564764	0.279721	0.104187	0.136*	0.36 (3)
H59E	0.511996	0.283417	0.186061	0.136*	0.36 (3)
H59F	0.622915	0.292777	0.186364	0.136*	0.36 (3)
O30	0.5200 (10)	0.6114 (9)	−0.2014 (9)	0.059 (4)	
C60	0.5257 (15)	0.5688 (14)	−0.2619 (15)	0.059 (6)	
H60	0.512842	0.512776	−0.256171	0.070*	
N6	0.5476 (11)	0.5933 (11)	−0.3324 (10)	0.049 (4)	
C61	0.5632 (18)	0.6748 (15)	−0.3471 (16)	0.071 (7)	
H61A	0.551564	0.706276	−0.299162	0.085*	
H61B	0.521015	0.693088	−0.390789	0.085*	
H61C	0.627874	0.682628	−0.361852	0.085*	
C62	0.544 (2)	0.535 (2)	−0.3995 (18)	0.094 (9)	
H62A	0.493602	0.549649	−0.437476	0.141*	
H62B	0.533704	0.480279	−0.379198	0.141*	
H62C	0.603721	0.536298	−0.426282	0.141*	
O31	0.5907 (9)	0.8921 (8)	−0.1024 (8)	0.049 (3)	
C63	0.6086 (12)	0.9537 (12)	−0.1448 (12)	0.050 (5)	
H63	0.585549	0.952341	−0.198497	0.060*	
N7	0.6549 (12)	1.0184 (11)	−0.1227 (11)	0.064 (5)	
C64	0.690 (2)	1.025 (2)	−0.0426 (18)	0.115 (14)	
H64A	0.672293	0.976160	−0.013118	0.172*	
H64B	0.663044	1.072559	−0.017871	0.172*	
H64C	0.757741	1.029481	−0.041643	0.172*	
C65	0.6753 (16)	1.0815 (13)	−0.1768 (16)	0.069 (7)	
H65A	0.662379	1.062823	−0.231407	0.104*	
H65B	0.741136	1.096459	−0.169971	0.104*	
H65C	0.636320	1.128648	−0.166189	0.104*	
O32	0.6075 (19)	0.7910 (17)	0.1735 (14)	0.050 (7)	0.51 (2)
C66	0.633 (3)	0.799 (2)	0.248 (2)	0.058 (6)	0.51 (2)
H66	0.632976	0.751106	0.279596	0.070*	0.51 (2)
N8	0.660 (3)	0.867 (2)	0.2832 (19)	0.059 (6)	0.51 (2)
C67	0.664 (4)	0.937 (3)	0.239 (3)	0.069 (9)	0.51 (2)
H67A	0.728462	0.957485	0.240666	0.103*	0.51 (2)
H67B	0.622884	0.977860	0.260172	0.103*	0.51 (2)
H67C	0.644884	0.925393	0.183226	0.103*	0.51 (2)
C68	0.692 (4)	0.859 (3)	0.365 (2)	0.075 (11)	0.51 (2)
H68A	0.758926	0.848350	0.367287	0.112*	0.51 (2)
H68B	0.659183	0.813519	0.389090	0.112*	0.51 (2)
H68C	0.678673	0.908794	0.393418	0.112*	0.51 (2)
O32B	0.637 (3)	0.847 (2)	0.173 (2)	0.076 (8)	0.49 (2)
C66B	0.640 (4)	0.911 (3)	0.219 (2)	0.063 (7)	0.49 (2)
H66B	0.623516	0.962244	0.195981	0.076*	0.49 (2)
N8B	0.664 (3)	0.907 (2)	0.294 (2)	0.058 (6)	0.49 (2)
C67B	0.677 (4)	0.829 (2)	0.328 (3)	0.067 (10)	0.49 (2)
H67D	0.733264	0.804046	0.307132	0.100*	0.49 (2)
H67E	0.623148	0.794713	0.315217	0.100*	0.49 (2)
H67F	0.685459	0.833973	0.386213	0.100*	0.49 (2)
C68B	0.661 (3)	0.976 (2)	0.344 (2)	0.051 (9)	0.49 (2)
H68D	0.658889	1.025445	0.311370	0.076*	0.49 (2)
H68E	0.716703	0.977496	0.379707	0.076*	0.49 (2)
H68F	0.605251	0.974111	0.375919	0.076*	0.49 (2)

### Tetra-µ-aqua-tetrakis{2-[azanidylene(oxido)methyl]phenolato}tetrakis(µ2-3-hydroxybenzoato)dysprosium(III)tetramanganese(III)sodium(I) N,N-dimethylformamide tetrasolvate (2) Atomic displacement parameters (Å^2^)

**Table:**

	*U*^11^	*U*^22^	*U*^33^	*U*^12^	*U*^13^	*U*^23^
Mn2	0.0437 (14)	0.0177 (10)	0.0200 (10)	0.0027 (10)	0.0047 (9)	0.0002 (8)
Mn3	0.0299 (11)	0.0232 (11)	0.0161 (10)	0.0002 (9)	0.0046 (8)	0.0032 (8)
Mn4	0.0341 (12)	0.0179 (10)	0.0206 (10)	0.0012 (9)	0.0034 (9)	0.0003 (8)
Na1	0.042 (3)	0.023 (3)	0.027 (3)	0.003 (3)	−0.008 (3)	−0.004 (2)
O3	0.078 (9)	0.023 (5)	0.034 (6)	−0.002 (6)	0.006 (6)	−0.002 (5)
O5	0.061 (8)	0.017 (5)	0.027 (5)	0.004 (5)	0.008 (5)	−0.006 (4)
O6	0.038 (6)	0.043 (6)	0.020 (5)	−0.011 (5)	0.006 (4)	0.006 (5)
O8	0.035 (6)	0.027 (5)	0.021 (5)	−0.003 (4)	0.005 (4)	0.005 (4)
O9	0.044 (6)	0.022 (5)	0.031 (6)	−0.009 (5)	0.003 (5)	0.000 (4)
O25	0.113 (13)	0.061 (10)	0.045 (8)	0.043 (9)	−0.013 (8)	−0.011 (7)
O26	0.059 (8)	0.046 (7)	0.036 (7)	0.022 (6)	0.006 (6)	0.003 (6)
O27	0.034 (6)	0.044 (7)	0.046 (7)	0.000 (5)	0.008 (5)	−0.008 (6)
O28	0.041 (7)	0.038 (6)	0.032 (6)	0.004 (5)	−0.001 (5)	0.000 (5)
N1	0.037 (7)	0.014 (5)	0.025 (6)	−0.001 (5)	0.005 (5)	0.000 (5)
N3	0.026 (6)	0.012 (5)	0.024 (6)	0.005 (5)	−0.001 (5)	0.000 (4)
C2	0.052 (10)	0.024 (8)	0.030 (8)	−0.005 (7)	−0.004 (7)	0.001 (6)
C3	0.068 (12)	0.037 (9)	0.029 (8)	0.002 (9)	−0.008 (8)	0.011 (7)
C4	0.058 (11)	0.028 (8)	0.057 (12)	−0.002 (8)	−0.004 (9)	0.019 (8)
C5	0.060 (12)	0.033 (9)	0.057 (12)	0.009 (8)	0.014 (10)	0.009 (8)
C6	0.058 (11)	0.019 (7)	0.046 (10)	−0.001 (7)	0.015 (8)	−0.004 (7)
C7	0.050 (10)	0.020 (7)	0.037 (9)	−0.002 (7)	0.008 (7)	−0.003 (6)
C8	0.024 (7)	0.029 (7)	0.024 (6)	0.002 (6)	0.002 (5)	−0.004 (5)
C9	0.028 (8)	0.038 (8)	0.028 (7)	0.002 (7)	0.011 (6)	−0.005 (7)
C10	0.048 (10)	0.045 (10)	0.032 (9)	−0.006 (8)	0.008 (8)	−0.003 (7)
C11	0.067 (14)	0.071 (14)	0.033 (10)	−0.014 (11)	0.016 (9)	−0.021 (10)
C12	0.060 (13)	0.083 (15)	0.024 (8)	−0.011 (11)	0.006 (8)	−0.009 (9)
C13	0.057 (11)	0.056 (11)	0.017 (7)	−0.012 (9)	0.010 (7)	−0.006 (7)
C14	0.035 (9)	0.054 (10)	0.018 (7)	−0.011 (8)	0.004 (6)	−0.006 (7)
C15	0.025 (7)	0.018 (6)	0.028 (7)	0.003 (5)	−0.004 (6)	0.007 (6)
C16	0.026 (8)	0.029 (8)	0.034 (8)	−0.002 (6)	0.005 (6)	0.002 (6)
C17	0.039 (9)	0.022 (8)	0.060 (12)	−0.004 (7)	0.009 (8)	0.006 (7)
C18	0.065 (13)	0.039 (10)	0.050 (11)	−0.018 (9)	0.007 (9)	0.026 (9)
C19	0.085 (15)	0.031 (9)	0.060 (13)	−0.013 (10)	0.032 (12)	0.010 (9)
C20	0.052 (11)	0.027 (8)	0.048 (10)	−0.009 (7)	0.014 (8)	−0.004 (7)
C21	0.028 (8)	0.028 (8)	0.045 (9)	0.000 (6)	0.017 (7)	0.003 (7)
Dy1	0.0381 (12)	0.0309 (4)	0.0258 (6)	0.0046 (7)	0.0024 (7)	0.0003 (4)
O1	0.042 (8)	0.019 (5)	0.016 (5)	0.005 (5)	−0.002 (5)	−0.001 (4)
O2	0.052 (7)	0.028 (6)	0.024 (5)	−0.002 (6)	−0.003 (6)	0.008 (5)
O4	0.037 (7)	0.021 (5)	0.010 (4)	0.009 (5)	0.006 (4)	0.007 (4)
N2	0.030 (6)	0.026 (6)	0.020 (5)	0.017 (5)	−0.006 (4)	−0.007 (4)
C1	0.054 (9)	0.030 (6)	0.022 (6)	0.001 (7)	0.001 (6)	0.001 (5)
N4	0.031 (6)	0.015 (5)	0.016 (5)	0.001 (5)	0.004 (5)	0.000 (4)
O7	0.045 (8)	0.014 (5)	0.014 (5)	−0.005 (5)	0.000 (5)	0.003 (4)
O10	0.021 (6)	0.017 (5)	0.015 (5)	−0.001 (5)	−0.006 (4)	0.007 (4)
O11	0.032 (10)	0.021 (5)	0.022 (5)	−0.001 (5)	0.002 (5)	−0.004 (4)
C22	0.036 (7)	0.022 (5)	0.022 (5)	0.005 (5)	0.010 (5)	0.003 (4)
C23	0.032 (8)	0.029 (6)	0.013 (6)	0.001 (6)	0.007 (5)	0.001 (5)
C24	0.036 (9)	0.041 (7)	0.012 (7)	−0.003 (7)	0.000 (6)	−0.005 (6)
C25	0.042 (10)	0.054 (8)	0.018 (7)	−0.004 (8)	0.000 (7)	−0.007 (7)
C26	0.047 (10)	0.050 (8)	0.021 (7)	−0.009 (8)	−0.005 (7)	−0.006 (7)
C27	0.044 (9)	0.042 (8)	0.016 (6)	−0.001 (7)	−0.004 (6)	0.006 (6)
C28	0.040 (8)	0.036 (7)	0.016 (6)	0.001 (7)	0.000 (6)	0.004 (5)
O12	0.059 (8)	0.030 (6)	0.021 (6)	0.002 (6)	−0.010 (6)	0.001 (5)
Mn1	0.044 (2)	0.0216 (14)	0.0174 (13)	0.0003 (16)	0.0023 (16)	−0.0004 (10)
O13	0.056 (8)	0.035 (6)	0.040 (7)	−0.008 (6)	0.021 (6)	−0.007 (6)
O14	0.051 (8)	0.037 (7)	0.046 (7)	0.004 (7)	0.015 (6)	0.001 (6)
C29	0.069 (9)	0.022 (7)	0.043 (8)	−0.003 (8)	0.024 (8)	−0.005 (7)
C30	0.063 (8)	0.036 (7)	0.055 (8)	−0.003 (7)	0.020 (7)	−0.007 (7)
C31	0.063 (10)	0.028 (8)	0.057 (10)	−0.015 (8)	0.030 (8)	−0.003 (8)
C32	0.063 (9)	0.034 (8)	0.055 (9)	−0.013 (8)	0.025 (8)	0.004 (8)
C33	0.062 (9)	0.035 (9)	0.060 (9)	−0.007 (8)	0.024 (8)	0.008 (9)
C34	0.068 (9)	0.046 (8)	0.061 (9)	−0.005 (8)	0.022 (8)	0.006 (8)
C35	0.063 (9)	0.046 (8)	0.056 (9)	−0.008 (8)	0.025 (8)	0.001 (8)
O15	0.071 (10)	0.051 (9)	0.071 (10)	0.004 (8)	0.026 (9)	0.014 (8)
Dy1B	0.0381 (12)	0.0309 (4)	0.0258 (6)	0.0046 (7)	0.0024 (7)	0.0003 (4)
O1B	0.034 (17)	0.019 (14)	0.013 (14)	0.002 (15)	0.005 (15)	0.000 (13)
O2B	0.048 (11)	0.025 (10)	0.018 (10)	−0.003 (10)	0.001 (10)	0.005 (10)
O4B	0.037 (7)	0.021 (5)	0.010 (4)	0.009 (5)	0.006 (4)	0.007 (4)
N2B	0.030 (6)	0.026 (6)	0.020 (5)	0.017 (5)	−0.006 (4)	−0.007 (4)
C1B	0.054 (9)	0.030 (6)	0.022 (6)	0.001 (7)	0.001 (6)	0.001 (5)
N4B	0.032 (10)	0.016 (9)	0.019 (9)	0.002 (10)	0.001 (10)	0.004 (9)
O7B	0.045 (8)	0.014 (5)	0.014 (5)	−0.005 (5)	0.000 (5)	0.003 (4)
O10B	0.026 (14)	0.017 (12)	0.017 (12)	0.002 (13)	−0.001 (13)	0.001 (12)
O11B	0.033 (15)	0.022 (12)	0.023 (12)	0.002 (12)	0.014 (12)	0.001 (12)
C22B	0.036 (7)	0.022 (5)	0.022 (5)	0.005 (5)	0.010 (5)	0.003 (4)
C23B	0.038 (10)	0.032 (9)	0.016 (9)	0.000 (9)	0.004 (9)	0.001 (9)
C24B	0.038 (12)	0.042 (11)	0.016 (11)	0.000 (11)	0.003 (10)	−0.001 (10)
C25B	0.041 (12)	0.047 (11)	0.017 (11)	−0.004 (11)	−0.001 (11)	−0.003 (10)
C26B	0.042 (12)	0.047 (11)	0.018 (10)	−0.003 (11)	−0.004 (11)	−0.002 (10)
C27B	0.044 (12)	0.042 (11)	0.016 (10)	−0.002 (11)	−0.003 (11)	0.004 (10)
C28B	0.043 (10)	0.035 (9)	0.016 (9)	−0.001 (9)	−0.001 (9)	0.003 (9)
O12B	0.040 (11)	0.027 (10)	0.014 (10)	0.001 (10)	0.001 (10)	0.002 (9)
Mn1B	0.037 (8)	0.015 (6)	0.015 (6)	0.000 (7)	0.003 (7)	0.005 (5)
O13B	0.060 (11)	0.032 (11)	0.047 (11)	0.000 (11)	0.017 (11)	−0.009 (10)
O14B	0.060 (15)	0.026 (13)	0.055 (14)	−0.003 (14)	0.017 (14)	0.002 (13)
C29B	0.059 (10)	0.032 (9)	0.050 (9)	−0.003 (9)	0.018 (9)	−0.003 (9)
C30B	0.063 (11)	0.039 (11)	0.055 (11)	−0.006 (10)	0.021 (10)	0.002 (10)
C31B	0.063 (9)	0.038 (9)	0.057 (9)	−0.008 (9)	0.024 (9)	0.002 (9)
C32B	0.064 (11)	0.038 (11)	0.060 (11)	−0.007 (11)	0.024 (11)	0.006 (11)
C33B	0.066 (12)	0.040 (12)	0.061 (12)	−0.004 (12)	0.023 (12)	0.009 (12)
C34B	0.064 (13)	0.043 (13)	0.058 (13)	−0.008 (13)	0.022 (13)	0.005 (13)
C35B	0.063 (13)	0.043 (13)	0.056 (13)	−0.008 (13)	0.021 (13)	0.007 (13)
O15B	0.09 (3)	0.04 (3)	0.07 (3)	0.00 (3)	0.03 (3)	0.02 (3)
O16	0.060 (8)	0.035 (7)	0.030 (6)	−0.010 (6)	0.011 (6)	0.006 (6)
O17	0.069 (9)	0.034 (7)	0.034 (7)	−0.007 (6)	0.017 (6)	0.004 (6)
C36	0.082 (9)	0.028 (7)	0.037 (8)	−0.002 (8)	0.005 (8)	0.009 (7)
C37	0.089 (9)	0.048 (8)	0.053 (9)	0.003 (8)	0.016 (8)	0.009 (8)
C38	0.095 (11)	0.058 (10)	0.055 (9)	0.001 (9)	0.014 (9)	0.011 (9)
C39	0.099 (11)	0.076 (11)	0.061 (10)	−0.004 (10)	0.015 (10)	0.014 (10)
C40	0.103 (11)	0.077 (11)	0.077 (11)	0.003 (10)	0.005 (10)	0.013 (10)
C41	0.104 (12)	0.075 (12)	0.088 (12)	0.012 (11)	0.010 (11)	0.004 (11)
C42	0.101 (11)	0.063 (10)	0.074 (11)	0.008 (10)	0.010 (10)	0.008 (10)
O18	0.101 (15)	0.143 (19)	0.119 (18)	0.014 (15)	−0.013 (14)	0.021 (15)
O16B	0.069 (12)	0.030 (12)	0.032 (12)	−0.004 (12)	0.010 (11)	0.008 (12)
O17B	0.09 (3)	0.05 (2)	0.05 (2)	−0.01 (2)	−0.01 (2)	0.01 (2)
C36B	0.078 (11)	0.035 (10)	0.038 (10)	−0.004 (10)	0.011 (10)	0.009 (9)
C37B	0.091 (10)	0.051 (10)	0.055 (10)	0.002 (10)	0.011 (10)	0.009 (10)
C38B	0.095 (12)	0.060 (12)	0.057 (12)	−0.001 (12)	0.014 (11)	0.012 (12)
C39B	0.100 (13)	0.071 (13)	0.063 (12)	0.001 (12)	0.011 (12)	0.013 (12)
C40B	0.103 (13)	0.080 (14)	0.072 (13)	0.004 (13)	0.010 (13)	0.014 (13)
C41B	0.102 (12)	0.076 (12)	0.075 (12)	0.006 (11)	0.009 (11)	0.010 (11)
C42B	0.098 (12)	0.065 (11)	0.069 (12)	0.007 (11)	0.011 (11)	0.009 (11)
O18B	0.10 (3)	0.09 (3)	0.09 (3)	0.01 (3)	0.00 (3)	0.00 (3)
O19	0.027 (6)	0.033 (6)	0.049 (7)	0.000 (5)	0.007 (5)	0.003 (5)
O20	0.037 (6)	0.046 (7)	0.044 (7)	−0.003 (5)	0.008 (5)	0.008 (6)
C43	0.043 (10)	0.025 (8)	0.032 (8)	−0.004 (7)	0.003 (7)	−0.006 (6)
C44	0.016 (7)	0.034 (8)	0.045 (10)	−0.001 (6)	0.002 (7)	−0.005 (7)
C45	0.031 (8)	0.016 (6)	0.043 (9)	0.003 (6)	0.003 (7)	−0.003 (6)
C46	0.048 (10)	0.017 (6)	0.024 (7)	−0.003 (6)	−0.005 (7)	0.001 (5)
C47	0.031 (8)	0.028 (8)	0.042 (9)	−0.002 (6)	0.006 (7)	−0.003 (7)
C48	0.028 (8)	0.055 (11)	0.038 (9)	0.007 (8)	0.006 (7)	0.001 (8)
C49	0.034 (9)	0.050 (10)	0.022 (7)	−0.002 (7)	−0.003 (6)	0.005 (7)
O21	0.038 (7)	0.053 (8)	0.051 (8)	0.006 (6)	0.000 (6)	0.021 (6)
O22	0.038 (6)	0.023 (5)	0.031 (6)	0.008 (5)	0.004 (5)	−0.003 (4)
O23	0.039 (6)	0.023 (5)	0.045 (7)	0.009 (5)	0.001 (5)	−0.013 (5)
C50	0.050 (10)	0.020 (7)	0.020 (7)	0.002 (7)	−0.009 (7)	0.005 (6)
C51	0.029 (8)	0.030 (8)	0.025 (7)	0.001 (6)	−0.003 (6)	0.002 (6)
C52	0.031 (8)	0.022 (7)	0.022 (7)	0.005 (6)	0.004 (6)	−0.001 (5)
C53	0.039 (9)	0.026 (7)	0.023 (7)	0.005 (7)	−0.004 (6)	0.000 (6)
C54	0.033 (8)	0.038 (8)	0.021 (7)	0.008 (7)	0.010 (6)	−0.001 (6)
C55	0.033 (9)	0.052 (11)	0.035 (9)	−0.006 (8)	−0.004 (7)	−0.013 (8)
C56	0.040 (9)	0.025 (8)	0.031 (8)	0.004 (7)	0.003 (7)	−0.009 (6)
O24	0.037 (6)	0.057 (8)	0.033 (6)	0.007 (6)	0.001 (5)	−0.019 (6)
O29	0.067 (19)	0.091 (15)	0.083 (15)	0.041 (12)	0.006 (14)	0.041 (13)
C57	0.071 (11)	0.082 (11)	0.085 (11)	0.016 (10)	0.004 (10)	0.027 (10)
N5	0.070 (8)	0.081 (8)	0.087 (8)	0.014 (7)	−0.001 (7)	0.029 (7)
C58	0.086 (18)	0.105 (19)	0.109 (19)	0.010 (17)	0.002 (17)	0.010 (17)
C59	0.072 (16)	0.076 (17)	0.086 (17)	−0.001 (15)	0.003 (15)	0.032 (15)
O29B	0.08 (2)	0.09 (2)	0.09 (2)	0.023 (18)	0.001 (18)	0.046 (18)
C57B	0.072 (12)	0.077 (12)	0.084 (12)	0.012 (11)	0.000 (11)	0.028 (11)
N5B	0.070 (8)	0.081 (8)	0.087 (8)	0.014 (7)	−0.001 (7)	0.029 (7)
C58B	0.069 (19)	0.09 (2)	0.09 (2)	0.013 (18)	−0.003 (18)	0.018 (19)
C59B	0.08 (2)	0.09 (2)	0.11 (2)	0.018 (19)	−0.002 (19)	0.030 (19)
O30	0.067 (10)	0.063 (9)	0.047 (8)	0.015 (7)	0.006 (7)	−0.010 (7)
C60	0.048 (12)	0.053 (12)	0.076 (16)	0.005 (9)	0.011 (11)	0.008 (12)
N6	0.041 (9)	0.057 (10)	0.049 (9)	0.016 (7)	0.010 (7)	0.001 (8)
C61	0.071 (15)	0.062 (14)	0.081 (17)	0.001 (11)	0.028 (13)	0.017 (13)
C62	0.11 (2)	0.10 (2)	0.078 (19)	0.032 (18)	0.035 (17)	−0.004 (17)
O31	0.039 (7)	0.062 (9)	0.048 (7)	0.003 (6)	0.006 (6)	0.008 (7)
C63	0.032 (9)	0.065 (13)	0.054 (11)	−0.001 (9)	0.012 (8)	−0.008 (10)
N7	0.045 (10)	0.077 (13)	0.070 (12)	−0.021 (9)	0.007 (9)	−0.013 (11)
C64	0.069 (19)	0.14 (3)	0.13 (3)	0.002 (18)	−0.033 (19)	−0.07 (3)
C65	0.050 (12)	0.053 (13)	0.11 (2)	−0.009 (10)	0.029 (13)	−0.007 (13)
O32	0.055 (15)	0.048 (15)	0.045 (13)	0.002 (12)	−0.021 (11)	0.011 (12)
C66	0.047 (12)	0.067 (15)	0.060 (13)	−0.003 (12)	−0.012 (11)	−0.001 (12)
N8	0.048 (11)	0.071 (15)	0.055 (13)	0.000 (13)	−0.015 (10)	−0.002 (12)
C67	0.056 (18)	0.070 (19)	0.078 (19)	−0.007 (16)	−0.018 (16)	0.000 (17)
C68	0.07 (2)	0.09 (2)	0.06 (2)	−0.01 (2)	−0.014 (19)	0.00 (2)
O32B	0.072 (16)	0.085 (18)	0.069 (16)	0.013 (15)	−0.019 (14)	−0.001 (15)
C66B	0.053 (13)	0.070 (16)	0.064 (14)	0.003 (14)	−0.016 (12)	−0.002 (13)
N8B	0.043 (11)	0.068 (15)	0.062 (13)	−0.005 (13)	−0.013 (11)	0.003 (13)
C67B	0.067 (19)	0.06 (2)	0.07 (2)	−0.005 (18)	−0.011 (19)	0.000 (18)
C68B	0.026 (16)	0.048 (18)	0.08 (2)	−0.007 (14)	−0.011 (16)	0.024 (17)

### Tetra-µ-aqua-tetrakis{2-[azanidylene(oxido)methyl]phenolato}tetrakis(µ2-3-hydroxybenzoato)dysprosium(III)tetramanganese(III)sodium(I) N,N-dimethylformamide tetrasolvate (2) Geometric parameters (Å, º)

**Table:**

Mn2—O4B	1.83 (7)	C22B—C23B	1.49 (4)
Mn2—O3	1.854 (12)	C23B—C24B	1.3900
Mn2—O4	1.910 (14)	C23B—C28B	1.3900
Mn2—O5	1.939 (11)	C24B—C25B	1.3900
Mn2—N1	1.960 (13)	C24B—H24B	0.9500
Mn2—O16	2.154 (15)	C25B—C26B	1.3900
Mn2—O16B	2.20 (4)	C25B—H25B	0.9500
Mn2—O26	2.393 (15)	C26B—C27B	1.3900
Mn2—Na1	3.726 (6)	C26B—H26B	0.9500
Mn3—O7B	1.83 (6)	C27B—C28B	1.3900
Mn3—O6	1.853 (11)	C27B—H27B	0.9500
Mn3—O7	1.904 (13)	C28B—O12B	1.36 (4)
Mn3—N2	1.91 (2)	O12B—Mn1B	1.86 (3)
Mn3—O8	1.944 (10)	Mn1B—O13B	2.15 (4)
Mn3—O19	2.127 (11)	O13B—C29B	1.26 (3)
Mn3—N2B	2.15 (11)	O14B—C29B	1.29 (3)
Mn3—O27	2.447 (13)	C29B—C30B	1.49 (3)
Mn3—Na1	3.691 (6)	C30B—C31B	1.40 (3)
Mn4—O9	1.833 (10)	C30B—C35B	1.41 (3)
Mn4—O10	1.901 (12)	C31B—C32B	1.39 (3)
Mn4—O11B	1.90 (4)	C31B—H31B	0.9500
Mn4—O11	1.952 (12)	C32B—C33B	1.39 (3)
Mn4—N3	1.969 (12)	C32B—H32B	0.9500
Mn4—O10B	2.07 (4)	C33B—O15B	1.37 (3)
Mn4—O22	2.230 (11)	C33B—C34B	1.40 (3)
Mn4—O28	2.446 (12)	C34B—C35B	1.38 (3)
Mn4—C22B	2.64 (5)	C34B—H34B	0.9500
Mn4—Na1	3.651 (6)	C35B—H35B	0.9500
Na1—O25	2.396 (14)	O15B—H15B	0.8400
Na1—O28	2.460 (13)	O16—C36	1.24 (2)
Na1—O27	2.486 (15)	O17—C36	1.28 (2)
Na1—O26	2.519 (15)	C36—C37	1.49 (3)
Na1—O10	2.682 (14)	C37—C38	1.40 (3)
Na1—O1	2.704 (15)	C37—C42	1.41 (3)
Na1—O7	2.725 (18)	C38—C39	1.40 (3)
Na1—O4	2.740 (15)	C38—H38	0.9500
Na1—O7B	2.85 (10)	C39—C40	1.37 (3)
Na1—O10B	2.93 (7)	C39—H39	0.9500
Na1—Dy1	3.583 (7)	C40—O18	1.36 (3)
Na1—Mn1	3.621 (7)	C40—C41	1.39 (3)
O3—C7	1.37 (2)	C41—C42	1.37 (3)
O5—C8	1.299 (18)	C41—H41	0.9500
O6—C14	1.37 (2)	C42—H42	0.9500
O8—C15	1.287 (18)	O18—H18O	0.8400
O9—C21	1.317 (19)	O16B—C36B	1.26 (2)
O25—Mn1	2.435 (19)	O17B—C36B	1.29 (2)
O25—H25C	0.9200	C36B—C37B	1.488 (19)
O25—H25D	0.8696	C37B—C38B	1.3900
O26—H26C	0.85 (4)	C37B—C42B	1.3900
O26—H26D	0.84 (4)	C38B—C39B	1.3900
O27—H27C	0.87 (4)	C38B—H38B	0.9500
O27—H27D	0.87 (4)	C39B—C40B	1.3900
O28—H28C	0.88 (4)	C39B—H39B	0.9500
O28—H28D	0.88 (4)	C40B—O18B	1.355 (17)
N1—C1B	1.30 (4)	C40B—C41B	1.3900
N1—C1	1.30 (2)	C41B—C42B	1.3900
N1—O1	1.394 (16)	C41B—H41B	0.9500
N1—O1B	1.45 (4)	C42B—H42B	0.9500
N3—C15	1.319 (18)	O18B—H18B	0.8400
N3—O7	1.395 (17)	O19—C43	1.258 (17)
N3—O7B	1.63 (8)	O20—C43	1.288 (18)
C2—C3	1.41 (2)	C43—C44	1.485 (18)
C2—C7	1.42 (2)	C44—C49	1.385 (9)
C2—C1	1.48 (2)	C44—C45	1.387 (9)
C2—C1B	1.51 (4)	C45—C46	1.388 (9)
C3—C4	1.33 (2)	C45—H45	0.9500
C3—H3	0.9500	C46—C47	1.387 (9)
C4—C5	1.39 (3)	C46—H46	0.9500
C4—H4	0.9500	C47—O21	1.356 (15)
C5—C6	1.40 (3)	C47—C48	1.390 (9)
C5—H5	0.9500	C48—C49	1.388 (9)
C6—C7	1.39 (2)	C48—H48	0.9500
C6—H6	0.9500	C49—H49	0.9500
C8—N2B	1.18 (8)	O21—H21O	0.8400
C8—N2	1.31 (2)	O22—C50	1.280 (18)
C8—C9	1.49 (2)	O23—C50	1.268 (16)
C9—C14	1.38 (2)	C50—C51	1.482 (19)
C9—C10	1.38 (2)	C51—C52	1.395 (18)
C10—C11	1.39 (2)	C51—C56	1.402 (19)
C10—H10	0.9500	C52—C53	1.393 (18)
C11—C12	1.37 (3)	C52—H52	0.9500
C11—H11	0.9500	C53—C54	1.38 (2)
C12—C13	1.38 (3)	C53—H53	0.9500
C12—H12	0.9500	C54—O24	1.370 (17)
C13—C14	1.41 (2)	C54—C55	1.40 (2)
C13—H13	0.9500	C55—C56	1.37 (2)
C15—C16	1.47 (2)	C55—H55	0.9500
C16—C17	1.40 (2)	C56—H56	0.9500
C16—C21	1.42 (2)	O24—H24O	0.8400
C17—C18	1.39 (2)	O29—C57	1.28 (3)
C17—H17	0.9500	C57—N5	1.30 (3)
C18—C19	1.31 (3)	C57—H57	0.9500
C18—H18	0.9500	N5—C58	1.42 (3)
C19—C20	1.38 (3)	N5—C59	1.44 (3)
C19—H19	0.9500	C58—H58A	0.9800
C20—C21	1.43 (2)	C58—H58B	0.9800
C20—H20	0.9500	C58—H58C	0.9800
Dy1—O14	2.250 (14)	C59—H59A	0.9800
Dy1—O17	2.332 (14)	C59—H59B	0.9800
Dy1—O10	2.425 (12)	C59—H59C	0.9800
Dy1—O7	2.438 (11)	O29B—C57B	1.28 (3)
Dy1—O1	2.439 (12)	C57B—N5B	1.31 (3)
Dy1—O4	2.440 (10)	C57B—H57B	0.9500
O1—Mn1	1.906 (12)	N5B—C59B	1.41 (3)
O2—C1	1.29 (2)	N5B—C58B	1.42 (3)
O2—Mn1	1.938 (12)	C58B—H58D	0.9800
O4—N2	1.427 (17)	C58B—H58E	0.9800
N4—C22	1.33 (2)	C58B—H58F	0.9800
N4—O10	1.387 (16)	C59B—H59D	0.9800
N4—Mn1	1.944 (13)	C59B—H59E	0.9800
O11—C22	1.285 (19)	C59B—H59F	0.9800
C22—C23	1.47 (2)	O30—C60	1.24 (3)
C23—C24	1.39 (2)	C60—N6	1.30 (3)
C23—C28	1.42 (2)	C60—H60	0.9500
C24—C25	1.40 (2)	N6—C61	1.39 (3)
C24—H24	0.9500	N6—C62	1.48 (3)
C25—C26	1.35 (2)	C61—H61A	0.9800
C25—H25	0.9500	C61—H61B	0.9800
C26—C27	1.39 (2)	C61—H61C	0.9800
C26—H26	0.9500	C62—H62A	0.9800
C27—C28	1.40 (2)	C62—H62B	0.9800
C27—H27	0.9500	C62—H62C	0.9800
C28—O12	1.34 (2)	O31—C63	1.27 (2)
O12—Mn1	1.859 (13)	C63—N7	1.30 (2)
Mn1—O13	2.147 (16)	C63—H63	0.9500
O13—C29	1.25 (2)	N7—C64	1.42 (3)
O14—C29	1.28 (2)	N7—C65	1.42 (2)
C29—C30	1.49 (2)	C64—H64A	0.9800
C30—C35	1.38 (3)	C64—H64B	0.9800
C30—C31	1.41 (2)	C64—H64C	0.9800
C31—C32	1.38 (2)	C65—H65A	0.9800
C31—H31	0.9500	C65—H65B	0.9800
C32—C33	1.40 (3)	C65—H65C	0.9800
C32—H32	0.9500	O32—C66	1.29 (3)
C33—O15	1.37 (2)	C66—N8	1.33 (3)
C33—C34	1.40 (3)	C66—H66	0.9500
C34—C35	1.39 (2)	N8—C67	1.39 (4)
C34—H34	0.9500	N8—C68	1.43 (3)
C35—H35	0.9500	C67—H67A	0.9800
O15—H15O	0.8400	C67—H67B	0.9800
Dy1B—O20	1.900 (18)	C67—H67C	0.9800
Dy1B—O23	2.193 (15)	C68—H68A	0.9800
Dy1B—O14B	2.23 (3)	C68—H68B	0.9800
Dy1B—O7B	2.43 (4)	C68—H68C	0.9800
Dy1B—O4B	2.45 (4)	O32B—C66B	1.31 (4)
Dy1B—O10B	2.45 (4)	C66B—N8B	1.30 (3)
Dy1B—O1B	2.48 (3)	C66B—H66B	0.9500
O1B—Mn1B	1.92 (3)	N8B—C67B	1.42 (4)
O2B—C1B	1.30 (4)	N8B—C68B	1.43 (3)
O2B—Mn1B	1.93 (3)	C67B—H67D	0.9800
O4B—N2B	1.43 (4)	C67B—H67E	0.9800
N4B—C22B	1.31 (4)	C67B—H67F	0.9800
N4B—O10B	1.40 (4)	C68B—H68D	0.9800
N4B—Mn1B	1.94 (3)	C68B—H68E	0.9800
O11B—C22B	1.28 (4)	C68B—H68F	0.9800
			
O4B—Mn2—O3	177 (3)	O12—Mn1—Na1	124.8 (5)
O3—Mn2—O4	172.9 (6)	O1—Mn1—Na1	46.9 (4)
O4B—Mn2—O5	83.0 (17)	O2—Mn1—Na1	102.9 (4)
O3—Mn2—O5	97.0 (5)	N4—Mn1—Na1	65.7 (4)
O4—Mn2—O5	82.0 (5)	O13—Mn1—Na1	135.6 (5)
O4B—Mn2—N1	89.8 (18)	O25—Mn1—Na1	41.0 (3)
O3—Mn2—N1	90.8 (5)	C29—O13—Mn1	124.3 (12)
O4—Mn2—N1	89.2 (5)	C29—O14—Dy1	140.1 (13)
O5—Mn2—N1	168.0 (5)	O13—C29—O14	124.2 (18)
O3—Mn2—O16	90.5 (6)	O13—C29—C30	119.0 (17)
O4—Mn2—O16	96.5 (6)	O14—C29—C30	116.8 (19)
O5—Mn2—O16	90.1 (5)	C35—C30—C31	120.6 (18)
N1—Mn2—O16	99.0 (5)	C35—C30—C29	120.7 (18)
O4B—Mn2—O16B	65 (3)	C31—C30—C29	118.7 (18)
O3—Mn2—O16B	111 (2)	C32—C31—C30	119 (2)
O5—Mn2—O16B	90.8 (18)	C32—C31—H31	120.7
N1—Mn2—O16B	95.0 (19)	C30—C31—H31	120.7
O4B—Mn2—O26	90 (3)	C31—C32—C33	121.5 (19)
O3—Mn2—O26	93.5 (5)	C31—C32—H32	119.2
O4—Mn2—O26	79.4 (5)	C33—C32—H32	119.2
O5—Mn2—O26	85.3 (5)	O15—C33—C32	123.0 (19)
N1—Mn2—O26	85.1 (5)	O15—C33—C34	118 (2)
O16—Mn2—O26	174.2 (5)	C32—C33—C34	119.1 (19)
O16B—Mn2—O26	155 (2)	C35—C34—C33	120 (2)
O4B—Mn2—Na1	55 (3)	C35—C34—H34	120.2
O3—Mn2—Na1	128.7 (5)	C33—C34—H34	120.2
O4—Mn2—Na1	45.3 (4)	C30—C35—C34	120 (2)
O5—Mn2—Na1	101.5 (3)	C30—C35—H35	119.8
N1—Mn2—Na1	66.5 (4)	C34—C35—H35	119.8
O16—Mn2—Na1	136.4 (4)	C33—O15—H15O	109.5
O16B—Mn2—Na1	116 (2)	O20—Dy1B—O23	87.6 (7)
O26—Mn2—Na1	42.0 (3)	O20—Dy1B—O14B	123.8 (19)
O7B—Mn3—O6	175 (2)	O23—Dy1B—O14B	77.7 (18)
O6—Mn3—O7	174.1 (6)	O20—Dy1B—O7B	80 (2)
O6—Mn3—N2	90.8 (6)	O23—Dy1B—O7B	78 (2)
O7—Mn3—N2	90.0 (6)	O14B—Dy1B—O7B	145 (3)
O7B—Mn3—O8	88 (2)	O20—Dy1B—O4B	82.8 (19)
O6—Mn3—O8	96.2 (5)	O23—Dy1B—O4B	141 (2)
O7—Mn3—O8	82.1 (5)	O14B—Dy1B—O4B	137 (2)
N2—Mn3—O8	168.3 (9)	O7B—Dy1B—O4B	63.6 (19)
O7B—Mn3—O19	88 (3)	O20—Dy1B—O10B	139.6 (13)
O6—Mn3—O19	91.3 (5)	O23—Dy1B—O10B	67.5 (16)
O8—Mn3—O19	93.2 (5)	O14B—Dy1B—O10B	83 (2)
O7B—Mn3—N2B	85 (3)	O7B—Dy1B—O10B	64.9 (19)
O6—Mn3—N2B	90.8 (16)	O4B—Dy1B—O10B	97 (2)
O8—Mn3—N2B	170 (4)	O20—Dy1B—O1B	143.5 (11)
O19—Mn3—N2B	93 (4)	O23—Dy1B—O1B	127.4 (11)
O7B—Mn3—O27	86 (3)	O14B—Dy1B—O1B	79.2 (18)
O6—Mn3—O27	94.5 (5)	O7B—Dy1B—O1B	96 (2)
O7—Mn3—O27	79.8 (6)	O4B—Dy1B—O1B	63.6 (18)
N2—Mn3—O27	84.7 (9)	O10B—Dy1B—O1B	63.2 (15)
O8—Mn3—O27	85.4 (4)	O20—Dy1B—Na1	116.8 (6)
O19—Mn3—O27	174.2 (5)	O23—Dy1B—Na1	104.2 (5)
N2B—Mn3—O27	87 (4)	O14B—Dy1B—Na1	119.4 (18)
O7B—Mn3—Na1	49 (3)	O7B—Dy1B—Na1	45 (2)
O6—Mn3—Na1	129.8 (4)	O4B—Dy1B—Na1	50 (2)
O7—Mn3—Na1	45.7 (5)	O10B—Dy1B—Na1	47.3 (16)
N2—Mn3—Na1	66.6 (8)	O1B—Dy1B—Na1	51.2 (14)
O8—Mn3—Na1	101.8 (3)	N1—O1B—Mn1B	110.9 (19)
O19—Mn3—Na1	133.2 (3)	N1—O1B—Dy1B	129 (2)
N2B—Mn3—Na1	68 (4)	Mn1B—O1B—Dy1B	119.0 (18)
O27—Mn3—Na1	41.9 (3)	C1B—O2B—Mn1B	113 (3)
O9—Mn4—O10	169.6 (5)	N2B—O4B—Mn2	106 (4)
O9—Mn4—O11B	102.7 (16)	N2B—O4B—Dy1B	123 (5)
O9—Mn4—O11	97.7 (5)	Mn2—O4B—Dy1B	129 (3)
O10—Mn4—O11	81.3 (5)	C8—N2B—O4B	123 (8)
O9—Mn4—N3	89.9 (5)	C8—N2B—Mn3	123 (6)
O10—Mn4—N3	89.6 (5)	O4B—N2B—Mn3	112 (4)
O11B—Mn4—N3	167.3 (16)	N1—C1B—O2B	121 (3)
O11—Mn4—N3	168.5 (6)	N1—C1B—C2	117 (3)
O9—Mn4—O10B	175.9 (11)	O2B—C1B—C2	123 (3)
O11B—Mn4—O10B	81.3 (18)	C22B—N4B—O10B	110 (3)
N3—Mn4—O10B	86.1 (12)	C22B—N4B—Mn1B	128 (4)
O9—Mn4—O22	91.6 (5)	O10B—N4B—Mn1B	116 (3)
O11B—Mn4—O22	82 (5)	N3—O7B—Mn3	106 (2)
N3—Mn4—O22	99.3 (4)	N3—O7B—Dy1B	121 (4)
O10B—Mn4—O22	88.1 (19)	Mn3—O7B—Dy1B	127 (4)
O9—Mn4—O28	90.3 (5)	N3—O7B—Na1	94 (4)
O10—Mn4—O28	79.4 (5)	Mn3—O7B—Na1	102 (4)
O11B—Mn4—O28	93 (5)	Dy1B—O7B—Na1	97 (2)
O11—Mn4—O28	86.2 (7)	N4B—O10B—Mn4	109 (2)
N3—Mn4—O28	85.2 (4)	N4B—O10B—Dy1B	119 (3)
O10B—Mn4—O28	90.3 (19)	Mn4—O10B—Dy1B	126 (2)
O22—Mn4—O28	175.2 (4)	N4B—O10B—Na1	108 (4)
O9—Mn4—C22B	129.3 (11)	Mn4—O10B—Na1	92 (2)
O11B—Mn4—C22B	26.9 (14)	Dy1B—O10B—Na1	94.8 (16)
N3—Mn4—C22B	140.4 (10)	C22B—O11B—Mn4	110 (4)
O10B—Mn4—C22B	54.7 (13)	O11B—C22B—N4B	127 (4)
O22—Mn4—C22B	87 (2)	O11B—C22B—C23B	112 (4)
O28—Mn4—C22B	89 (2)	N4B—C22B—C23B	121 (4)
O9—Mn4—Na1	125.6 (4)	O11B—C22B—Mn4	43 (2)
O10—Mn4—Na1	45.4 (4)	N4B—C22B—Mn4	86 (3)
O11B—Mn4—Na1	104 (3)	C23B—C22B—Mn4	153 (3)
O11—Mn4—Na1	102.0 (6)	C24B—C23B—C28B	120.0
N3—Mn4—Na1	66.5 (3)	C24B—C23B—C22B	116 (3)
O10B—Mn4—Na1	53.4 (19)	C28B—C23B—C22B	124 (3)
O22—Mn4—Na1	138.3 (3)	C23B—C24B—C25B	120.0
O28—Mn4—Na1	42.1 (3)	C23B—C24B—H24B	120.0
C22B—Mn4—Na1	83.1 (18)	C25B—C24B—H24B	120.0
O25—Na1—O28	86.1 (5)	C26B—C25B—C24B	120.0
O25—Na1—O27	152.0 (6)	C26B—C25B—H25B	120.0
O28—Na1—O27	87.2 (4)	C24B—C25B—H25B	120.0
O25—Na1—O26	87.5 (5)	C25B—C26B—C27B	120.0
O28—Na1—O26	155.4 (5)	C25B—C26B—H26B	120.0
O27—Na1—O26	87.4 (4)	C27B—C26B—H26B	120.0
O25—Na1—O10	82.8 (5)	C28B—C27B—C26B	120.0
O28—Na1—O10	66.0 (4)	C28B—C27B—H27B	120.0
O27—Na1—O10	118.8 (4)	C26B—C27B—H27B	120.0
O26—Na1—O10	136.5 (5)	O12B—C28B—C27B	117 (3)
O25—Na1—O1	67.2 (6)	O12B—C28B—C23B	123 (3)
O28—Na1—O1	119.7 (4)	C27B—C28B—C23B	120.0
O27—Na1—O1	138.2 (5)	C28B—O12B—Mn1B	130 (3)
O26—Na1—O1	79.1 (4)	O12B—Mn1B—O1B	169 (3)
O10—Na1—O1	58.0 (4)	O12B—Mn1B—O2B	97 (2)
O25—Na1—O7	140.2 (5)	O1B—Mn1B—O2B	80.7 (18)
O28—Na1—O7	82.7 (4)	O12B—Mn1B—N4B	92 (2)
O27—Na1—O7	65.3 (4)	O1B—Mn1B—N4B	88.1 (18)
O26—Na1—O7	116.5 (5)	O2B—Mn1B—N4B	167 (2)
O10—Na1—O7	57.8 (4)	O12B—Mn1B—O13B	96 (2)
O1—Na1—O7	85.7 (4)	O1B—Mn1B—O13B	95 (2)
O25—Na1—O4	120.3 (6)	O2B—Mn1B—O13B	91 (3)
O28—Na1—O4	138.9 (4)	N4B—Mn1B—O13B	97 (3)
O27—Na1—O4	81.4 (4)	C29B—O13B—Mn1B	125 (4)
O26—Na1—O4	63.5 (4)	C29B—O14B—Dy1B	141 (6)
O10—Na1—O4	85.4 (4)	O13B—C29B—O14B	123 (4)
O1—Na1—O4	57.2 (4)	O13B—C29B—C30B	123 (4)
O7—Na1—O4	56.7 (4)	O14B—C29B—C30B	113 (4)
O25—Na1—O7B	139.8 (12)	C31B—C30B—C35B	119 (4)
O28—Na1—O7B	88.0 (16)	C31B—C30B—C29B	120 (5)
O27—Na1—O7B	66.9 (10)	C35B—C30B—C29B	117 (4)
O26—Na1—O7B	111.8 (17)	C32B—C31B—C30B	118 (4)
O25—Na1—O10B	86.6 (9)	C32B—C31B—H31B	121.1
O28—Na1—O10B	72.5 (8)	C30B—C31B—H31B	121.1
O27—Na1—O10B	117.1 (10)	C33B—C32B—C31B	123 (5)
O26—Na1—O10B	130.8 (9)	C33B—C32B—H32B	118.7
O7B—Na1—O10B	53.9 (14)	C31B—C32B—H32B	118.7
O25—Na1—Dy1	104.6 (5)	O15B—C33B—C32B	122 (5)
O28—Na1—Dy1	103.7 (3)	O15B—C33B—C34B	119 (5)
O27—Na1—Dy1	103.4 (3)	C32B—C33B—C34B	116 (4)
O26—Na1—Dy1	100.9 (4)	C35B—C34B—C33B	121 (4)
O10—Na1—Dy1	42.6 (3)	C35B—C34B—H34B	119.6
O1—Na1—Dy1	42.9 (3)	C33B—C34B—H34B	119.6
O7—Na1—Dy1	42.8 (2)	C34B—C35B—C30B	120 (4)
O4—Na1—Dy1	42.9 (2)	C34B—C35B—H35B	120.2
O25—Na1—Mn1	41.9 (5)	C30B—C35B—H35B	120.2
O28—Na1—Mn1	95.2 (3)	C33B—O15B—H15B	109.5
O27—Na1—Mn1	166.1 (4)	C36—O16—Mn2	122.5 (14)
O26—Na1—Mn1	95.8 (4)	C36—O17—Dy1	138.2 (14)
O10—Na1—Mn1	51.0 (3)	O16—C36—O17	125 (2)
O1—Na1—Mn1	31.0 (3)	O16—C36—C37	119.3 (18)
O7—Na1—Mn1	101.3 (3)	O17—C36—C37	115.9 (19)
O4—Na1—Mn1	88.0 (3)	C38—C37—C42	124 (2)
Dy1—Na1—Mn1	62.77 (13)	C38—C37—C36	119 (2)
C7—O3—Mn2	129.8 (10)	C42—C37—C36	117 (2)
C8—O5—Mn2	111.3 (9)	C37—C38—C39	115 (2)
C14—O6—Mn3	128.9 (9)	C37—C38—H38	122.5
C15—O8—Mn3	111.3 (8)	C39—C38—H38	122.5
C21—O9—Mn4	130.4 (10)	C40—C39—C38	122 (3)
Na1—O25—Mn1	97.1 (6)	C40—C39—H39	118.9
Na1—O25—H25C	127.7	C38—C39—H39	118.9
Mn1—O25—H25C	105.4	O18—C40—C39	122 (3)
Na1—O25—H25D	136.0	O18—C40—C41	117 (3)
Mn1—O25—H25D	71.8	C39—C40—C41	120 (3)
H25C—O25—H25D	96.2	C42—C41—C40	121 (3)
Mn2—O26—Na1	98.6 (5)	C42—C41—H41	119.5
Mn2—O26—H26C	99 (10)	C40—C41—H41	119.5
Na1—O26—H26C	124 (5)	C41—C42—C37	117 (3)
Mn2—O26—H26D	94 (10)	C41—C42—H42	121.6
Na1—O26—H26D	125 (5)	C37—C42—H42	121.6
H26C—O26—H26D	106 (6)	C40—O18—H18O	109.5
Mn3—O27—Na1	96.9 (4)	C36B—O16B—Mn2	144 (6)
Mn3—O27—H27C	83 (10)	O16B—C36B—O17B	124 (2)
Na1—O27—H27C	128 (5)	O16B—C36B—C37B	118 (2)
Mn3—O27—H27D	111 (10)	O17B—C36B—C37B	118 (2)
Na1—O27—H27D	127 (5)	C38B—C37B—C42B	120.0
H27C—O27—H27D	101 (5)	C38B—C37B—C36B	117.5 (17)
Mn4—O28—Na1	96.2 (4)	C42B—C37B—C36B	120.3 (17)
Mn4—O28—H28C	93 (10)	C37B—C38B—C39B	120.0
Na1—O28—H28C	126 (5)	C37B—C38B—H38B	120.0
Mn4—O28—H28D	109 (10)	C39B—C38B—H38B	120.0
Na1—O28—H28D	126 (5)	C40B—C39B—C38B	120.0
H28C—O28—H28D	100 (5)	C40B—C39B—H39B	120.0
C1—N1—O1	110.6 (13)	C38B—C39B—H39B	120.0
C1B—N1—O1B	111 (2)	O18B—C40B—C39B	121.3 (16)
C1B—N1—Mn2	133.9 (19)	O18B—C40B—C41B	118.5 (16)
C1—N1—Mn2	130.1 (11)	C39B—C40B—C41B	120.0
O1—N1—Mn2	114.9 (8)	C42B—C41B—C40B	120.0
O1B—N1—Mn2	113.8 (14)	C42B—C41B—H41B	120.0
C15—N3—O7	112.6 (11)	C40B—C41B—H41B	120.0
C15—N3—O7B	112 (2)	C41B—C42B—C37B	120.0
C15—N3—Mn4	131.0 (10)	C41B—C42B—H42B	120.0
O7—N3—Mn4	114.5 (9)	C37B—C42B—H42B	120.0
O7B—N3—Mn4	116 (2)	C40B—O18B—H18B	109.5
C3—C2—C7	116.4 (15)	C43—O19—Mn3	128.8 (11)
C3—C2—C1	120.2 (15)	C43—O20—Dy1B	145.8 (12)
C7—C2—C1	123.3 (14)	O19—C43—O20	123.8 (14)
C3—C2—C1B	118 (2)	O19—C43—C44	118.2 (13)
C7—C2—C1B	125 (2)	O20—C43—C44	118.0 (13)
C4—C3—C2	123.8 (18)	C49—C44—C45	120.2 (9)
C4—C3—H3	118.1	C49—C44—C43	121.3 (10)
C2—C3—H3	118.1	C45—C44—C43	118.5 (10)
C3—C4—C5	119.6 (18)	C44—C45—C46	119.7 (9)
C3—C4—H4	120.2	C44—C45—H45	120.1
C5—C4—H4	120.2	C46—C45—H45	120.1
C4—C5—C6	120.2 (17)	C47—C46—C45	120.0 (9)
C4—C5—H5	119.9	C47—C46—H46	120.0
C6—C5—H5	119.9	C45—C46—H46	120.0
C7—C6—C5	119.6 (17)	O21—C47—C46	121.4 (10)
C7—C6—H6	120.2	O21—C47—C48	118.3 (10)
C5—C6—H6	120.2	C46—C47—C48	120.3 (9)
O3—C7—C6	116.9 (15)	C49—C48—C47	119.5 (9)
O3—C7—C2	122.5 (14)	C49—C48—H48	120.3
C6—C7—C2	120.6 (16)	C47—C48—H48	120.3
N2B—C8—O5	115 (5)	C44—C49—C48	120.2 (9)
O5—C8—N2	122.7 (15)	C44—C49—H49	119.9
N2B—C8—C9	126 (5)	C48—C49—H49	119.9
O5—C8—C9	119.0 (13)	C47—O21—H21O	109.5
N2—C8—C9	118.3 (15)	C50—O22—Mn4	118.0 (9)
C14—C9—C10	119.1 (15)	C50—O23—Dy1B	155.2 (11)
C14—C9—C8	123.5 (14)	O23—C50—O22	122.3 (14)
C10—C9—C8	117.2 (15)	O23—C50—C51	116.9 (13)
C9—C10—C11	122.0 (18)	O22—C50—C51	120.7 (12)
C9—C10—H10	119.0	C52—C51—C56	118.8 (12)
C11—C10—H10	119.0	C52—C51—C50	122.1 (12)
C12—C11—C10	118.7 (18)	C56—C51—C50	119.2 (13)
C12—C11—H11	120.7	C53—C52—C51	121.2 (13)
C10—C11—H11	120.7	C53—C52—H52	119.4
C11—C12—C13	120.2 (17)	C51—C52—H52	119.4
C11—C12—H12	119.9	C54—C53—C52	119.4 (13)
C13—C12—H12	119.9	C54—C53—H53	120.3
C12—C13—C14	120.5 (18)	C52—C53—H53	120.3
C12—C13—H13	119.7	O24—C54—C53	122.3 (13)
C14—C13—H13	119.7	O24—C54—C55	117.9 (13)
O6—C14—C9	123.9 (14)	C53—C54—C55	119.7 (13)
O6—C14—C13	116.9 (15)	C56—C55—C54	121.2 (14)
C9—C14—C13	119.2 (16)	C56—C55—H55	119.4
O8—C15—N3	121.1 (12)	C54—C55—H55	119.4
O8—C15—C16	119.2 (13)	C55—C56—C51	119.8 (14)
N3—C15—C16	119.7 (13)	C55—C56—H56	120.1
C17—C16—C21	120.8 (15)	C51—C56—H56	120.1
C17—C16—C15	118.1 (15)	C54—O24—H24O	109.5
C21—C16—C15	121.1 (14)	O29—C57—N5	130 (4)
C18—C17—C16	119.7 (18)	O29—C57—H57	115.2
C18—C17—H17	120.1	N5—C57—H57	115.2
C16—C17—H17	120.1	C57—N5—C58	115 (3)
C19—C18—C17	120.8 (17)	C57—N5—C59	124 (3)
C19—C18—H18	119.6	C58—N5—C59	121 (3)
C17—C18—H18	119.6	N5—C58—H58A	109.5
C18—C19—C20	121.8 (18)	N5—C58—H58B	109.5
C18—C19—H19	119.1	H58A—C58—H58B	109.5
C20—C19—H19	119.1	N5—C58—H58C	109.5
C19—C20—C21	121.3 (18)	H58A—C58—H58C	109.5
C19—C20—H20	119.4	H58B—C58—H58C	109.5
C21—C20—H20	119.4	N5—C59—H59A	109.5
O9—C21—C16	126.0 (14)	N5—C59—H59B	109.5
O9—C21—C20	118.5 (16)	H59A—C59—H59B	109.5
C16—C21—C20	115.5 (15)	N5—C59—H59C	109.5
O14—Dy1—O17	77.0 (5)	H59A—C59—H59C	109.5
O14—Dy1—O10	82.6 (5)	H59B—C59—H59C	109.5
O17—Dy1—O10	143.9 (5)	O29B—C57B—N5B	125 (4)
O14—Dy1—O7	144.2 (5)	O29B—C57B—H57B	117.6
O17—Dy1—O7	138.6 (5)	N5B—C57B—H57B	117.6
O10—Dy1—O7	65.0 (4)	C57B—N5B—C59B	127 (3)
O14—Dy1—O1	79.9 (5)	C57B—N5B—C58B	115 (3)
O17—Dy1—O1	82.2 (5)	C59B—N5B—C58B	118 (3)
O10—Dy1—O1	64.9 (4)	N5B—C58B—H58D	109.5
O7—Dy1—O1	98.4 (5)	N5B—C58B—H58E	109.5
O14—Dy1—O4	139.5 (5)	H58D—C58B—H58E	109.5
O17—Dy1—O4	79.4 (5)	N5B—C58B—H58F	109.5
O10—Dy1—O4	98.3 (4)	H58D—C58B—H58F	109.5
O7—Dy1—O4	64.3 (4)	H58E—C58B—H58F	109.5
O1—Dy1—O4	64.6 (4)	N5B—C59B—H59D	109.5
O14—Dy1—Na1	118.1 (5)	N5B—C59B—H59E	109.5
O17—Dy1—Na1	118.8 (4)	H59D—C59B—H59E	109.5
O10—Dy1—Na1	48.4 (3)	N5B—C59B—H59F	109.5
O7—Dy1—Na1	49.5 (4)	H59D—C59B—H59F	109.5
O1—Dy1—Na1	49.0 (3)	H59E—C59B—H59F	109.5
O4—Dy1—Na1	49.8 (3)	O30—C60—N6	127 (2)
N1—O1—Mn1	114.1 (9)	O30—C60—H60	116.7
N1—O1—Dy1	117.7 (9)	N6—C60—H60	116.7
Mn1—O1—Dy1	118.9 (6)	C60—N6—C61	121 (2)
N1—O1—Na1	110.6 (8)	C60—N6—C62	119 (2)
Mn1—O1—Na1	102.1 (6)	C61—N6—C62	120 (2)
Dy1—O1—Na1	88.2 (4)	N6—C61—H61A	109.5
C1—O2—Mn1	110.9 (11)	N6—C61—H61B	109.5
N2—O4—Mn2	113.3 (9)	H61A—C61—H61B	109.5
N2—O4—Dy1	120.9 (10)	N6—C61—H61C	109.5
Mn2—O4—Dy1	118.1 (6)	H61A—C61—H61C	109.5
N2—O4—Na1	105.8 (12)	H61B—C61—H61C	109.5
Mn2—O4—Na1	105.1 (6)	N6—C62—H62A	109.5
Dy1—O4—Na1	87.3 (4)	N6—C62—H62B	109.5
C8—N2—O4	110.7 (15)	H62A—C62—H62B	109.5
C8—N2—Mn3	132.9 (13)	N6—C62—H62C	109.5
O4—N2—Mn3	115.2 (10)	H62A—C62—H62C	109.5
O2—C1—N1	122.7 (16)	H62B—C62—H62C	109.5
O2—C1—C2	118.3 (15)	O31—C63—N7	127.3 (19)
N1—C1—C2	118.1 (15)	O31—C63—H63	116.3
C22—N4—O10	112.5 (12)	N7—C63—H63	116.3
C22—N4—Mn1	130.5 (11)	C63—N7—C64	119 (2)
O10—N4—Mn1	116.0 (9)	C63—N7—C65	122.3 (18)
N3—O7—Mn3	112.8 (8)	C64—N7—C65	119 (2)
N3—O7—Dy1	120.0 (9)	N7—C64—H64A	109.5
Mn3—O7—Dy1	119.7 (6)	N7—C64—H64B	109.5
N3—O7—Na1	106.1 (9)	H64A—C64—H64B	109.5
Mn3—O7—Na1	104.4 (7)	N7—C64—H64C	109.5
Dy1—O7—Na1	87.7 (5)	H64A—C64—H64C	109.5
N4—O10—Mn4	113.8 (9)	H64B—C64—H64C	109.5
N4—O10—Dy1	119.7 (9)	N7—C65—H65A	109.5
Mn4—O10—Dy1	118.4 (5)	N7—C65—H65B	109.5
N4—O10—Na1	105.5 (8)	H65A—C65—H65B	109.5
Mn4—O10—Na1	104.3 (5)	N7—C65—H65C	109.5
Dy1—O10—Na1	89.0 (4)	H65A—C65—H65C	109.5
C22—O11—Mn4	112.4 (11)	H65B—C65—H65C	109.5
O11—C22—N4	119.9 (15)	O32—C66—N8	126 (3)
O11—C22—C23	120.0 (16)	O32—C66—H66	117.2
N4—C22—C23	120.1 (14)	N8—C66—H66	117.2
C24—C23—C28	120.1 (14)	C66—N8—C67	119 (3)
C24—C23—C22	118.1 (15)	C66—N8—C68	115 (3)
C28—C23—C22	121.8 (14)	C67—N8—C68	125 (4)
C23—C24—C25	119.2 (17)	N8—C67—H67A	109.5
C23—C24—H24	120.4	N8—C67—H67B	109.5
C25—C24—H24	120.4	H67A—C67—H67B	109.5
C26—C25—C24	121.5 (17)	N8—C67—H67C	109.5
C26—C25—H25	119.3	H67A—C67—H67C	109.5
C24—C25—H25	119.3	H67B—C67—H67C	109.5
C25—C26—C27	120.4 (17)	N8—C68—H68A	109.5
C25—C26—H26	119.8	N8—C68—H68B	109.5
C27—C26—H26	119.8	H68A—C68—H68B	109.5
C26—C27—C28	120.9 (17)	N8—C68—H68C	109.5
C26—C27—H27	119.6	H68A—C68—H68C	109.5
C28—C27—H27	119.6	H68B—C68—H68C	109.5
O12—C28—C27	117.5 (15)	N8B—C66B—O32B	122 (4)
O12—C28—C23	124.4 (14)	N8B—C66B—H66B	119.2
C27—C28—C23	118.0 (15)	O32B—C66B—H66B	119.2
C28—O12—Mn1	128.7 (11)	C66B—N8B—C67B	118 (4)
O12—Mn1—O1	170.3 (7)	C66B—N8B—C68B	121 (3)
O12—Mn1—O2	97.5 (6)	C67B—N8B—C68B	120 (3)
O1—Mn1—O2	81.3 (5)	N8B—C67B—H67D	109.5
O12—Mn1—N4	90.9 (6)	N8B—C67B—H67E	109.5
O1—Mn1—N4	89.2 (5)	H67D—C67B—H67E	109.5
O2—Mn1—N4	168.5 (6)	N8B—C67B—H67F	109.5
O12—Mn1—O13	94.4 (7)	H67D—C67B—H67F	109.5
O1—Mn1—O13	95.2 (6)	H67E—C67B—H67F	109.5
O2—Mn1—O13	90.1 (6)	N8B—C68B—H68D	109.5
N4—Mn1—O13	97.1 (6)	N8B—C68B—H68E	109.5
O12—Mn1—O25	89.8 (6)	H68D—C68B—H68E	109.5
O1—Mn1—O25	80.5 (5)	N8B—C68B—H68F	109.5
O2—Mn1—O25	88.6 (6)	H68D—C68B—H68F	109.5
N4—Mn1—O25	83.5 (6)	H68E—C68B—H68F	109.5
O13—Mn1—O25	175.7 (6)		
			
O5—Mn2—O3—C7	178.5 (15)	C9—C8—N2B—O4B	171 (8)
N1—Mn2—O3—C7	7.7 (16)	O5—C8—N2B—Mn3	164 (7)
O16—Mn2—O3—C7	−91.4 (16)	C9—C8—N2B—Mn3	−23 (17)
O16B—Mn2—O3—C7	−88 (2)	Mn2—O4B—N2B—C8	10 (16)
O26—Mn2—O3—C7	92.8 (15)	Dy1B—O4B—N2B—C8	−154 (11)
Na1—Mn2—O3—C7	67.7 (16)	Mn2—O4B—N2B—Mn3	−157 (7)
N2—Mn3—O6—C14	6.5 (16)	Dy1B—O4B—N2B—Mn3	39 (12)
O8—Mn3—O6—C14	177.1 (13)	O1B—N1—C1B—O2B	−9 (5)
O19—Mn3—O6—C14	−89.5 (14)	Mn2—N1—C1B—O2B	−174 (4)
N2B—Mn3—O6—C14	4 (5)	O1B—N1—C1B—C2	172 (4)
O27—Mn3—O6—C14	91.2 (14)	Mn2—N1—C1B—C2	7 (6)
Na1—Mn3—O6—C14	66.1 (14)	Mn1B—O2B—C1B—N1	−7 (5)
O10—Mn4—O9—C21	96 (3)	Mn1B—O2B—C1B—C2	172 (4)
O11B—Mn4—O9—C21	−174 (5)	C3—C2—C1B—N1	−179 (3)
O11—Mn4—O9—C21	179.4 (14)	C7—C2—C1B—N1	−9 (5)
N3—Mn4—O9—C21	8.0 (13)	C3—C2—C1B—O2B	3 (5)
O22—Mn4—O9—C21	−91.3 (13)	C7—C2—C1B—O2B	172 (4)
O28—Mn4—O9—C21	93.2 (13)	C15—N3—O7B—Mn3	10 (6)
C22B—Mn4—O9—C21	−178 (3)	Mn4—N3—O7B—Mn3	−162 (3)
Na1—Mn4—O9—C21	68.6 (14)	C15—N3—O7B—Dy1B	−145 (4)
C7—C2—C3—C4	1 (3)	Mn4—N3—O7B—Dy1B	43 (6)
C1—C2—C3—C4	−175 (2)	C15—N3—O7B—Na1	113.3 (17)
C1B—C2—C3—C4	171 (3)	Mn4—N3—O7B—Na1	−59 (2)
C2—C3—C4—C5	−2 (3)	O8—Mn3—O7B—N3	−9 (4)
C3—C4—C5—C6	2 (3)	O19—Mn3—O7B—N3	−102 (4)
C4—C5—C6—C7	0 (3)	N2B—Mn3—O7B—N3	164 (6)
Mn2—O3—C7—C6	168.7 (13)	O27—Mn3—O7B—N3	77 (4)
Mn2—O3—C7—C2	−12 (3)	Na1—Mn3—O7B—N3	98 (5)
C5—C6—C7—O3	177.8 (17)	O8—Mn3—O7B—Dy1B	144 (5)
C5—C6—C7—C2	−1 (3)	O19—Mn3—O7B—Dy1B	51 (5)
C3—C2—C7—O3	−178.1 (17)	N2B—Mn3—O7B—Dy1B	−43 (6)
C1—C2—C7—O3	−3 (3)	O27—Mn3—O7B—Dy1B	−131 (5)
C1B—C2—C7—O3	12 (3)	Na1—Mn3—O7B—Dy1B	−109 (6)
C3—C2—C7—C6	1 (3)	O8—Mn3—O7B—Na1	−107.1 (17)
C1—C2—C7—C6	176.0 (18)	O19—Mn3—O7B—Na1	159.6 (18)
C1B—C2—C7—C6	−169 (3)	N2B—Mn3—O7B—Na1	66 (5)
Mn2—O5—C8—N2B	−8 (9)	O27—Mn3—O7B—Na1	−21.6 (16)
Mn2—O5—C8—N2	−1 (2)	C22B—N4B—O10B—Mn4	−1 (9)
Mn2—O5—C8—C9	179.4 (11)	Mn1B—N4B—O10B—Mn4	−158 (4)
N2B—C8—C9—C14	13 (10)	C22B—N4B—O10B—Dy1B	−155 (6)
O5—C8—C9—C14	−175.1 (15)	Mn1B—N4B—O10B—Dy1B	48 (7)
N2—C8—C9—C14	5 (3)	C22B—N4B—O10B—Na1	98 (7)
N2B—C8—C9—C10	−162 (10)	Mn1B—N4B—O10B—Na1	−59 (5)
O5—C8—C9—C10	10 (2)	Mn4—O11B—C22B—N4B	−14 (16)
N2—C8—C9—C10	−170.0 (19)	Mn4—O11B—C22B—C23B	170 (6)
C14—C9—C10—C11	1 (3)	O10B—N4B—C22B—O11B	10 (15)
C8—C9—C10—C11	176.7 (17)	Mn1B—N4B—C22B—O11B	164 (10)
C9—C10—C11—C12	−4 (3)	O10B—N4B—C22B—C23B	−174 (5)
C10—C11—C12—C13	5 (3)	Mn1B—N4B—C22B—C23B	−21 (11)
C11—C12—C13—C14	−4 (3)	O10B—N4B—C22B—Mn4	1 (6)
Mn3—O6—C14—C9	−14 (2)	Mn1B—N4B—C22B—Mn4	154 (6)
Mn3—O6—C14—C13	168.4 (12)	O11B—C22B—C23B—C24B	10 (10)
C10—C9—C14—O6	−177.1 (16)	N4B—C22B—C23B—C24B	−166 (7)
C8—C9—C14—O6	8 (3)	Mn4—C22B—C23B—C24B	25 (10)
C10—C9—C14—C13	0 (2)	O11B—C22B—C23B—C28B	−170 (10)
C8—C9—C14—C13	−174.5 (16)	N4B—C22B—C23B—C28B	14 (7)
C12—C13—C14—O6	178.4 (17)	Mn4—C22B—C23B—C28B	−155 (10)
C12—C13—C14—C9	1 (3)	C28B—C23B—C24B—C25B	0.0
Mn3—O8—C15—N3	−3.9 (17)	C22B—C23B—C24B—C25B	−180.0 (9)
Mn3—O8—C15—C16	176.8 (10)	C23B—C24B—C25B—C26B	0.0
O7—N3—C15—O8	3.6 (19)	C24B—C25B—C26B—C27B	0.0
O7B—N3—C15—O8	−4 (4)	C25B—C26B—C27B—C28B	0.0
Mn4—N3—C15—O8	166.7 (10)	C26B—C27B—C28B—O12B	180 (3)
O7—N3—C15—C16	−177.1 (13)	C26B—C27B—C28B—C23B	0.0
O7B—N3—C15—C16	176 (4)	C24B—C23B—C28B—O12B	−180 (3)
Mn4—N3—C15—C16	−14 (2)	C22B—C23B—C28B—O12B	0 (3)
O8—C15—C16—C17	10 (2)	C24B—C23B—C28B—C27B	0.0
N3—C15—C16—C17	−168.9 (14)	C22B—C23B—C28B—C27B	180.0 (10)
O8—C15—C16—C21	−171.0 (13)	C27B—C28B—O12B—Mn1B	173 (5)
N3—C15—C16—C21	10 (2)	C23B—C28B—O12B—Mn1B	−7 (6)
C21—C16—C17—C18	0 (3)	C28B—O12B—Mn1B—O1B	93 (12)
C15—C16—C17—C18	178.9 (16)	C28B—O12B—Mn1B—O2B	173 (5)
C16—C17—C18—C19	−1 (3)	C28B—O12B—Mn1B—N4B	2 (5)
C17—C18—C19—C20	2 (4)	C28B—O12B—Mn1B—O13B	−95 (5)
C18—C19—C20—C21	−1 (3)	Mn1B—O13B—C29B—O14B	−16 (15)
Mn4—O9—C21—C16	−13 (2)	Mn1B—O13B—C29B—C30B	152 (8)
Mn4—O9—C21—C20	168.7 (12)	Dy1B—O14B—C29B—O13B	58 (15)
C17—C16—C21—O9	−178.1 (15)	Dy1B—O14B—C29B—C30B	−111 (9)
C15—C16—C21—O9	3 (2)	O13B—C29B—C30B—C31B	28 (17)
C17—C16—C21—C20	0 (2)	O14B—C29B—C30B—C31B	−163 (10)
C15—C16—C21—C20	−178.3 (15)	O13B—C29B—C30B—C35B	−176 (11)
C19—C20—C21—O9	178.7 (17)	O14B—C29B—C30B—C35B	−6 (15)
C19—C20—C21—C16	0 (3)	C35B—C30B—C31B—C32B	9 (20)
C1—N1—O1—Mn1	−6.2 (17)	C29B—C30B—C31B—C32B	165 (14)
Mn2—N1—O1—Mn1	−164.9 (7)	C30B—C31B—C32B—C33B	11 (26)
C1—N1—O1—Dy1	−152.6 (13)	C31B—C32B—C33B—O15B	179 (15)
Mn2—N1—O1—Dy1	48.7 (12)	C31B—C32B—C33B—C34B	−20 (25)
C1—N1—O1—Na1	108.2 (14)	O15B—C33B—C34B—C35B	171 (12)
Mn2—N1—O1—Na1	−50.5 (11)	C32B—C33B—C34B—C35B	9 (20)
O5—C8—N2—O4	1 (3)	C33B—C34B—C35B—C30B	10 (20)
C9—C8—N2—O4	−179.4 (15)	C31B—C30B—C35B—C34B	−19 (19)
O5—C8—N2—Mn3	167.3 (18)	C29B—C30B—C35B—C34B	−176 (12)
C9—C8—N2—Mn3	−13 (3)	Mn2—O16—C36—O17	−12 (3)
Mn2—O4—N2—C8	0 (2)	Mn2—O16—C36—C37	171.8 (16)
Dy1—O4—N2—C8	−149.3 (15)	Dy1—O17—C36—O16	57 (4)
Na1—O4—N2—C8	114.2 (18)	Dy1—O17—C36—C37	−127 (2)
Mn2—O4—N2—Mn3	−169.5 (11)	O16—C36—C37—C38	9 (4)
Dy1—O4—N2—Mn3	42 (2)	O17—C36—C37—C38	−167 (3)
Na1—O4—N2—Mn3	−54.9 (17)	O16—C36—C37—C42	−172 (3)
Mn1—O2—C1—N1	−7 (3)	O17—C36—C37—C42	12 (3)
Mn1—O2—C1—C2	−175.4 (14)	C42—C37—C38—C39	1 (4)
O1—N1—C1—O2	9 (3)	C36—C37—C38—C39	−180 (2)
Mn2—N1—C1—O2	163.3 (15)	C37—C38—C39—C40	1 (4)
O1—N1—C1—C2	177.3 (15)	C38—C39—C40—O18	180 (3)
Mn2—N1—C1—C2	−28 (3)	C38—C39—C40—C41	2 (5)
C3—C2—C1—O2	7 (3)	O18—C40—C41—C42	176 (3)
C7—C2—C1—O2	−168.5 (18)	C39—C40—C41—C42	−6 (5)
C3—C2—C1—N1	−162.4 (18)	C40—C41—C42—C37	8 (5)
C7—C2—C1—N1	23 (3)	C38—C37—C42—C41	−6 (5)
C15—N3—O7—Mn3	−1.5 (16)	C36—C37—C42—C41	175 (3)
Mn4—N3—O7—Mn3	−167.5 (7)	Mn2—O16B—C36B—O17B	39 (23)
C15—N3—O7—Dy1	−151.0 (10)	Mn2—O16B—C36B—C37B	−148 (7)
Mn4—N3—O7—Dy1	43.0 (14)	O16B—C36B—C37B—C38B	76 (11)
C15—N3—O7—Na1	112.2 (10)	O17B—C36B—C37B—C38B	−110 (11)
Mn4—N3—O7—Na1	−53.9 (9)	O16B—C36B—C37B—C42B	−121 (10)
C22—N4—O10—Mn4	2.8 (17)	O17B—C36B—C37B—C42B	53 (13)
Mn1—N4—O10—Mn4	−167.4 (7)	C42B—C37B—C38B—C39B	0.0
C22—N4—O10—Dy1	−145.6 (12)	C36B—C37B—C38B—C39B	163 (6)
Mn1—N4—O10—Dy1	44.2 (13)	C37B—C38B—C39B—C40B	0.0
C22—N4—O10—Na1	116.5 (13)	C38B—C39B—C40B—O18B	−175 (13)
Mn1—N4—O10—Na1	−53.6 (11)	C38B—C39B—C40B—C41B	0.0
Mn4—O11—C22—N4	0 (2)	O18B—C40B—C41B—C42B	175 (12)
Mn4—O11—C22—C23	179.2 (13)	C39B—C40B—C41B—C42B	0.0
O10—N4—C22—O11	−2 (2)	C40B—C41B—C42B—C37B	0.0
Mn1—N4—C22—O11	166.4 (15)	C38B—C37B—C42B—C41B	0.0
O10—N4—C22—C23	179.1 (14)	C36B—C37B—C42B—C41B	−163 (6)
Mn1—N4—C22—C23	−13 (3)	O23—Dy1B—O20—C43	51 (2)
O11—C22—C23—C24	14 (3)	O14B—Dy1B—O20—C43	125 (3)
N4—C22—C23—C24	−166.7 (17)	O7B—Dy1B—O20—C43	−27 (3)
O11—C22—C23—C28	−165.5 (19)	O4B—Dy1B—O20—C43	−91 (3)
N4—C22—C23—C28	13 (3)	O10B—Dy1B—O20—C43	1 (4)
C28—C23—C24—C25	0 (3)	O1B—Dy1B—O20—C43	−114 (3)
C22—C23—C24—C25	−179.4 (17)	Na1—Dy1B—O20—C43	−53 (2)
C23—C24—C25—C26	1 (3)	Mn3—O19—C43—O20	−2 (2)
C24—C25—C26—C27	−1 (3)	Mn3—O19—C43—C44	179.4 (10)
C25—C26—C27—C28	0 (3)	Dy1B—O20—C43—O19	47 (3)
C26—C27—C28—O12	177 (2)	Dy1B—O20—C43—C44	−134.3 (18)
C26—C27—C28—C23	1 (3)	O19—C43—C44—C49	−167.4 (16)
C24—C23—C28—O12	−177.5 (19)	O20—C43—C44—C49	14 (2)
C22—C23—C28—O12	2 (3)	O19—C43—C44—C45	12 (2)
C24—C23—C28—C27	−1 (3)	O20—C43—C44—C45	−166.4 (15)
C22—C23—C28—C27	178.3 (18)	C49—C44—C45—C46	−1 (2)
C27—C28—O12—Mn1	164.1 (15)	C43—C44—C45—C46	179.8 (14)
C23—C28—O12—Mn1	−20 (3)	C44—C45—C46—C47	0 (2)
C28—O12—Mn1—O2	−171.0 (17)	C45—C46—C47—O21	−179.9 (14)
C28—O12—Mn1—N4	16.8 (17)	C45—C46—C47—C48	−1 (2)
C28—O12—Mn1—O13	−80.4 (17)	O21—C47—C48—C49	−179.4 (16)
C28—O12—Mn1—O25	100.4 (17)	C46—C47—C48—C49	2 (3)
C28—O12—Mn1—Na1	77.5 (18)	C45—C44—C49—C48	1 (3)
Mn1—O13—C29—O14	−14 (3)	C43—C44—C49—C48	−179.2 (16)
Mn1—O13—C29—C30	166.8 (14)	C47—C48—C49—C44	−2 (3)
Dy1—O14—C29—O13	55 (3)	Dy1B—O23—C50—O22	73 (3)
Dy1—O14—C29—C30	−126 (2)	Dy1B—O23—C50—C51	−108 (2)
O13—C29—C30—C35	−168 (2)	Mn4—O22—C50—O23	−2.8 (19)
O14—C29—C30—C35	13 (3)	Mn4—O22—C50—C51	178.3 (10)
O13—C29—C30—C31	13 (3)	O23—C50—C51—C52	−166.4 (14)
O14—C29—C30—C31	−165.6 (19)	O22—C50—C51—C52	13 (2)
C35—C30—C31—C32	0 (3)	O23—C50—C51—C56	13 (2)
C29—C30—C31—C32	178 (2)	O22—C50—C51—C56	−168.0 (14)
C30—C31—C32—C33	1 (3)	C56—C51—C52—C53	0 (2)
C31—C32—C33—O15	−173 (3)	C50—C51—C52—C53	179.2 (14)
C31—C32—C33—C34	0 (4)	C51—C52—C53—C54	0 (2)
O15—C33—C34—C35	172 (3)	C52—C53—C54—O24	−176.4 (13)
C32—C33—C34—C35	−2 (5)	C52—C53—C54—C55	1 (2)
C31—C30—C35—C34	−1 (4)	O24—C54—C55—C56	176.3 (15)
C29—C30—C35—C34	−179 (2)	C53—C54—C55—C56	−1 (3)
C33—C34—C35—C30	2 (4)	C54—C55—C56—C51	1 (3)
C1B—N1—O1B—Mn1B	20 (5)	C52—C51—C56—C55	0 (2)
Mn2—N1—O1B—Mn1B	−171.3 (19)	C50—C51—C56—C55	−179.4 (15)
C1B—N1—O1B—Dy1B	−145 (4)	O29—C57—N5—C58	8 (6)
Mn2—N1—O1B—Dy1B	24 (5)	O29—C57—N5—C59	−170 (4)
O5—Mn2—O4B—N2B	−10 (7)	O29B—C57B—N5B—C59B	−162 (8)
N1—Mn2—O4B—N2B	160 (7)	O29B—C57B—N5B—C58B	21 (10)
O16B—Mn2—O4B—N2B	−104 (8)	O30—C60—N6—C61	−3 (3)
O26—Mn2—O4B—N2B	75 (7)	O30—C60—N6—C62	−176 (2)
Na1—Mn2—O4B—N2B	99 (8)	O31—C63—N7—C64	−1 (3)
O5—Mn2—O4B—Dy1B	153 (5)	O31—C63—N7—C65	176.2 (19)
N1—Mn2—O4B—Dy1B	−37 (5)	O32—C66—N8—C67	−2 (7)
O16B—Mn2—O4B—Dy1B	59 (5)	O32—C66—N8—C68	−176 (4)
O26—Mn2—O4B—Dy1B	−122 (5)	O32B—C66B—N8B—C67B	−7 (8)
Na1—Mn2—O4B—Dy1B	−98 (5)	O32B—C66B—N8B—C68B	−176 (4)
O5—C8—N2B—O4B	−2 (17)		

### Tetra-µ-aqua-tetrakis{2-[azanidylene(oxido)methyl]phenolato}tetrakis(µ2-3-hydroxybenzoato)dysprosium(III)tetramanganese(III)sodium(I) N,N-dimethylformamide tetrasolvate (2) Hydrogen-bond geometry (Å, º)

**Table:**

*D*—H···*A*	*D*—H	H···*A*	*D*···*A*	*D*—H···*A*
O25—H25*C*···O29	0.92	2.00	2.74 (3)	137
O25—H25*D*···O12	0.87	2.41	3.06 (2)	132
O26—H26*C*···O30	0.85 (4)	2.04 (9)	2.74 (2)	138 (10)
O26—H26*D*···O29	0.84 (4)	2.03 (11)	2.70 (4)	136 (11)
O27—H27*C*···O30	0.87 (4)	2.12 (14)	2.730 (19)	127 (14)
O27—H27*D*···O31	0.87 (4)	2.09 (7)	2.798 (18)	138 (6)
O28—H28*C*···O31	0.88 (4)	2.07 (10)	2.776 (17)	137 (10)
O28—H28*D*···O32	0.88 (4)	1.94 (7)	2.68 (3)	142 (6)
C32—H32···O3^i^	0.95	2.66	3.35 (2)	131
O15—H15*O*···O3^i^	0.84	1.93	2.77 (2)	175
C46—H46···O9^ii^	0.95	2.24	3.168 (15)	165
O21—H21*O*···O22^ii^	0.84	2.01	2.794 (16)	155
O24—H24*O*···O6^iii^	0.84	2.02	2.815 (16)	158

Symmetry codes: (i) *x*−1/2, −*y*+1, *z*+1/2; (ii) *x*−1/2, −*y*+2, *z*−1/2; (iii) *x*−1/2, −*y*+2, *z*+1/2.

### Tetra-µ-aqua-tetrakis{2-[azanidylene(oxido)methyl]phenolato}tetrakis(µ2-3-hydroxybenzoato)dysprosium(III)tetramanganese(III)sodium(I) N,N-dimethylacetamide decasolvate (1) Crystal data

**Table:**

[DyMn_4_Na(C_7_H_5_O_3_)_4_(C_7_H_4_NO_2_)_4_(H_2_O)_4_]·10C_4_H_9_NO	*D*_x_ = 1.488 Mg m^−^^3^
*M**_r_* = 2497.41	Mo *K*α radiation, λ = 0.71073 Å
Tetragonal, *P*4/*n*	Cell parameters from 9707 reflections
*a* = 19.9869 (9) Å	θ = 2.7–32.8°
*c* = 13.9570 (11) Å	µ = 1.19 mm^−^^1^
*V* = 5575.5 (7) Å^3^	*T* = 150 K
*Z* = 2	Block, brown
*F*(000) = 2578	0.25 × 0.23 × 0.15 mm

### Tetra-µ-aqua-tetrakis{2-[azanidylene(oxido)methyl]phenolato}tetrakis(µ2-3-hydroxybenzoato)dysprosium(III)tetramanganese(III)sodium(I) N,N-dimethylacetamide decasolvate (1) Data collection

**Table:**

Bruker AXS D8 Quest CMOS diffractometer	7967 independent reflections
Radiation source: fine focus sealed tube X-ray source	6605 reflections with *I* > 2σ(*I*)
Triumph curved graphite crystal monochromator	*R*_int_ = 0.042
Detector resolution: 10.4167 pixels mm^-1^	θ_max_ = 30.5°, θ_min_ = 2.5°
ω and phi scans	*h* = −25→24
Absorption correction: multi-scan (SADABS; Krause *et al.*, 2015)	*k* = −27→24
*T*_min_ = 0.024, *T*_max_ = 0.055	*l* = −17→19
58638 measured reflections	

### Tetra-µ-aqua-tetrakis{2-[azanidylene(oxido)methyl]phenolato}tetrakis(µ2-3-hydroxybenzoato)dysprosium(III)tetramanganese(III)sodium(I) N,N-dimethylacetamide decasolvate (1) Refinement

**Table:**

Refinement on *F*^2^	1550 restraints
Least-squares matrix: full	Hydrogen site location: mixed
*R*[*F*^2^ > 2σ(*F*^2^)] = 0.051	H-atom parameters constrained
*wR*(*F*^2^) = 0.151	*w* = 1/[σ^2^(*F*_o_^2^) + (0.0823*P*)^2^ + 8.7362*P*] where *P* = (*F*_o_^2^ + 2*F*_c_^2^)/3
*S* = 1.04	(Δ/σ)_max_ = 0.001
7967 reflections	Δρ_max_ = 2.49 e Å^−^^3^
761 parameters	Δρ_min_ = −0.91 e Å^−^^3^

### Tetra-µ-aqua-tetrakis{2-[azanidylene(oxido)methyl]phenolato}tetrakis(µ2-3-hydroxybenzoato)dysprosium(III)tetramanganese(III)sodium(I) N,N-dimethylacetamide decasolvate (1) Special details

**Table:**

Geometry. All esds (except the esd in the dihedral angle between two l.s. planes) are estimated using the full covariance matrix. The cell esds are taken into account individually in the estimation of esds in distances, angles and torsion angles; correlations between esds in cell parameters are only used when they are defined by crystal symmetry. An approximate (isotropic) treatment of cell esds is used for estimating esds involving l.s. planes.
Refinement. Whole molecule disorder is observed for the main molecule, excluding only the Dy and Na ions. Equivalent disordered organic moieties were restrained to have similar geometries, and Uij components of ADPs for all disordered atoms closer to each other than 2.0 Angstrom were restrained to be similar. Subject to these conditions the occupancy ratio refined to 0.8018 (14) to 0.1982 (14). Three DMA molecules were refined as disordered. Two in general positions by an approximate 180 degree rotation. The third is in addition also disordered by an exact 180 degree rotation from a two fold axis that bisects it. All DMA moieties were restrained to have similar geometries SAME command of Shelxl). All N-CH3 bond lengths were restrained to be similar to each other, and all 1,3 distances of the C-N-CH3 angles were also restrained to be similar. Uij components of ADPs for all DMA atoms closer to each other than 2.0 Angstrom were restrained to be similar, and the atoms of the four fold disordered molecule were restrained to be close to isotropic. The least occupied DMA molecule (the minor component disordered by two fold symmetry) was restrained to be close to planar. Subject to these conditions the occupancy ratios refined to 0.496 (8) to 0.504 (8), 0.608 (9) to 0.392 (9), and two times 0.275 (7) to two times 0.225 (7). Alcohol H atoms were initially allowed to rotate and Water H atom positions were initially refined while a damping factor was applied and O-H and H···H distances were restrained to 0.84 (2) and 1.36 (2) Angstrom, respectively. Some water H atom positions were further restrained based on hydrogen bonding considerations. In the final refinement cycles these H atoms were set to ride on their carrier oxygen atoms and the damping factor was removed.

### Tetra-µ-aqua-tetrakis{2-[azanidylene(oxido)methyl]phenolato}tetrakis(µ2-3-hydroxybenzoato)dysprosium(III)tetramanganese(III)sodium(I) N,N-dimethylacetamide decasolvate (1) Fractional atomic coordinates and isotropic or equivalent isotropic displacement parameters (Å^2^)

**Table:**

	*x*	*y*	*z*	*U*_iso_*/*U*_eq_	Occ. (<1)
Mn1	0.84667 (3)	0.88157 (3)	0.49946 (4)	0.02920 (15)	0.8018 (14)
O1	0.76087 (12)	0.84232 (12)	0.47528 (18)	0.0294 (5)	0.8018 (14)
N1	0.7083 (4)	0.8872 (3)	0.4910 (14)	0.0291 (9)	0.8018 (14)
O2	0.79161 (13)	0.96071 (14)	0.5213 (2)	0.0333 (5)	0.8018 (14)
C1	0.72846 (17)	0.94758 (17)	0.5145 (2)	0.0300 (6)	0.8018 (14)
C2	0.67924 (19)	1.0008 (2)	0.5321 (4)	0.0315 (8)	0.8018 (14)
C3	0.7020 (2)	1.0669 (2)	0.5343 (4)	0.0453 (9)	0.8018 (14)
H3	0.748483	1.075493	0.526983	0.054*	0.8018 (14)
C4	0.6587 (3)	1.1202 (3)	0.5468 (6)	0.0559 (13)	0.8018 (14)
H4	0.674759	1.164916	0.545125	0.067*	0.8018 (14)
C5	0.5909 (2)	1.1072 (3)	0.5619 (5)	0.0480 (12)	0.8018 (14)
H5	0.560912	1.143107	0.573807	0.058*	0.8018 (14)
C6	0.5671 (2)	1.04252 (19)	0.5595 (3)	0.0385 (8)	0.8018 (14)
H6	0.520704	1.034486	0.568776	0.046*	0.8018 (14)
C7	0.61041 (18)	0.98822 (18)	0.5437 (3)	0.0326 (7)	0.8018 (14)
O3	0.58331 (14)	0.92701 (16)	0.5428 (2)	0.0378 (6)	0.8018 (14)
O4	0.86173 (16)	0.91072 (15)	0.3509 (2)	0.0427 (6)	0.8018 (14)
O5	0.82694 (16)	0.81397 (16)	0.2889 (2)	0.0477 (7)	0.8018 (14)
C8	0.8473 (2)	0.8741 (2)	0.2817 (3)	0.0448 (8)	0.8018 (14)
C9	0.8557 (2)	0.9003 (2)	0.1808 (3)	0.0501 (9)	0.8018 (14)
C10	0.8788 (3)	0.9631 (3)	0.1655 (4)	0.0600 (12)	0.8018 (14)
H10	0.889378	0.991320	0.218185	0.072*	0.8018 (14)
C11	0.8870 (4)	0.9860 (3)	0.0718 (4)	0.0699 (15)	0.8018 (14)
C12	0.8687 (4)	0.9467 (3)	−0.0058 (4)	0.0733 (15)	0.8018 (14)
H12	0.873706	0.963218	−0.069169	0.088*	0.8018 (14)
C13	0.8433 (5)	0.8838 (4)	0.0096 (4)	0.0833 (17)	0.8018 (14)
H13	0.829448	0.856841	−0.042877	0.100*	0.8018 (14)
C14	0.8382 (4)	0.8597 (3)	0.1036 (4)	0.0731 (14)	0.8018 (14)
H14	0.822630	0.815505	0.114732	0.088*	0.8018 (14)
O6	0.9105 (4)	1.0486 (3)	0.0591 (5)	0.107 (3)	0.8018 (14)
H6A1	0.930968	1.051182	0.006681	0.161*	0.8018 (14)
O7	0.81729 (18)	0.8449 (2)	0.6615 (3)	0.0385 (7)	0.8018 (14)
H7A	0.790436	0.872875	0.681729	0.058*	0.8018 (14)
H7B	0.846055	0.835775	0.700792	0.058*	0.8018 (14)
Mn1B	0.80037 (11)	0.90547 (11)	0.50499 (15)	0.0296 (6)	0.1982 (14)
O1B	0.6868 (5)	0.8187 (5)	0.4794 (7)	0.0309 (17)	0.1982 (14)
N1B	0.7022 (16)	0.8864 (12)	0.494 (6)	0.029 (3)	0.1982 (14)
O2B	0.5921 (5)	0.8978 (6)	0.5262 (9)	0.033 (2)	0.1982 (14)
C1B	0.6507 (6)	0.9230 (6)	0.5201 (11)	0.0326 (17)	0.1982 (14)
C2B	0.6592 (7)	0.9958 (7)	0.5358 (19)	0.032 (2)	0.1982 (14)
C3B	0.6023 (8)	1.0354 (7)	0.5408 (13)	0.040 (2)	0.1982 (14)
H3B	0.559206	1.015307	0.539614	0.048*	0.1982 (14)
C4B	0.6085 (10)	1.1051 (10)	0.548 (2)	0.048 (3)	0.1982 (14)
H4B	0.570364	1.132836	0.539250	0.057*	0.1982 (14)
C5B	0.6702 (10)	1.1335 (10)	0.567 (2)	0.050 (3)	0.1982 (14)
H5B	0.673353	1.178950	0.586179	0.059*	0.1982 (14)
C6B	0.7271 (8)	1.0951 (7)	0.5574 (15)	0.045 (2)	0.1982 (14)
H6B	0.769744	1.116048	0.559490	0.054*	0.1982 (14)
C7B	0.7232 (6)	1.0252 (6)	0.5449 (12)	0.0356 (19)	0.1982 (14)
O3B	0.7818 (5)	0.9910 (6)	0.5478 (9)	0.0333 (18)	0.1982 (14)
O4B	0.8128 (6)	0.9351 (6)	0.3564 (7)	0.041 (2)	0.1982 (14)
O5B	0.7561 (6)	0.8492 (6)	0.2932 (9)	0.048 (2)	0.1982 (14)
C8B	0.7903 (9)	0.9028 (8)	0.2876 (9)	0.045 (2)	0.1982 (14)
C9B	0.8068 (11)	0.9253 (9)	0.1869 (10)	0.058 (2)	0.1982 (14)
C10B	0.8423 (14)	0.9834 (11)	0.1727 (13)	0.063 (3)	0.1982 (14)
H10B	0.854922	1.010086	0.226086	0.075*	0.1982 (14)
C11B	0.8600 (16)	1.0033 (12)	0.0797 (13)	0.072 (3)	0.1982 (14)
C12B	0.8321 (17)	0.9692 (13)	0.0022 (14)	0.078 (3)	0.1982 (14)
H12B	0.837071	0.986818	−0.060584	0.094*	0.1982 (14)
C13B	0.7975 (17)	0.9106 (13)	0.0155 (13)	0.076 (3)	0.1982 (14)
H13B	0.781114	0.886496	−0.038287	0.091*	0.1982 (14)
C14B	0.7863 (14)	0.8865 (11)	0.1081 (11)	0.068 (3)	0.1982 (14)
H14B	0.765235	0.844480	0.117654	0.081*	0.1982 (14)
O6B	0.8818 (16)	1.0672 (11)	0.072 (2)	0.088 (6)	0.1982 (14)
H6B1	0.893403	1.072920	0.014649	0.133*	0.1982 (14)
O7B	0.7925 (6)	0.8580 (8)	0.6681 (10)	0.033 (2)	0.1982 (14)
H7C	0.785662	0.887227	0.710119	0.049*	0.1982 (14)
H7D	0.829921	0.840159	0.679782	0.049*	0.1982 (14)
O8	0.7267 (4)	0.9190 (4)	0.7513 (9)	0.0471 (16)	0.496 (8)
C15	0.7303 (5)	0.9804 (4)	0.7656 (7)	0.071 (2)	0.496 (8)
C16	0.7934 (6)	1.0243 (8)	0.7470 (11)	0.069 (3)	0.496 (8)
H16A	0.830606	0.995602	0.726844	0.104*	0.496 (8)
H16B	0.805643	1.047829	0.806064	0.104*	0.496 (8)
H16C	0.783652	1.056920	0.696582	0.104*	0.496 (8)
N2	0.6781 (4)	1.0137 (4)	0.7929 (6)	0.0651 (19)	0.496 (8)
C17	0.6152 (6)	0.9691 (8)	0.8040 (12)	0.064 (3)	0.496 (8)
H17A	0.587299	0.973098	0.746621	0.096*	0.496 (8)
H17B	0.589671	0.983588	0.860243	0.096*	0.496 (8)
H17C	0.628959	0.922380	0.812295	0.096*	0.496 (8)
C18	0.6987 (10)	1.0894 (6)	0.8151 (17)	0.103 (5)	0.496 (8)
H18A	0.712974	1.111290	0.755691	0.154*	0.496 (8)
H18B	0.735553	1.090030	0.861402	0.154*	0.496 (8)
H18C	0.660178	1.113333	0.841844	0.154*	0.496 (8)
O8B	0.7021 (7)	0.9110 (5)	0.7572 (10)	0.088 (3)	0.504 (8)
C15B	0.6841 (5)	0.9691 (4)	0.7775 (6)	0.069 (2)	0.504 (8)
C16B	0.6074 (7)	0.9806 (10)	0.7853 (14)	0.086 (4)	0.504 (8)
H16D	0.594807	1.019693	0.746971	0.129*	0.504 (8)
H16E	0.595329	0.988266	0.852428	0.129*	0.504 (8)
H16F	0.583732	0.941032	0.761345	0.129*	0.504 (8)
N2B	0.7244 (5)	1.0225 (4)	0.7873 (7)	0.078 (2)	0.504 (8)
C17B	0.7972 (6)	1.0092 (9)	0.7693 (15)	0.104 (5)	0.504 (8)
H17D	0.807718	1.018857	0.702167	0.155*	0.504 (8)
H17E	0.807091	0.962171	0.783142	0.155*	0.504 (8)
H17F	0.824296	1.037917	0.810972	0.155*	0.504 (8)
C18B	0.6739 (9)	1.0864 (5)	0.7973 (14)	0.087 (4)	0.504 (8)
H18D	0.672813	1.111050	0.736713	0.131*	0.504 (8)
H18E	0.689809	1.115905	0.848546	0.131*	0.504 (8)
H18F	0.628823	1.070413	0.812822	0.131*	0.504 (8)
O9	0.9251 (8)	1.0845 (6)	0.8857 (9)	0.090 (4)	0.608 (9)
C19	0.9589 (5)	1.1335 (4)	0.8605 (5)	0.0737 (19)	0.608 (9)
C20	0.9736 (9)	1.1944 (8)	0.9272 (10)	0.096 (4)	0.608 (9)
H20A	1.019835	1.209408	0.917513	0.145*	0.608 (9)
H20B	0.967498	1.181067	0.994213	0.145*	0.608 (9)
H20C	0.942838	1.231018	0.911850	0.145*	0.608 (9)
N3	0.9712 (4)	1.1468 (4)	0.7693 (5)	0.0734 (17)	0.608 (9)
C21	0.9586 (13)	1.0828 (8)	0.7072 (10)	0.102 (4)	0.608 (9)
H21A	0.917348	1.088456	0.670116	0.153*	0.608 (9)
H21B	0.954167	1.043764	0.749307	0.153*	0.608 (9)
H21C	0.996310	1.076029	0.663456	0.153*	0.608 (9)
C22	1.0080 (10)	1.2133 (7)	0.7482 (10)	0.084 (4)	0.608 (9)
H22A	1.044163	1.219537	0.794745	0.126*	0.608 (9)
H22B	0.976308	1.250613	0.753216	0.126*	0.608 (9)
H22C	1.026709	1.211994	0.683363	0.126*	0.608 (9)
O9B	0.9394 (13)	1.0698 (9)	0.8677 (14)	0.082 (4)	0.392 (9)
C19B	0.9602 (7)	1.1050 (6)	0.8021 (8)	0.075 (2)	0.392 (9)
C20B	0.9663 (16)	1.0947 (11)	0.6932 (10)	0.067 (3)	0.392 (9)
H20D	0.929162	1.117404	0.660820	0.100*	0.392 (9)
H20E	0.964711	1.046742	0.678604	0.100*	0.392 (9)
H20F	1.008830	1.113331	0.670687	0.100*	0.392 (9)
N3B	0.9812 (7)	1.1667 (6)	0.8214 (7)	0.080 (2)	0.392 (9)
C21B	0.9905 (15)	1.1761 (12)	0.9321 (12)	0.097 (6)	0.392 (9)
H21D	1.012851	1.218861	0.944549	0.145*	0.392 (9)
H21E	1.017885	1.139457	0.957422	0.145*	0.392 (9)
H21F	0.946656	1.175787	0.963438	0.145*	0.392 (9)
C22B	0.9940 (17)	1.2076 (12)	0.7265 (14)	0.088 (5)	0.392 (9)
H22D	1.026460	1.183676	0.686535	0.131*	0.392 (9)
H22E	1.011672	1.251904	0.742683	0.131*	0.392 (9)
H22F	0.951855	1.212551	0.691363	0.131*	0.392 (9)
Na1	0.750000	0.750000	0.61298 (18)	0.0325 (5)	
O10	0.6805 (12)	1.2775 (16)	0.703 (2)	0.136 (6)	0.275 (7)
C23	0.7332 (14)	1.255 (2)	0.7402 (15)	0.123 (4)	0.275 (7)
C24	0.7931 (15)	1.226 (2)	0.678 (2)	0.129 (6)	0.275 (7)
H24A	0.829103	1.210734	0.720129	0.194*	0.275 (7)
H24B	0.776972	1.187951	0.639447	0.194*	0.275 (7)
H24C	0.809989	1.260828	0.634971	0.194*	0.275 (7)
N4	0.7436 (13)	1.244 (2)	0.8306 (14)	0.128 (4)	0.275 (7)
C25	0.6840 (17)	1.261 (2)	0.897 (2)	0.138 (7)	0.275 (7)
H25A	0.696127	1.251165	0.963847	0.207*	0.275 (7)
H25B	0.673215	1.308695	0.891161	0.207*	0.275 (7)
H25C	0.644929	1.234266	0.879075	0.207*	0.275 (7)
C26	0.8176 (14)	1.2228 (19)	0.852 (2)	0.123 (7)	0.275 (7)
H26A	0.822756	1.214984	0.921383	0.185*	0.275 (7)
H26B	0.828308	1.181623	0.817452	0.185*	0.275 (7)
H26C	0.847942	1.258560	0.832208	0.185*	0.275 (7)
O10B	0.743 (2)	1.2564 (18)	0.6231 (15)	0.131 (6)	0.225 (7)
C23B	0.7383 (13)	1.2528 (11)	0.7142 (16)	0.123 (4)	0.225 (7)
C24B	0.6694 (14)	1.2653 (18)	0.765 (3)	0.135 (6)	0.225 (7)
H24D	0.675022	1.260517	0.834813	0.202*	0.225 (7)
H24E	0.653621	1.310539	0.750689	0.202*	0.225 (7)
H24F	0.636573	1.232511	0.742579	0.202*	0.225 (7)
N4B	0.7880 (11)	1.2390 (11)	0.7703 (16)	0.121 (4)	0.225 (7)
C25B	0.8556 (13)	1.2272 (17)	0.714 (3)	0.130 (8)	0.225 (7)
H25D	0.891378	1.217022	0.759840	0.194*	0.225 (7)
H25E	0.850128	1.189670	0.669709	0.194*	0.225 (7)
H25F	0.867143	1.267689	0.678120	0.194*	0.225 (7)
C26B	0.7712 (18)	1.237 (2)	0.8796 (15)	0.123 (7)	0.225 (7)
H26D	0.811704	1.226485	0.915929	0.185*	0.225 (7)
H26E	0.754256	1.281090	0.899651	0.185*	0.225 (7)
H26F	0.737029	1.203096	0.891637	0.185*	0.225 (7)
Dy1	0.750000	0.750000	0.36449 (2)	0.03521 (10)	

### Tetra-µ-aqua-tetrakis{2-[azanidylene(oxido)methyl]phenolato}tetrakis(µ2-3-hydroxybenzoato)dysprosium(III)tetramanganese(III)sodium(I) N,N-dimethylacetamide decasolvate (1) Atomic displacement parameters (Å^2^)

**Table:**

	*U*^11^	*U*^22^	*U*^33^	*U*^12^	*U*^13^	*U*^23^
Mn1	0.0247 (3)	0.0243 (3)	0.0386 (3)	−0.00064 (18)	−0.00242 (18)	0.00210 (18)
O1	0.0239 (10)	0.0244 (10)	0.0398 (12)	0.0020 (9)	−0.0009 (9)	−0.0025 (9)
N1	0.026 (2)	0.0272 (15)	0.034 (2)	0.0023 (14)	−0.003 (2)	0.0011 (13)
O2	0.0261 (12)	0.0283 (13)	0.0455 (14)	−0.0007 (10)	−0.0043 (10)	0.0002 (11)
C1	0.0283 (14)	0.0273 (14)	0.0345 (14)	0.0006 (12)	−0.0043 (11)	0.0007 (11)
C2	0.0289 (19)	0.0261 (15)	0.0395 (17)	0.0012 (14)	−0.0069 (18)	0.0019 (13)
C3	0.0361 (19)	0.0270 (17)	0.073 (2)	0.0007 (15)	−0.0048 (17)	−0.0010 (17)
C4	0.047 (3)	0.029 (2)	0.093 (4)	0.0036 (19)	−0.009 (2)	−0.008 (2)
C5	0.038 (2)	0.0306 (18)	0.076 (3)	0.0080 (18)	−0.004 (2)	−0.0077 (18)
C6	0.0312 (17)	0.0326 (17)	0.052 (2)	0.0057 (15)	−0.0076 (15)	−0.0068 (15)
C7	0.0294 (15)	0.0298 (15)	0.0387 (16)	0.0026 (13)	−0.0053 (12)	−0.0034 (12)
O3	0.0286 (13)	0.0276 (14)	0.0572 (17)	0.0015 (11)	0.0010 (11)	−0.0065 (13)
O4	0.0432 (15)	0.0422 (15)	0.0427 (15)	−0.0030 (12)	0.0010 (11)	0.0125 (11)
O5	0.0502 (16)	0.0479 (16)	0.0451 (15)	−0.0028 (13)	0.0089 (12)	0.0067 (12)
C8	0.0400 (18)	0.048 (2)	0.0464 (19)	0.0015 (15)	0.0037 (15)	0.0128 (16)
C9	0.054 (2)	0.055 (2)	0.0407 (19)	−0.0028 (18)	0.0067 (17)	0.0172 (17)
C10	0.074 (3)	0.061 (3)	0.045 (2)	−0.015 (2)	−0.010 (2)	0.015 (2)
C11	0.092 (4)	0.069 (3)	0.048 (2)	−0.020 (3)	−0.011 (3)	0.020 (2)
C12	0.102 (4)	0.074 (3)	0.044 (2)	−0.014 (3)	0.002 (3)	0.018 (2)
C13	0.118 (4)	0.083 (4)	0.048 (3)	−0.025 (3)	0.005 (3)	0.005 (2)
C14	0.098 (4)	0.073 (3)	0.048 (2)	−0.021 (3)	0.010 (3)	0.006 (2)
O6	0.172 (7)	0.095 (4)	0.056 (3)	−0.062 (4)	−0.014 (4)	0.030 (3)
O7	0.0342 (19)	0.0380 (19)	0.0434 (16)	0.0042 (15)	−0.0043 (15)	−0.0048 (13)
Mn1B	0.0308 (11)	0.0282 (11)	0.0298 (10)	−0.0005 (8)	0.0006 (7)	0.0018 (7)
O1B	0.027 (4)	0.029 (4)	0.037 (4)	0.004 (3)	−0.001 (3)	−0.001 (3)
N1B	0.025 (4)	0.026 (4)	0.036 (4)	0.006 (4)	0.000 (4)	−0.002 (4)
O2B	0.027 (4)	0.029 (4)	0.044 (4)	−0.001 (4)	−0.004 (3)	0.001 (4)
C1B	0.032 (3)	0.026 (3)	0.039 (3)	0.001 (3)	−0.005 (3)	−0.002 (3)
C2B	0.030 (4)	0.023 (4)	0.043 (4)	0.002 (4)	−0.006 (4)	−0.003 (3)
C3B	0.033 (4)	0.031 (4)	0.056 (4)	0.006 (4)	−0.007 (4)	−0.009 (4)
C4B	0.040 (5)	0.031 (4)	0.073 (5)	0.009 (4)	−0.003 (5)	−0.010 (4)
C5B	0.038 (5)	0.034 (5)	0.076 (5)	0.003 (4)	−0.009 (4)	−0.009 (4)
C6B	0.039 (5)	0.029 (4)	0.068 (5)	−0.001 (4)	−0.005 (4)	−0.005 (4)
C7B	0.035 (4)	0.027 (4)	0.045 (4)	0.001 (3)	−0.003 (3)	0.002 (3)
O3B	0.029 (4)	0.030 (4)	0.041 (4)	0.001 (3)	0.000 (3)	−0.001 (3)
O4B	0.041 (4)	0.044 (4)	0.036 (4)	−0.004 (4)	0.001 (4)	0.008 (4)
O5B	0.053 (5)	0.053 (5)	0.039 (4)	−0.001 (4)	−0.002 (4)	0.011 (4)
C8B	0.048 (4)	0.050 (4)	0.038 (4)	−0.002 (4)	0.003 (3)	0.011 (3)
C9B	0.068 (4)	0.062 (4)	0.043 (4)	−0.012 (4)	0.003 (4)	0.013 (4)
C10B	0.078 (5)	0.067 (5)	0.043 (4)	−0.018 (5)	−0.003 (5)	0.016 (4)
C11B	0.095 (5)	0.074 (5)	0.047 (4)	−0.024 (5)	−0.002 (5)	0.017 (4)
C12B	0.106 (5)	0.081 (5)	0.048 (5)	−0.019 (5)	0.000 (5)	0.014 (5)
C13B	0.103 (5)	0.078 (5)	0.047 (4)	−0.021 (5)	0.000 (5)	0.008 (5)
C14B	0.090 (5)	0.070 (5)	0.044 (4)	−0.019 (5)	0.003 (5)	0.010 (4)
O6B	0.115 (11)	0.090 (10)	0.060 (9)	−0.039 (9)	−0.001 (9)	0.033 (8)
O7B	0.027 (6)	0.036 (6)	0.036 (5)	0.011 (5)	0.001 (5)	0.000 (4)
O8	0.043 (3)	0.041 (3)	0.057 (4)	−0.006 (2)	0.005 (3)	−0.014 (2)
C15	0.102 (5)	0.062 (4)	0.050 (4)	0.011 (4)	−0.002 (4)	−0.004 (3)
C16	0.087 (6)	0.064 (7)	0.056 (6)	−0.022 (5)	−0.015 (5)	0.011 (4)
N2	0.095 (5)	0.048 (3)	0.052 (3)	0.010 (4)	0.005 (4)	−0.004 (3)
C17	0.086 (6)	0.054 (6)	0.052 (6)	0.018 (5)	−0.003 (5)	0.006 (5)
C18	0.170 (11)	0.057 (6)	0.081 (9)	0.009 (7)	−0.012 (9)	−0.020 (5)
O8B	0.149 (9)	0.054 (5)	0.059 (4)	0.023 (6)	−0.008 (7)	−0.018 (4)
C15B	0.112 (5)	0.053 (4)	0.042 (3)	0.016 (4)	0.005 (4)	−0.004 (3)
C16B	0.128 (9)	0.064 (7)	0.066 (8)	0.030 (6)	0.034 (7)	0.027 (5)
N2B	0.120 (5)	0.055 (4)	0.059 (3)	0.007 (4)	−0.001 (4)	−0.016 (3)
C17B	0.140 (9)	0.071 (8)	0.100 (10)	−0.016 (7)	−0.047 (7)	−0.015 (7)
C18B	0.143 (10)	0.042 (5)	0.075 (8)	0.016 (6)	−0.015 (7)	−0.012 (4)
O9	0.106 (8)	0.108 (7)	0.057 (5)	−0.037 (6)	0.009 (4)	0.031 (5)
C19	0.079 (4)	0.095 (5)	0.048 (3)	−0.023 (4)	0.006 (3)	0.009 (3)
C20	0.106 (9)	0.117 (9)	0.067 (5)	−0.011 (7)	0.006 (5)	−0.011 (6)
N3	0.085 (4)	0.083 (4)	0.052 (3)	−0.027 (3)	0.007 (3)	0.009 (3)
C21	0.105 (8)	0.108 (9)	0.094 (8)	−0.010 (7)	0.041 (7)	0.015 (7)
C22	0.105 (9)	0.071 (5)	0.077 (7)	−0.033 (5)	0.007 (6)	0.013 (5)
O9B	0.091 (9)	0.098 (8)	0.058 (7)	−0.004 (6)	0.008 (6)	0.016 (6)
C19B	0.079 (5)	0.083 (5)	0.063 (5)	−0.023 (4)	−0.001 (4)	0.012 (4)
C20B	0.083 (8)	0.071 (7)	0.046 (5)	−0.032 (6)	0.023 (6)	0.007 (5)
N3B	0.087 (5)	0.092 (5)	0.061 (5)	−0.020 (4)	0.003 (4)	0.007 (4)
C21B	0.099 (11)	0.116 (11)	0.076 (8)	−0.023 (9)	0.000 (8)	0.003 (8)
C22B	0.105 (11)	0.088 (8)	0.070 (9)	−0.034 (8)	0.011 (8)	−0.001 (7)
Na1	0.0267 (6)	0.0267 (6)	0.0442 (12)	0.000	0.000	0.000
O10	0.131 (10)	0.120 (9)	0.158 (10)	−0.006 (9)	−0.003 (9)	−0.004 (9)
C23	0.125 (7)	0.106 (6)	0.138 (6)	−0.015 (6)	−0.009 (6)	0.002 (6)
C24	0.130 (10)	0.118 (9)	0.140 (10)	−0.020 (9)	−0.019 (9)	0.014 (9)
N4	0.130 (7)	0.115 (6)	0.141 (6)	−0.012 (5)	−0.012 (6)	−0.002 (6)
C25	0.139 (11)	0.132 (11)	0.143 (11)	0.005 (10)	−0.015 (10)	0.000 (10)
C26	0.133 (11)	0.117 (11)	0.119 (11)	−0.011 (10)	−0.026 (10)	−0.003 (10)
O10B	0.121 (10)	0.111 (9)	0.160 (10)	−0.028 (8)	−0.011 (10)	0.006 (10)
C23B	0.124 (7)	0.107 (6)	0.137 (6)	−0.014 (6)	−0.010 (6)	0.001 (6)
C24B	0.137 (10)	0.122 (9)	0.145 (10)	−0.005 (9)	−0.004 (9)	0.000 (9)
N4B	0.124 (7)	0.110 (6)	0.130 (7)	−0.013 (6)	−0.009 (5)	0.000 (6)
C25B	0.125 (13)	0.121 (12)	0.142 (13)	−0.008 (12)	−0.009 (12)	0.002 (12)
C26B	0.131 (11)	0.120 (10)	0.119 (10)	−0.012 (10)	−0.027 (9)	0.001 (9)
Dy1	0.03543 (12)	0.03543 (12)	0.03478 (15)	0.000	0.000	0.000

### Tetra-µ-aqua-tetrakis{2-[azanidylene(oxido)methyl]phenolato}tetrakis(µ2-3-hydroxybenzoato)dysprosium(III)tetramanganese(III)sodium(I) N,N-dimethylacetamide decasolvate (1) Geometric parameters (Å, º)

**Table:**

Mn1—O3^i^	1.854 (3)	O8—C15	1.245 (10)
Mn1—O1	1.916 (2)	C15—N2	1.295 (10)
Mn1—O2	1.951 (3)	C15—C16	1.557 (13)
Mn1—N1^i^	1.974 (7)	C16—H16A	0.9800
Mn1—O4	2.175 (3)	C16—H16B	0.9800
Mn1—O7	2.448 (4)	C16—H16C	0.9800
Mn1—Na1	3.6274 (12)	N2—C17	1.548 (12)
O1—N1	1.399 (6)	N2—C18	1.599 (11)
O1—Dy1	2.417 (2)	C17—H17A	0.9800
O1—Na1	2.673 (3)	C17—H17B	0.9800
N1—C1	1.314 (7)	C17—H17C	0.9800
O2—C1	1.293 (4)	C18—H18A	0.9800
C1—C2	1.470 (5)	C18—H18B	0.9800
C2—C3	1.398 (6)	C18—H18C	0.9800
C2—C7	1.408 (5)	O8B—C15B	1.248 (10)
C3—C4	1.384 (7)	C15B—N2B	1.345 (10)
C3—H3	0.9500	C15B—C16B	1.554 (14)
C4—C5	1.396 (7)	C16B—H16D	0.9800
C4—H4	0.9500	C16B—H16E	0.9800
C5—C6	1.377 (7)	C16B—H16F	0.9800
C5—H5	0.9500	N2B—C17B	1.500 (13)
C6—C7	1.406 (5)	N2B—C18B	1.632 (11)
C6—H6	0.9500	C17B—H17D	0.9800
C7—O3	1.338 (5)	C17B—H17E	0.9800
O4—C8	1.245 (6)	C17B—H17F	0.9800
O5—C8	1.273 (5)	C18B—H18D	0.9800
O5—Dy1	2.261 (3)	C18B—H18E	0.9800
C8—C9	1.512 (6)	C18B—H18F	0.9800
C9—C10	1.354 (7)	O9—C19	1.241 (10)
C9—C14	1.393 (8)	C19—N3	1.323 (8)
C10—C11	1.396 (7)	C19—C20	1.561 (12)
C10—H10	0.9500	C20—H20A	0.9800
C11—O6	1.348 (7)	C20—H20B	0.9800
C11—C12	1.386 (8)	C20—H20C	0.9800
C12—C13	1.373 (9)	N3—C22	1.548 (10)
C12—H12	0.9500	N3—C21	1.565 (12)
C13—C14	1.400 (8)	C21—H21A	0.9800
C13—H13	0.9500	C21—H21B	0.9800
C14—H14	0.9500	C21—H21C	0.9800
O6—H6A1	0.8401	C22—H22A	0.9800
O7—Na1	2.422 (4)	C22—H22B	0.9800
O7—H7A	0.8243	C22—H22C	0.9800
O7—H7B	0.8155	O9B—C19B	1.228 (13)
Mn1B—O3B	1.848 (12)	C19B—N3B	1.331 (12)
Mn1B—O1B^i^	1.915 (10)	C19B—C20B	1.539 (14)
Mn1B—O2B^i^	1.970 (13)	C20B—H20D	0.9800
Mn1B—N1B	2.00 (3)	C20B—H20E	0.9800
Mn1B—O4B	2.171 (10)	C20B—H20F	0.9800
Mn1B—O7B	2.471 (15)	N3B—C21B	1.568 (13)
Mn1B—Na1	3.597 (2)	N3B—C22B	1.576 (12)
O1B—N1B	1.402 (18)	C21B—H21D	0.9800
O1B—Dy1	2.460 (9)	C21B—H21E	0.9800
O1B—Na1	2.637 (10)	C21B—H21F	0.9800
N1B—C1B	1.316 (19)	C22B—H22D	0.9800
O2B—C1B	1.277 (13)	C22B—H22E	0.9800
C1B—C2B	1.480 (14)	C22B—H22F	0.9800
C2B—C3B	1.389 (15)	Na1—H7D	2.5822
C2B—C7B	1.412 (14)	O10—C23	1.262 (14)
C3B—C4B	1.402 (18)	C23—N4	1.297 (14)
C3B—H3B	0.9500	C23—C24	1.590 (17)
C4B—C5B	1.384 (17)	C24—H24A	0.9800
C4B—H4B	0.9500	C24—H24B	0.9800
C5B—C6B	1.378 (18)	C24—H24C	0.9800
C5B—H5B	0.9500	N4—C25	1.552 (14)
C6B—C7B	1.411 (14)	N4—C26	1.567 (14)
C6B—H6B	0.9500	C25—H25A	0.9800
C7B—O3B	1.357 (13)	C25—H25B	0.9800
O4B—C8B	1.240 (14)	C25—H25C	0.9800
O5B—C8B	1.272 (14)	C26—H26A	0.9800
O5B—Dy1	2.222 (11)	C26—H26B	0.9800
C8B—C9B	1.513 (15)	C26—H26C	0.9800
C9B—C10B	1.375 (16)	O10B—C23B	1.277 (15)
C9B—C14B	1.407 (17)	C23B—N4B	1.293 (15)
C10B—C11B	1.404 (17)	C23B—C24B	1.572 (17)
C10B—H10B	0.9500	C24B—H24D	0.9800
C11B—O6B	1.353 (17)	C24B—H24E	0.9800
C11B—C12B	1.395 (17)	C24B—H24F	0.9800
C12B—C13B	1.373 (18)	N4B—C26B	1.562 (14)
C12B—H12B	0.9500	N4B—C25B	1.580 (14)
C13B—C14B	1.397 (17)	C25B—H25D	0.9800
C13B—H13B	0.9500	C25B—H25E	0.9800
C14B—H14B	0.9500	C25B—H25F	0.9800
O6B—H6B1	0.8400	C26B—H26D	0.9800
O7B—Na1	2.444 (16)	C26B—H26E	0.9800
O7B—H7C	0.8387	C26B—H26F	0.9800
O7B—H7D	0.8449		
			
O3^i^—Mn1—O1	171.06 (13)	O9—C19—C20	123.3 (10)
O3^i^—Mn1—O2	97.51 (12)	N3—C19—C20	112.5 (8)
O1—Mn1—O2	81.64 (10)	C19—C20—H20A	109.5
O3^i^—Mn1—N1^i^	90.50 (16)	C19—C20—H20B	109.5
O1—Mn1—N1^i^	89.09 (16)	H20A—C20—H20B	109.5
O2—Mn1—N1^i^	168.1 (4)	C19—C20—H20C	109.5
O3^i^—Mn1—O4	95.15 (13)	H20A—C20—H20C	109.5
O1—Mn1—O4	93.76 (11)	H20B—C20—H20C	109.5
O2—Mn1—O4	90.56 (12)	C19—N3—C22	116.3 (8)
N1^i^—Mn1—O4	97.5 (6)	C19—N3—C21	109.8 (7)
O3^i^—Mn1—O7	91.12 (14)	C22—N3—C21	132.4 (10)
O1—Mn1—O7	79.96 (12)	N3—C21—H21A	109.5
O2—Mn1—O7	87.86 (12)	N3—C21—H21B	109.5
N1^i^—Mn1—O7	83.2 (6)	H21A—C21—H21B	109.5
O4—Mn1—O7	173.68 (12)	N3—C21—H21C	109.5
O3^i^—Mn1—Na1	126.39 (11)	H21A—C21—H21C	109.5
O1—Mn1—Na1	45.84 (8)	H21B—C21—H21C	109.5
O2—Mn1—Na1	102.65 (8)	N3—C22—H22A	109.5
N1^i^—Mn1—Na1	65.5 (4)	N3—C22—H22B	109.5
O4—Mn1—Na1	133.19 (9)	H22A—C22—H22B	109.5
O7—Mn1—Na1	41.59 (10)	N3—C22—H22C	109.5
N1—O1—Mn1	112.5 (4)	H22A—C22—H22C	109.5
N1—O1—Dy1	121.5 (6)	H22B—C22—H22C	109.5
Mn1—O1—Dy1	120.38 (11)	O9B—C19B—N3B	119.2 (14)
N1—O1—Na1	105.6 (7)	O9B—C19B—C20B	133.4 (14)
Mn1—O1—Na1	103.22 (10)	N3B—C19B—C20B	107.4 (10)
Dy1—O1—Na1	85.74 (8)	C19B—C20B—H20D	109.5
C1—N1—O1	113.4 (6)	C19B—C20B—H20E	109.5
C1—N1—Mn1^ii^	129.9 (4)	H20D—C20B—H20E	109.5
O1—N1—Mn1^ii^	115.5 (3)	C19B—C20B—H20F	109.5
C1—O2—Mn1	112.0 (2)	H20D—C20B—H20F	109.5
O2—C1—N1	120.3 (4)	H20E—C20B—H20F	109.5
O2—C1—C2	119.6 (3)	C19B—N3B—C21B	110.3 (10)
N1—C1—C2	120.1 (4)	C19B—N3B—C22B	111.2 (10)
C3—C2—C7	119.0 (4)	C21B—N3B—C22B	138.3 (14)
C3—C2—C1	118.0 (4)	N3B—C21B—H21D	109.5
C7—C2—C1	122.9 (3)	N3B—C21B—H21E	109.5
C4—C3—C2	121.8 (4)	H21D—C21B—H21E	109.5
C4—C3—H3	119.1	N3B—C21B—H21F	109.5
C2—C3—H3	119.1	H21D—C21B—H21F	109.5
C3—C4—C5	118.9 (5)	H21E—C21B—H21F	109.5
C3—C4—H4	120.6	N3B—C22B—H22D	109.5
C5—C4—H4	120.6	N3B—C22B—H22E	109.5
C6—C5—C4	120.4 (5)	H22D—C22B—H22E	109.5
C6—C5—H5	119.8	N3B—C22B—H22F	109.5
C4—C5—H5	119.8	H22D—C22B—H22F	109.5
C5—C6—C7	121.1 (4)	H22E—C22B—H22F	109.5
C5—C6—H6	119.5	O7—Na1—O7^iii^	147.5 (2)
C7—C6—H6	119.5	O7—Na1—O7^i^	85.52 (6)
O3—C7—C6	117.3 (3)	O7^iii^—Na1—O7^i^	85.52 (6)
O3—C7—C2	123.9 (3)	O7—Na1—O7^ii^	85.52 (6)
C6—C7—C2	118.8 (3)	O7^iii^—Na1—O7^ii^	85.52 (6)
C7—O3—Mn1^ii^	129.9 (3)	O7^i^—Na1—O7^ii^	147.5 (2)
C8—O4—Mn1	123.4 (3)	O7—Na1—O7B^iii^	142.8 (3)
C8—O5—Dy1	142.0 (3)	O7^iii^—Na1—O7B^iii^	13.4 (2)
O4—C8—O5	124.6 (4)	O7^i^—Na1—O7B^iii^	72.2 (3)
O4—C8—C9	119.6 (4)	O7^ii^—Na1—O7B^iii^	97.5 (3)
O5—C8—C9	115.8 (4)	O7B—Na1—O7B^iii^	143.3 (7)
C10—C9—C14	120.2 (4)	O7—Na1—O7B^i^	72.2 (3)
C10—C9—C8	120.3 (5)	O7^iii^—Na1—O7B^i^	97.5 (3)
C14—C9—C8	119.4 (4)	O7^i^—Na1—O7B^i^	13.4 (2)
C9—C10—C11	119.4 (5)	O7^ii^—Na1—O7B^i^	142.8 (3)
C9—C10—H10	120.3	O7B—Na1—O7B^i^	84.3 (2)
C11—C10—H10	120.3	O7B^iii^—Na1—O7B^i^	84.3 (2)
O6—C11—C12	121.0 (5)	O7—Na1—O7B^ii^	97.5 (3)
O6—C11—C10	117.9 (6)	O7^iii^—Na1—O7B^ii^	72.2 (3)
C12—C11—C10	121.0 (5)	O7^i^—Na1—O7B^ii^	142.8 (3)
C13—C12—C11	119.6 (5)	O7^ii^—Na1—O7B^ii^	13.4 (2)
C13—C12—H12	120.2	O7B—Na1—O7B^ii^	84.3 (2)
C11—C12—H12	120.2	O7B^iii^—Na1—O7B^ii^	84.3 (2)
C12—C13—C14	119.2 (6)	O7B^i^—Na1—O7B^ii^	143.3 (7)
C12—C13—H13	120.4	O7B—Na1—O1B	85.9 (4)
C14—C13—H13	120.4	O7B^iii^—Na1—O1B	121.1 (4)
C9—C14—C13	120.4 (6)	O7B^i^—Na1—O1B	145.7 (4)
C9—C14—H14	119.8	O7B^ii^—Na1—O1B	67.6 (4)
C13—C14—H14	119.8	O7—Na1—O1B^ii^	149.5 (2)
C11—O6—H6A1	109.9	O7^iii^—Na1—O1B^ii^	62.2 (2)
Na1—O7—Mn1	96.27 (14)	O7^i^—Na1—O1B^ii^	109.9 (2)
Na1—O7—H7A	105.4	O7^ii^—Na1—O1B^ii^	93.2 (2)
Mn1—O7—H7A	105.7	O7B—Na1—O1B^ii^	145.7 (4)
Na1—O7—H7B	113.8	O7B^iii^—Na1—O1B^ii^	67.6 (4)
Mn1—O7—H7B	121.3	O7B^i^—Na1—O1B^ii^	121.1 (4)
H7A—O7—H7B	112.4	O7B^ii^—Na1—O1B^ii^	85.9 (4)
O3B—Mn1B—O1B^i^	171.8 (5)	O1B—Na1—O1B^ii^	60.0 (2)
O3B—Mn1B—N1B	90.3 (5)	O7—Na1—O1B^iii^	109.9 (2)
O1B^i^—Mn1B—N1B	89.4 (6)	O7^iii^—Na1—O1B^iii^	93.2 (2)
O2B^i^—Mn1B—N1B	169.6 (16)	O7^i^—Na1—O1B^iii^	62.2 (2)
O3B—Mn1B—O4B	94.6 (5)	O7^ii^—Na1—O1B^iii^	149.5 (2)
O1B^i^—Mn1B—O4B	93.6 (5)	O7B—Na1—O1B^iii^	121.1 (4)
O2B^i^—Mn1B—O4B	91.4 (5)	O7B^iii^—Na1—O1B^iii^	85.9 (4)
N1B—Mn1B—O4B	95 (3)	O7B^i^—Na1—O1B^iii^	67.6 (4)
O3B—Mn1B—O7B	92.6 (5)	O7B^ii^—Na1—O1B^iii^	145.7 (4)
O1B^i^—Mn1B—O7B	79.3 (5)	O1B—Na1—O1B^iii^	90.0 (4)
O2B^i^—Mn1B—O7B	86.2 (5)	O1B^ii^—Na1—O1B^iii^	60.0 (2)
N1B—Mn1B—O7B	86 (3)	O7—Na1—O1B^i^	62.2 (2)
O4B—Mn1B—O7B	172.7 (5)	O7^iii^—Na1—O1B^i^	149.5 (2)
O3B—Mn1B—Na1	127.4 (4)	O7^i^—Na1—O1B^i^	93.2 (2)
O1B^i^—Mn1B—Na1	45.5 (3)	O7^ii^—Na1—O1B^i^	109.9 (2)
O2B^i^—Mn1B—Na1	103.6 (3)	O7B—Na1—O1B^i^	67.6 (4)
N1B—Mn1B—Na1	66.1 (18)	O7B^iii^—Na1—O1B^i^	145.7 (4)
O4B—Mn1B—Na1	131.9 (3)	O7B^i^—Na1—O1B^i^	85.9 (4)
O7B—Mn1B—Na1	42.7 (4)	O7B^ii^—Na1—O1B^i^	121.1 (4)
N1B—O1B—Mn1B^ii^	111.7 (12)	O1B—Na1—O1B^i^	60.0 (2)
N1B—O1B—Dy1	121 (2)	O1B^ii^—Na1—O1B^i^	90.0 (4)
Mn1B^ii^—O1B—Dy1	120.6 (5)	O1B^iii^—Na1—O1B^i^	60.0 (2)
N1B—O1B—Na1	107 (3)	O7B—Na1—H7D	19.1
Mn1B^ii^—O1B—Na1	103.3 (4)	O7B^iii^—Na1—H7D	135.9
Dy1—O1B—Na1	85.7 (3)	O7B^i^—Na1—H7D	65.3
C1B—N1B—O1B	114 (2)	O7B^ii^—Na1—H7D	101.0
C1B—N1B—Mn1B	130 (2)	O1B—Na1—H7D	100.8
O1B—N1B—Mn1B	114.2 (17)	O1B^ii^—Na1—H7D	155.7
C1B—O2B—Mn1B^ii^	111.0 (9)	O1B^iii^—Na1—H7D	108.8
O2B—C1B—N1B	121.1 (15)	O1B^i^—Na1—H7D	66.4
O2B—C1B—C2B	119.0 (11)	O10—C23—N4	126.8 (19)
N1B—C1B—C2B	119.8 (14)	O10—C23—C24	122.2 (19)
C3B—C2B—C7B	120.0 (12)	N4—C23—C24	110.5 (16)
C3B—C2B—C1B	118.2 (12)	C23—C24—H24A	109.5
C7B—C2B—C1B	121.8 (11)	C23—C24—H24B	109.5
C2B—C3B—C4B	119.8 (14)	H24A—C24—H24B	109.5
C2B—C3B—H3B	120.1	C23—C24—H24C	109.5
C4B—C3B—H3B	120.1	H24A—C24—H24C	109.5
C5B—C4B—C3B	120.0 (18)	H24B—C24—H24C	109.5
C5B—C4B—H4B	120.0	C23—N4—C25	115.0 (13)
C3B—C4B—H4B	120.0	C23—N4—C26	112.7 (12)
C6B—C5B—C4B	119.3 (17)	C25—N4—C26	131.9 (16)
C6B—C5B—H5B	120.4	N4—C25—H25A	109.5
C4B—C5B—H5B	120.4	N4—C25—H25B	109.5
C5B—C6B—C7B	121.2 (14)	H25A—C25—H25B	109.5
C5B—C6B—H6B	119.4	N4—C25—H25C	109.5
C7B—C6B—H6B	119.4	H25A—C25—H25C	109.5
O3B—C7B—C6B	116.5 (12)	H25B—C25—H25C	109.5
O3B—C7B—C2B	125.1 (11)	N4—C26—H26A	109.5
C6B—C7B—C2B	118.3 (11)	N4—C26—H26B	109.5
C7B—O3B—Mn1B	129.0 (9)	H26A—C26—H26B	109.5
C8B—O4B—Mn1B	123.7 (9)	N4—C26—H26C	109.5
C8B—O5B—Dy1	143.9 (11)	H26A—C26—H26C	109.5
O4B—C8B—O5B	125.8 (13)	H26B—C26—H26C	109.5
O4B—C8B—C9B	119.0 (12)	O10B—C23B—N4B	124 (2)
O5B—C8B—C9B	115.1 (12)	O10B—C23B—C24B	121 (2)
C10B—C9B—C14B	120.2 (13)	N4B—C23B—C24B	115.5 (18)
C10B—C9B—C8B	119.8 (13)	C23B—C24B—H24D	109.5
C14B—C9B—C8B	120.0 (13)	C23B—C24B—H24E	109.5
C9B—C10B—C11B	120.2 (16)	H24D—C24B—H24E	109.5
C9B—C10B—H10B	119.9	C23B—C24B—H24F	109.5
C11B—C10B—H10B	119.9	H24D—C24B—H24F	109.5
O6B—C11B—C12B	122 (2)	H24E—C24B—H24F	109.5
O6B—C11B—C10B	115.0 (19)	C23B—N4B—C26B	115.5 (12)
C12B—C11B—C10B	118.5 (16)	C23B—N4B—C25B	112.8 (12)
C13B—C12B—C11B	121.0 (17)	C26B—N4B—C25B	131.6 (17)
C13B—C12B—H12B	119.5	N4B—C25B—H25D	109.5
C11B—C12B—H12B	119.5	N4B—C25B—H25E	109.5
C12B—C13B—C14B	119.9 (17)	H25D—C25B—H25E	109.5
C12B—C13B—H13B	120.0	N4B—C25B—H25F	109.5
C14B—C13B—H13B	120.0	H25D—C25B—H25F	109.5
C13B—C14B—C9B	119.2 (16)	H25E—C25B—H25F	109.5
C13B—C14B—H14B	120.4	N4B—C26B—H26D	109.5
C9B—C14B—H14B	120.4	N4B—C26B—H26E	109.5
C11B—O6B—H6B1	107.1	H26D—C26B—H26E	109.5
Na1—O7B—Mn1B	94.1 (5)	N4B—C26B—H26F	109.5
Na1—O7B—H7C	141.1	H26D—C26B—H26F	109.5
Mn1B—O7B—H7C	112.8	H26E—C26B—H26F	109.5
Na1—O7B—H7D	89.7	O5B—Dy1—O5B^iii^	126.8 (7)
Mn1B—O7B—H7D	106.5	O5B—Dy1—O5B^ii^	78.4 (3)
H7C—O7B—H7D	107.6	O5B^iii^—Dy1—O5B^ii^	78.4 (3)
O8—C15—N2	120.5 (9)	O5B—Dy1—O5^iii^	109.5 (3)
O8—C15—C16	125.1 (10)	O5B^iii^—Dy1—O5^iii^	41.3 (3)
N2—C15—C16	114.3 (9)	O5B^ii^—Dy1—O5^iii^	38.3 (3)
C15—C16—H16A	109.5	O5B^i^—Dy1—O5^iii^	111.5 (4)
C15—C16—H16B	109.5	O5—Dy1—O5^iii^	124.37 (17)
H16A—C16—H16B	109.5	O5—Dy1—O5^i^	77.42 (7)
C15—C16—H16C	109.5	O5^iii^—Dy1—O5^i^	77.42 (7)
H16A—C16—H16C	109.5	O5B—Dy1—O5^ii^	38.3 (3)
H16B—C16—H16C	109.5	O5B^iii^—Dy1—O5^ii^	111.5 (4)
C15—N2—C17	112.8 (8)	O5B^ii^—Dy1—O5^ii^	41.3 (3)
C15—N2—C18	109.6 (9)	O5B^i^—Dy1—O5^ii^	109.5 (3)
C17—N2—C18	137.3 (11)	O5—Dy1—O5^ii^	77.42 (7)
N2—C17—H17A	109.5	O5^iii^—Dy1—O5^ii^	77.42 (7)
N2—C17—H17B	109.5	O5^i^—Dy1—O5^ii^	124.37 (17)
H17A—C17—H17B	109.5	O5B—Dy1—O1^i^	108.9 (3)
N2—C17—H17C	109.5	O5B^iii^—Dy1—O1^i^	104.4 (4)
H17A—C17—H17C	109.5	O5B^ii^—Dy1—O1^i^	166.6 (3)
H17B—C17—H17C	109.5	O5B^i^—Dy1—O1^i^	66.4 (3)
N2—C18—H18A	109.5	O5—Dy1—O1^i^	80.22 (10)
N2—C18—H18B	109.5	O5^iii^—Dy1—O1^i^	140.07 (10)
H18A—C18—H18B	109.5	O5^i^—Dy1—O1^i^	78.80 (10)
N2—C18—H18C	109.5	O5^ii^—Dy1—O1^i^	142.30 (10)
H18A—C18—H18C	109.5	O5B—Dy1—O1^ii^	104.4 (3)
H18B—C18—H18C	109.5	O5B^iii^—Dy1—O1^ii^	108.9 (3)
O8B—C15B—N2B	126.1 (10)	O5B^ii^—Dy1—O1^ii^	66.4 (3)
O8B—C15B—C16B	116.0 (11)	O5B^i^—Dy1—O1^ii^	166.6 (3)
N2B—C15B—C16B	117.8 (10)	O5—Dy1—O1^ii^	140.07 (10)
C15B—C16B—H16D	109.5	O5^iii^—Dy1—O1^ii^	80.22 (10)
C15B—C16B—H16E	109.5	O5^i^—Dy1—O1^ii^	142.30 (10)
H16D—C16B—H16E	109.5	O5^ii^—Dy1—O1^ii^	78.80 (10)
C15B—C16B—H16F	109.5	O1^i^—Dy1—O1^ii^	100.46 (12)
H16D—C16B—H16F	109.5	O5B—Dy1—O1^iii^	166.6 (3)
H16E—C16B—H16F	109.5	O5B^iii^—Dy1—O1^iii^	66.4 (3)
C15B—N2B—C17B	115.0 (9)	O5B^ii^—Dy1—O1^iii^	104.4 (3)
C15B—N2B—C18B	105.0 (8)	O5B^i^—Dy1—O1^iii^	108.9 (3)
C17B—N2B—C18B	138.8 (11)	O5—Dy1—O1^iii^	142.30 (10)
N2B—C17B—H17D	109.5	O5^iii^—Dy1—O1^iii^	78.80 (10)
N2B—C17B—H17E	109.5	O5^i^—Dy1—O1^iii^	80.22 (10)
H17D—C17B—H17E	109.5	O5^ii^—Dy1—O1^iii^	140.07 (10)
N2B—C17B—H17F	109.5	O1^i^—Dy1—O1^iii^	65.84 (6)
H17D—C17B—H17F	109.5	O1^ii^—Dy1—O1^iii^	65.84 (6)
H17E—C17B—H17F	109.5	O5—Dy1—O1	78.80 (10)
N2B—C18B—H18D	109.5	O5^iii^—Dy1—O1	142.30 (10)
N2B—C18B—H18E	109.5	O5^i^—Dy1—O1	140.07 (10)
H18D—C18B—H18E	109.5	O5^ii^—Dy1—O1	80.22 (10)
N2B—C18B—H18F	109.5	O1^i^—Dy1—O1	65.84 (6)
H18D—C18B—H18F	109.5	O1^ii^—Dy1—O1	65.84 (6)
H18E—C18B—H18F	109.5	O1^iii^—Dy1—O1	100.46 (12)
O9—C19—N3	122.1 (9)		
			
Mn1—O1—N1—C1	3.0 (17)	N1B—C1B—C2B—C7B	−15 (5)
Dy1—O1—N1—C1	−150.5 (9)	C7B—C2B—C3B—C4B	5 (4)
Na1—O1—N1—C1	114.9 (12)	C1B—C2B—C3B—C4B	−175 (2)
Mn1—O1—N1—Mn1^ii^	−165.5 (7)	C2B—C3B—C4B—C5B	−12 (4)
Dy1—O1—N1—Mn1^ii^	41.0 (15)	C3B—C4B—C5B—C6B	15 (5)
Na1—O1—N1—Mn1^ii^	−53.6 (12)	C4B—C5B—C6B—C7B	−11 (4)
Mn1—O2—C1—N1	−2.9 (11)	C5B—C6B—C7B—O3B	−172 (2)
Mn1—O2—C1—C2	177.9 (3)	C5B—C6B—C7B—C2B	4 (3)
O1—N1—C1—O2	0.0 (18)	C3B—C2B—C7B—O3B	175.2 (18)
Mn1^ii^—N1—C1—O2	166.4 (10)	C1B—C2B—C7B—O3B	−5 (3)
O1—N1—C1—C2	179.1 (8)	C3B—C2B—C7B—C6B	−1 (3)
Mn1^ii^—N1—C1—C2	−14 (2)	C1B—C2B—C7B—C6B	178.5 (19)
O2—C1—C2—C3	14.2 (6)	C6B—C7B—O3B—Mn1B	−162.8 (13)
N1—C1—C2—C3	−165.0 (11)	C2B—C7B—O3B—Mn1B	21 (3)
O2—C1—C2—C7	−167.8 (4)	O2B^i^—Mn1B—O3B—C7B	173.3 (13)
N1—C1—C2—C7	13.0 (12)	N1B—Mn1B—O3B—C7B	−14 (3)
C7—C2—C3—C4	−0.5 (8)	O4B—Mn1B—O3B—C7B	81.3 (13)
C1—C2—C3—C4	177.6 (5)	O7B—Mn1B—O3B—C7B	−100.2 (13)
C2—C3—C4—C5	2.9 (10)	Na1—Mn1B—O3B—C7B	−73.4 (13)
C3—C4—C5—C6	−3.2 (11)	Mn1B—O4B—C8B—O5B	1 (3)
C4—C5—C6—C7	1.1 (10)	Mn1B—O4B—C8B—C9B	−175.2 (14)
C5—C6—C7—O3	179.8 (5)	Dy1—O5B—C8B—O4B	−46 (3)
C5—C6—C7—C2	1.3 (7)	Dy1—O5B—C8B—C9B	131.2 (18)
C3—C2—C7—O3	−180.0 (4)	O4B—C8B—C9B—C10B	−4 (3)
C1—C2—C7—O3	2.0 (7)	O5B—C8B—C9B—C10B	179 (2)
C3—C2—C7—C6	−1.6 (7)	O4B—C8B—C9B—C14B	175 (2)
C1—C2—C7—C6	−179.6 (4)	O5B—C8B—C9B—C14B	−2 (3)
C6—C7—O3—Mn1^ii^	165.2 (3)	C14B—C9B—C10B—C11B	−2 (4)
C2—C7—O3—Mn1^ii^	−16.3 (6)	C8B—C9B—C10B—C11B	178 (3)
Mn1—O4—C8—O5	−5.4 (6)	C9B—C10B—C11B—O6B	166 (3)
Mn1—O4—C8—C9	176.3 (3)	C9B—C10B—C11B—C12B	10 (5)
Dy1—O5—C8—O4	50.8 (7)	O6B—C11B—C12B—C13B	−166 (3)
Dy1—O5—C8—C9	−130.8 (4)	C10B—C11B—C12B—C13B	−11 (5)
O4—C8—C9—C10	0.2 (7)	C11B—C12B—C13B—C14B	4 (6)
O5—C8—C9—C10	−178.3 (5)	C12B—C13B—C14B—C9B	4 (5)
O4—C8—C9—C14	−178.6 (5)	C10B—C9B—C14B—C13B	−6 (4)
O5—C8—C9—C14	3.0 (7)	C8B—C9B—C14B—C13B	175 (3)
C14—C9—C10—C11	−2.1 (10)	O8—C15—N2—C17	−0.2 (15)
C8—C9—C10—C11	179.2 (6)	C16—C15—N2—C17	176.2 (10)
C9—C10—C11—O6	−179.5 (7)	O8—C15—N2—C18	174.7 (13)
C9—C10—C11—C12	3.2 (11)	C16—C15—N2—C18	−9.0 (15)
O6—C11—C12—C13	−178.4 (9)	O8B—C15B—N2B—C17B	1.5 (18)
C10—C11—C12—C13	−1.2 (13)	C16B—C15B—N2B—C17B	−174.2 (13)
C11—C12—C13—C14	−1.8 (14)	O8B—C15B—N2B—C18B	171.6 (13)
C10—C9—C14—C13	−0.9 (11)	C16B—C15B—N2B—C18B	−4.1 (14)
C8—C9—C14—C13	177.8 (7)	O9—C19—N3—C22	174.0 (15)
C12—C13—C14—C9	2.9 (13)	C20—C19—N3—C22	9.9 (16)
Mn1B^ii^—O1B—N1B—C1B	−1 (7)	O9—C19—N3—C21	−18.4 (19)
Dy1—O1B—N1B—C1B	151 (4)	C20—C19—N3—C21	177.4 (14)
Na1—O1B—N1B—C1B	−113 (6)	O9B—C19B—N3B—C21B	14 (3)
Mn1B^ii^—O1B—N1B—Mn1B	164 (3)	C20B—C19B—N3B—C21B	−168 (2)
Dy1—O1B—N1B—Mn1B	−44 (6)	O9B—C19B—N3B—C22B	−170 (2)
Na1—O1B—N1B—Mn1B	51 (5)	C20B—C19B—N3B—C22B	8 (3)
Mn1B^ii^—O2B—C1B—N1B	6 (5)	O10—C23—N4—C25	0 (7)
Mn1B^ii^—O2B—C1B—C2B	−178.8 (14)	C24—C23—N4—C25	−172 (3)
O1B—N1B—C1B—O2B	−3 (8)	O10—C23—N4—C26	−174 (4)
Mn1B—N1B—C1B—O2B	−165 (4)	C24—C23—N4—C26	15 (5)
O1B—N1B—C1B—C2B	−179 (4)	O10B—C23B—N4B—C26B	179.9 (3)
Mn1B—N1B—C1B—C2B	20 (9)	C24B—C23B—N4B—C26B	−0.2 (6)
O2B—C1B—C2B—C3B	−10 (3)	O10B—C23B—N4B—C25B	0.2 (6)
N1B—C1B—C2B—C3B	165 (5)	C24B—C23B—N4B—C25B	−179.9 (3)
O2B—C1B—C2B—C7B	170.1 (18)		

Symmetry codes: (i) *y*, −*x*+3/2, *z*; (ii) −*y*+3/2, *x*, *z*; (iii) −*x*+3/2, −*y*+3/2, *z*.

### Tetra-µ-aqua-tetrakis{2-[azanidylene(oxido)methyl]phenolato}tetrakis(µ2-3-hydroxybenzoato)dysprosium(III)tetramanganese(III)sodium(I) N,N-dimethylacetamide decasolvate (1) Hydrogen-bond geometry (Å, º)

**Table:**

*D*—H···*A*	*D*—H	H···*A*	*D*···*A*	*D*—H···*A*
O6—H6*A*1···O9^iv^	0.84	1.82	2.542 (13)	143
O7—H7*A*···O8	0.82	1.85	2.653 (9)	165
O7—H7*B*···O8^i^	0.82	2.05	2.785 (9)	151
C17—H17*B*···O6^v^	0.98	2.53	3.348 (19)	141

Symmetry codes: (i) *y*, −*x*+3/2, *z*; (iv) *x*, *y*, *z*−1; (v) *y*−1/2, −*x*+2, −*z*+1.
